# Supplementary material for: A prebiotic dietary pilot intervention restores faecal metabolites and may be neuroprotective in Parkinson’s Disease
Source: NPJ Parkinsons Dis. 2025 Apr 4;11:66. doi: 10.1038/s41531-025-00885-5 (PMC11968880; doi:10.1038/s41531-025-00885-5)
Supplement: Supplementary file 1 — Supplementary Information [file 41531_2025_885_MOESM1_ESM.pdf]

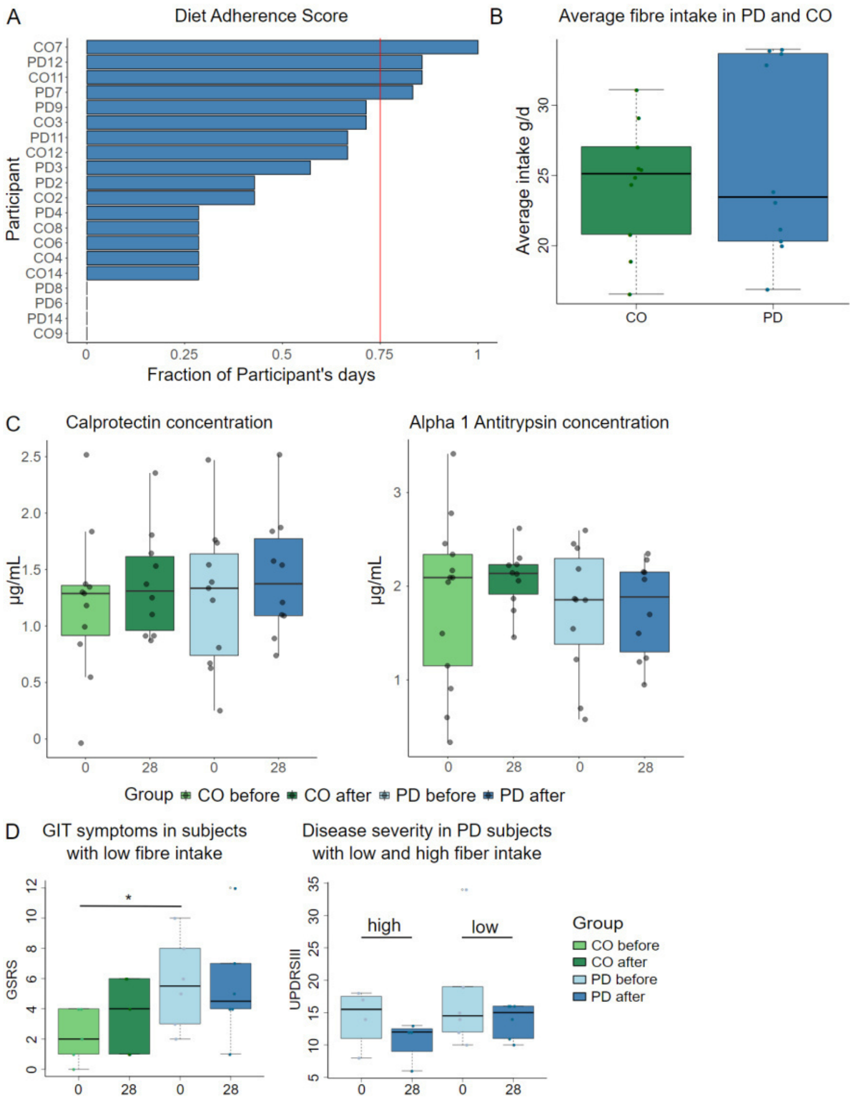

Supplementary Figure 1, dietary compliance and gut markers

A, the dietary compliance score based on the consumption of apples shows n=4 individuals, who were less adherent to the recommended diet (data is shown as the fraction of participants' diet days with an apple consumption below the mean apple consumption of all participants). A fraction of more than 0.75 days was considered as less adherent; B, average fibre intake was comparable in both study groups (Wilcoxon rank sum test, $p>0.05$), it should be noted that lactulose is in general not considered as fibre, because it is an artificial polysaccharide that exerts fibre-like prebiotic effects; C, gastrointestinal markers of intestinal inflammation (faecal calprotectin, left) or intestinal protein loss (faecal alpha-1-antitrypsin, right) were comparable between groups and did not change after prebiotics (Wilcoxon signed-rank test and Wilcoxon rank sum test, $p>0.05$); D, gastrointestinal symptoms stratified according to fibre intake showed that particularly PD individuals with low fibre intake during the study differed from CO before prebiotics and improved after prebiotics. Disease severity was less in PD patients with high fiber intake during the study (n.s.).

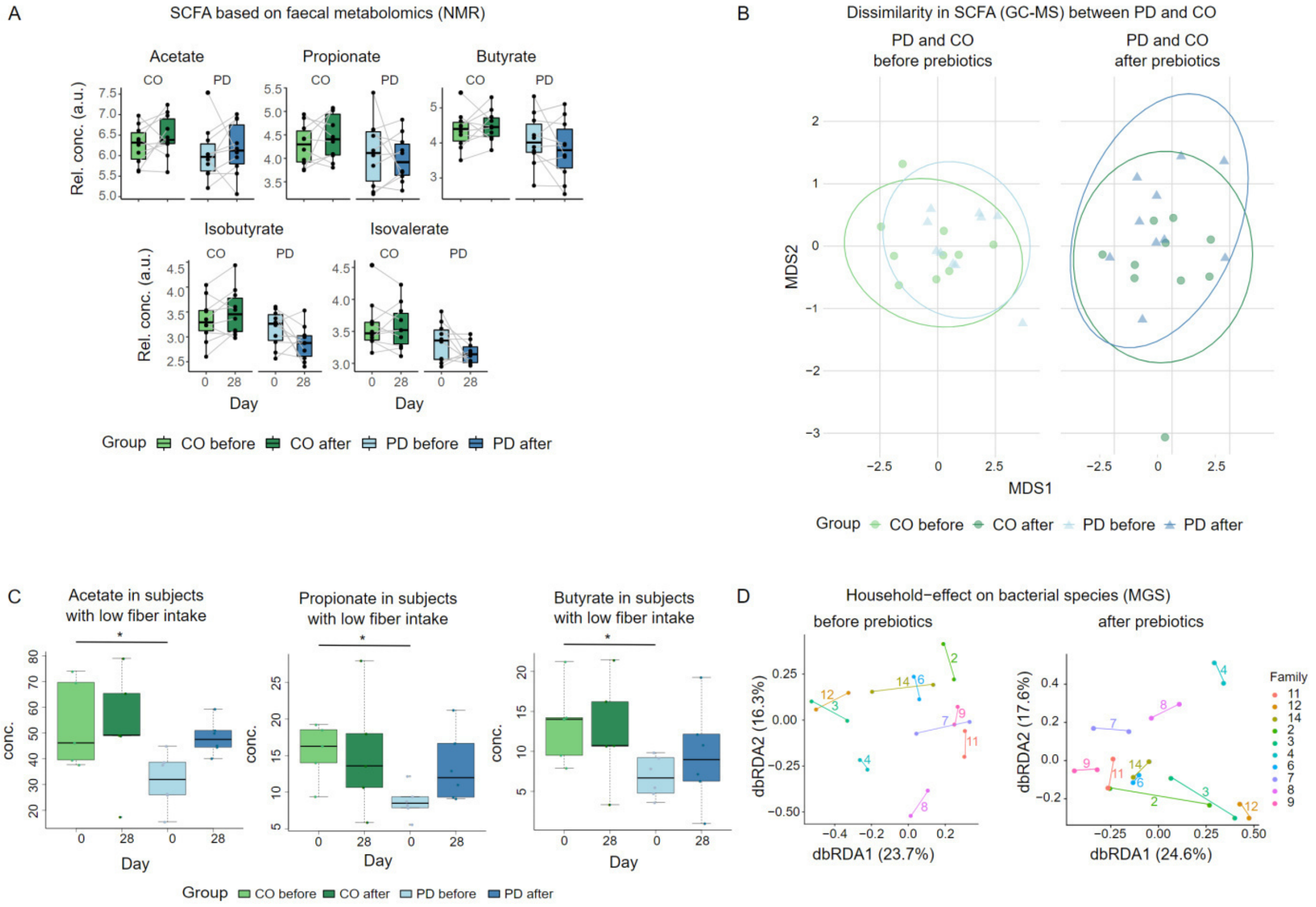

Supplementary Figure 2, SCFA and Household effect on bacterial species

A, SCFA rel. concentrations (a. u.) based on NMR metabolomics (log transformed data, a.u., arbitrary units) shows comparable baseline conditions between PD and CO similar to targeted SCFA measures. Rel. concentrations did not differ before vs. after prebiotics within each group; note, due to peak overlaps in NMR spectra, SCFA measures with NMR might differ from the targeted GC-MS measures displayed in Figure 1c, B, dissimilarity of SCFA composition (GC-MS, Gas Chromatography Mass Spectrometry) between PD and CO before and after prebiotics show that SCFA composition equalizes after prebiotics (perMANOVA, before R^2=0.13, p=0.08, after R^2=0.01, p=0.8, unconstrained dbRDA, Euclidean distance, targeted SCFA measures); C, SCFA (GC-MS) stratified for fiber intake showed, that several SCFA were markedly lower in PD than in CO before prebiotics in subjects with a low fiber intake during the study, which ameliorates after prebiotics; D, Household effect on bacterial species (MGS) after removing inter-individual effects was retained.

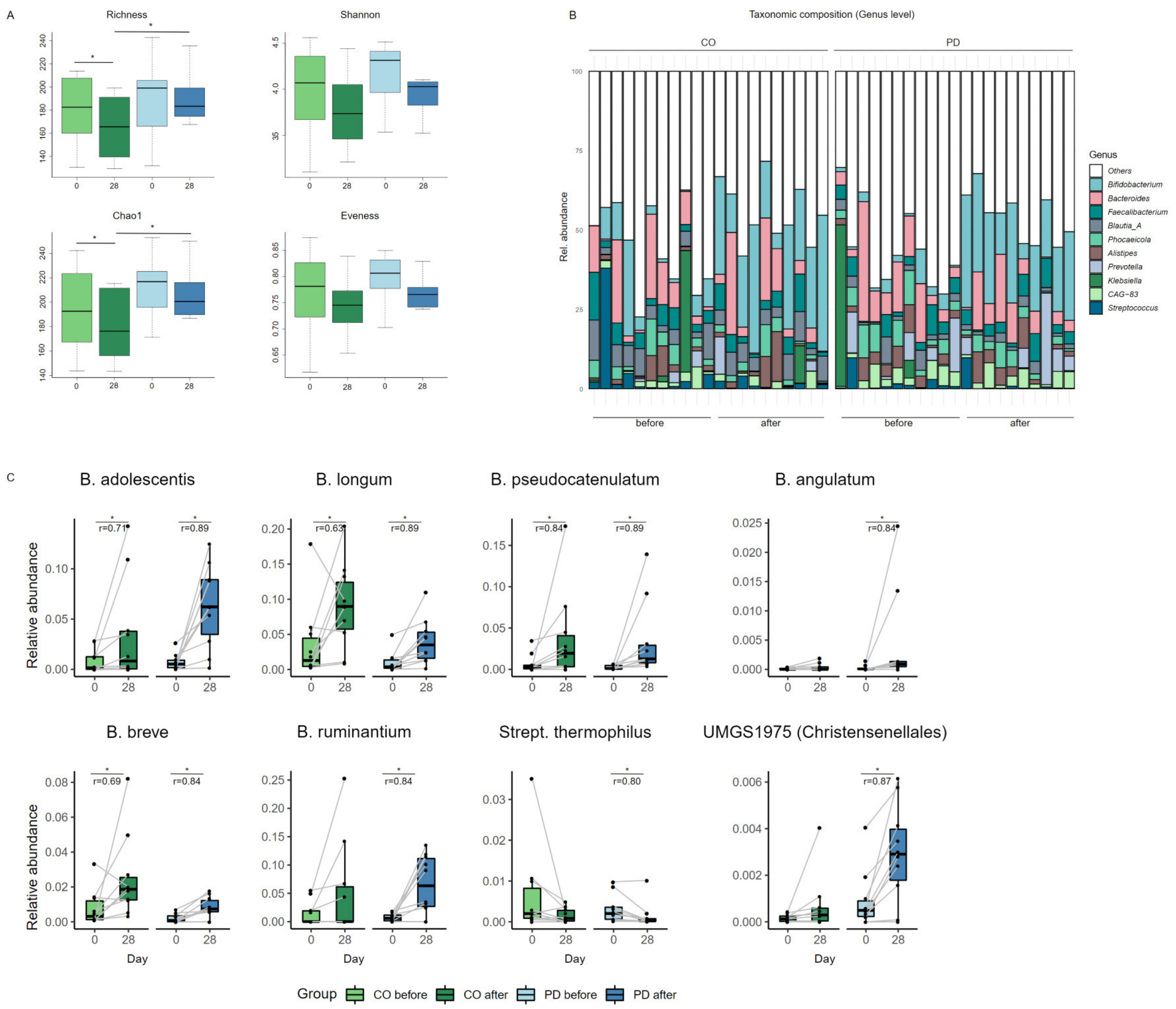

Supplementary Figure 3, Richness and bifidogenic effect of the prebiotic intervention

A, Species level alpha diversity, richness, and evenness was comparable between study groups before prebiotics, but richness decreased within CO and relative to PD after prebiotics (Wilcoxon signed-rank test, $p<0.05$); B, Composition of the top 10 Genera, separated for PD and CO; C, before vs. after comparisons (Wilcoxon signed-rank test revealed that several Bifidobacteria spp. increased after prebiotics, boxplots show relative abundance of taxa. n=6 *Bifidobacteria spp.* were enriched in PD after prebiotics, of which n=4 were also enriched in CO individuals; n=2 taxa were only sign. different in PD (Strept. Thermophilus, UMGS1975). * = $p<0.05$ (post-hoc test), r gives the respective effect size for post-hoc tests.

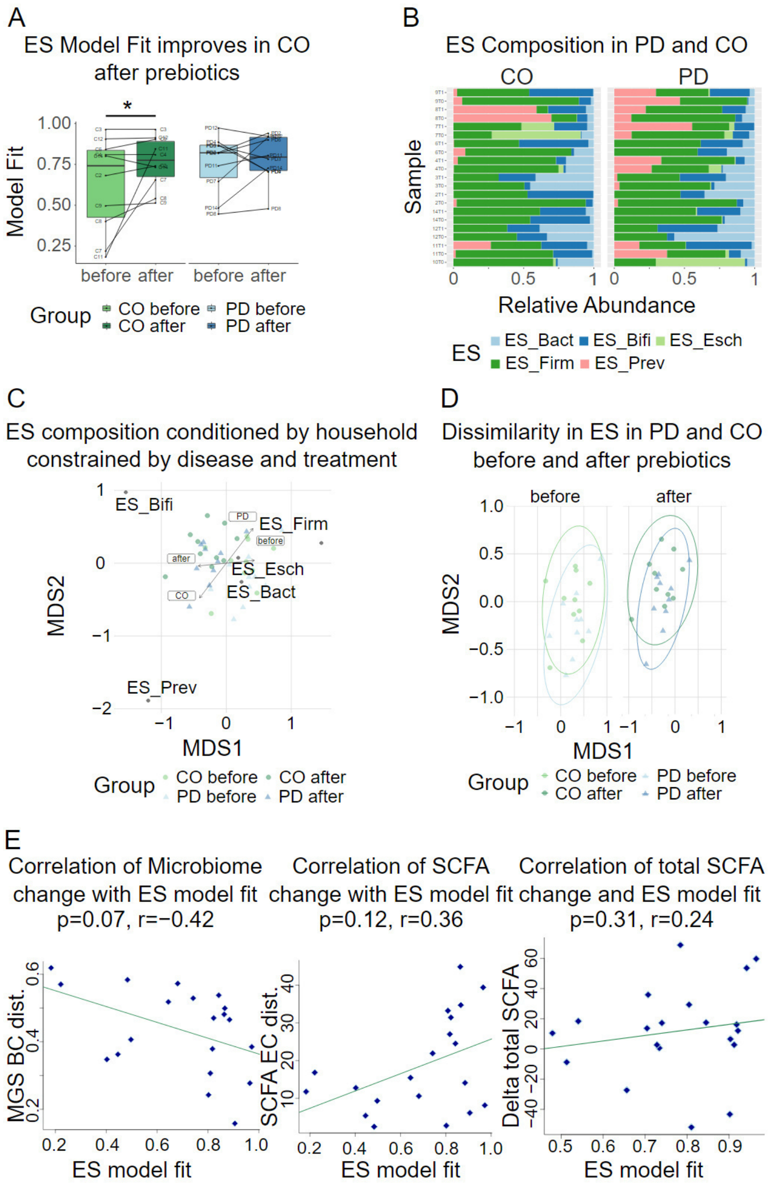

Supplementary Figure 4 Enterosignatures

A, Enterosignature (ES) model fit is comparable between groups and most samples have a model fit above 0.75. Note that only the CO group showed an increased model fit after prebiotics ($p=0.049$,Wilcoxon signed-rank test); B, ES composition along study samples shown as the relative abundance of the respective ES per patient; C, Dissimilarity in ES composition was explained by the dietary intervention ($R^2=0.30$, $p<0.001$, perMANOVA); D, Dissimilarity in ES composition showed no difference between PD and CO neither before nor after prebiotics (unconstrained dbRDA, Bray-Curtis distance). * = $p<0.05$; E, correlation of changes in microbiome based on MGS (difference in Bray Curtis distance, BC, before vs. after prebiotics) or SCFA (difference in Euclidean distance, EC, before vs. after prebiotics or change in total SCFA conc. per sample before vs. after prebiotics) correlated to the ES model fit at baseline as a measure of microbiome “normality”. r= correlation coefficient based on spearman correlation.

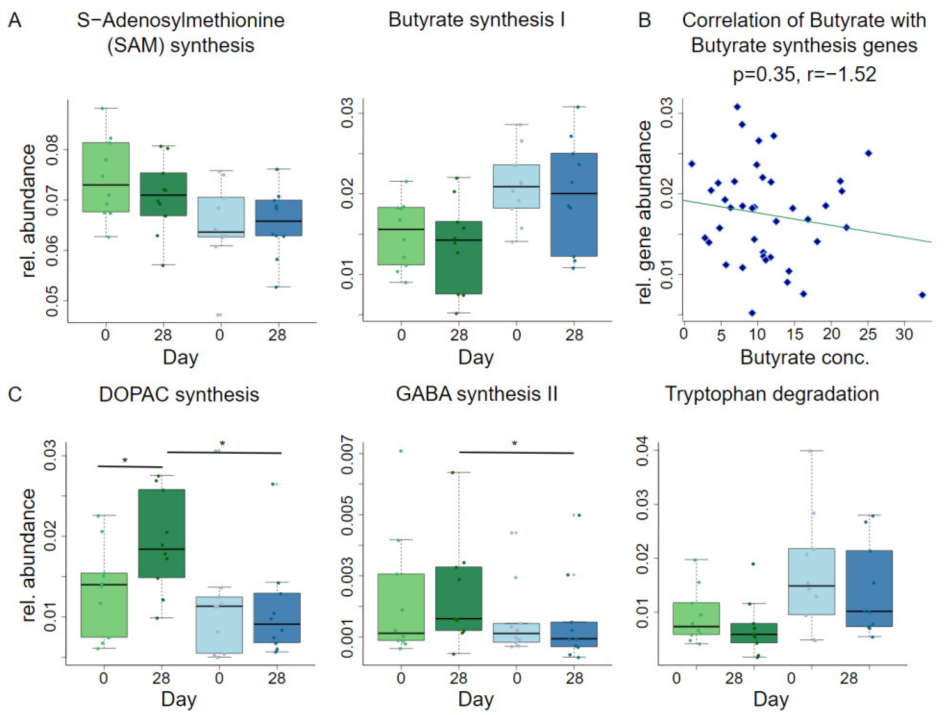

Supplementary Figure 5, Brain relevant functional metabolic potential

A, while a decrease in gene abundance for S-adenosylmethionine (SAM) synthesis in PD before ($p<0.05$, $q>0.1$, Wilcoxon signed-rank test) was ameliorated after prebiotics, a decreased gene abundance for Butyrate synthesis persisted in PD relative to CO during the study ($p<0.05$, $q>0.1$, Wilcoxon signed-rank test); B, a relevant correlation between Butyrate synthesis gene abundance and faecal Butyrate concentrations could not be observed. Transcriptomic data would be likely more informative; C, rel. gene abundance for DOPAC-synthesis, GABA-synthesis, and Trypotphan-degradation modules.

Supplementary Table 1

	PD	CO	p-value
Age (yrs)	64.73 ± 8.92	61.18 ± 7.92	p=0.31
Sex (f/m)	3/8	8/3	p=0.09
BMI (kg/m²)	26.81 ± 4.64	27.48 ± 4.71	p=0.72
Smoking [yes/past/never]	0/6/5	1/7/3	p=0.66
Levodopa [yes/no]	7/4	0/11	p=0.02
Dopamine-Agonist [yes/no]	10/1	0/11	P<0.0001
MAO-Inhibitor [yes/no]	1/10	0/11	p=0.48
COMT-Inhibitor [yes/no]	2/9	0/11	p=1
Statine intake [yes/no]	1/10	1/10	p=1
Diet<br>omnivor	 11	 11	p=n.a.
Probiotics at baseline<br>Yes<br>No<br>sometimes	 0<br>10<br>1	 2<br>9<br>0	p=1
Dairy consumption at baseline<br><1/d<br>2-3/d<br>NS	 6<br>4<br>1	 9<br>2<br>0	p=0.36
Vegetable consumption at baseline<br><1/d<br>2-3/d<br>3-4/d<br>5 or more/d<br>NS	 1<br>7<br>1<br>1<br>1	 0<br>9<br>2<br>0<br>0	p=0.59

Supplementary Table 1, Clinical characteristics and dietary baseline information
Dietary baseline information was acquired by a questionnaire assessing the dietary habits of the participants before the intervention; Note that one couple dropped out of the study after baseline measurements due to non-study related reasons; BMI, body mass index; PD, Parkinson's disease; CO, healthy control; Data is presented as mean ± standard deviation or absolute numbers of participants; NS, not specified. Differences between categorial data were tested with a Fisher's exact test.

Supplementary Table 2

	Data	Fiber intake during the study	
		High fiber (>25g/d)<br>N=9	Low fiber (<25g/d)<br>N=11
Difference between PD and CO before prebiotics	Acetate:	p= 0.9, q=1.0	**p= 0.03, q= 0.06***
	Propionate:	p=1.0, q=1.0	**p= 0.02, q= 0.06***
	Iso-Butyrate:	p=0.22, q=0.98	p= 0.50, q= 0.56
	Butyrate:	p=1.0, q=1.0	**p= 0.03, q= 0.06***
	Iso-Valerate:	p=0.33, q=0.98	p= 0.25, q= 0.37
	Valerate:	p=0.90, q=1.0	p= 0.79, q= 0.79
	GSRS:	p=0.17, q= 0.26	**p=0.07, q=0.07***
	Stool frequency:	p=0.46, q=0.46	**p=0.04, q=0.06***
Difference between PD and CO after prebiotics	Acetate:	p=0.73, q=0.90	p= 0.66, q= 0.79
	Propionate:	p=0.90, q=0.90	p= 0.79, q= 0.79
	Iso-Butyrate:	p=0.90, q=0.90	p= 0.54, q= 0.79
	Butyrate:	p=0.73, q=0.90	p= 0.66, q= 0.79
	Iso-Valerate:	p=0.81, q=0.90	p= 0.66, q= 0.79
	Valerate:	p=0.90, q=0.90	p= 0.18, q= 0.79
	GSRS:	p=0.53, q=0.53	p=0.51, q=0.51
	Stool frequency:	p=0.13, q=0.20	p=0.07, q=0.10

Supplementary Table 2, stratification according to fiber intake during the study
SCFA and clinical scores (GSRS, Stool frequency) differed between PD and CO at baseline related to the fiber intake during the study. After the intervention and in participants with higher fiber intake, no differences were observed; note, as baseline data of habitual fiber intake before the study were lacking, we stratified participants according to their fiber intake during the study.

Supplementary Table 3

**Data**	**R^2**	**p-value**
MGS Species	0.46	<0.001
Species	0.46	<0.001
Genus	0.47	<0.001
Family	0.44	<0.001
Order	0.46	<0.001
Class	0.41	<0.001
Phylum	0.41	<0.001
Functional metabolic potential (KEGG, general)	0.42	<0.001
Functional metabolic potential (KEGG/GBM, brain-relevant)	0.49	<0.001
Urine metabolomics	0.36	<0.001
Faeces metabolomics	0.45	<0.001

Supplementary Table 3, Household-effect on different data types assessed in this study

Supplementary Table 4

Taxonomic rank	Household effect before prebiotics	Household effect after prebiotics
MGS	R^2=0.56, p=0.001	R^2=0.56, p=0.004
Species	R^2= 0.56, p=0.002	R^2= 0.56, p=0.003
Genus	R^2=0.58, p= 0.001	R^2= 0.59 p= 0.004
Family	R^2=0.57, p=0.015	R^2=0.56, p=0.046

Supplementary Table 4, Household effect on different taxonomic ranks removing inter-individual effects

Supplementary Table 5

PDT0 vs. COT0	R^2=0.08, p=0.045
PDT0 vs. COT1	R^2=0.29, p=0.002
PDT1 vs. COT1	R^2=0.05, p=0.36
PDT1 vs. COT0	R^2=0.53, p= p=0.002
COT0 vs. COT1	R^2=0.18, p= 0.002
PDT0 vs. PDT1	R^2=0.23, p= 0.002

Supplementary Table 5, metabolome composition shifts
perMANOVA tests showed that the PD and CO metabolomes are most similar both before and after the diet intervention, but dissimilar when comparing pre to post intervention groups. This reflects the strong impact the diet intervention had and that both metabolomes moved in similar directions. Further, the change in the PD metabolome before vs. after was greater in the PD group, i.e. the CO group was more robust in their metabolomic composition.

Supplementary Table 6, KEGG and GBM Modules univariate testing

**KEGG PD vs. CO comparisons**				
**no differences**				

**KEGG Before vs. after comparisons, mean relative pathway abundance**									
**KEGG Modules with changes over time shared in PD and CO, mean relative pathway abundance**									
**Module**	**X**	**X.1**	**X.2**	**X.3**	**COT0**	**COT1**	**PDT0**	**PDT1**	
M00021	Pathway module	Nucleotide and amino acid metabolism	Cysteine and methionine metabolism	Cysteine biosynthesis, serine => cysteine	0.0063	0.0054	0.0063	0.0054	decrease
M00345	Pathway module	Energy metabolism	Methane metabolism	Formaldehyde assimilation, ribulose	0.0086	0.0077	0.0085	0.0076	

				monophosphate pathway					
M00618	Signature module	Gene set	Metabolic capacity	Acetogen	0.0041	0.0039	0.0042	0.0038	
M00299	Structural complex	Environmental information processing	Mineral and organic ion transport system	Spermidine/putrescine transport system	0.0053	0.0045	0.0052	0.0044	
M00307	Pathway module	Carbohydrate and lipid metabolism	Central carbohydrate metabolism	Pyruvate oxidation, pyruvate => acetyl-CoA	0.0274	0.0240	0.0279	0.0243	
M00052	Pathway module	Nucleotide and amino acid metabolism	Pyrimidine metabolism	Pyrimidine ribonucleotide biosynthesis, UMP => UDP/UTP,CDP/CTP	0.0078	0.0069	0.0077	0.0068	
M00742	Functional set	Environmental information processing	Drug resistance	Aminoglycoside resistance, protease FtsH	0.0153	0.0143	0.0148	0.0135	
M00121	Pathway module	Nucleotide and amino acid metabolism	Cofactor and vitamin biosynthesis	Heme biosynthesis, glutamate => protoheme/siroheme	0.0050	0.0040	0.0046	0.0037	
M00122	Pathway module	Nucleotide and amino acid metabolism	Cofactor and vitamin biosynthesis	Cobalamin biosynthesis, cobinamide => cobalamin	0.0089	0.0078	0.0091	0.0077	
M00006	Pathway module	Carbohydrate and lipid metabolism	Central carbohydrate metabolism	Pentose phosphate pathway, oxidative phase, glucose 6P => ribulose 5P	0.0016	0.0026	0.0017	0.0026	increase
M00207	Structural complex	Environmental information processing	Saccharide, polyol, and lipid transport system	Putative multiple sugar transport system	0.0108	0.0124	0.0099	0.0121	
M00196	Structural complex	Environmental information processing	Saccharide, polyol, and lipid transport system	Raffinose/stachyose/melibiose transport system	0.0068	0.0093	0.0059	0.0086	
M00394	Structural complex	Genetic information processing	RNA processing	RNA degradosome	0.0056	0.0072	0.0055	0.0072	
M00083	Pathway module	Carbohydrate and lipid metabolism	Fatty acid metabolism	Fatty acid biosynthesis, elongation	0.0034	0.0127	0.0026	0.0129	
M00082	Pathway module	Carbohydrate and lipid metabolism	Fatty acid metabolism	Fatty acid biosynthesis, initiation	0.0038	0.0082	0.0033	0.0082	
M00126	Pathway module	Nucleotide and amino	Cofactor and vitamin biosynthesis	Tetrahydrofolate biosynthesis, GTP => THF	0.0028	0.0035	0.0028	0.0035	

		acid metabolism							
M00051	Pathway module	Nucleotide and amino acid metabolism	Pyrimidine metabolism	Uridine monophosphate biosynthesis, glutamine (+ PRPP) => UMP	0.0055	0.0061	0.0055	0.0065	
M00086	Pathway module	Carbohydrate and lipid metabolism	Fatty acid metabolism	beta-Oxidation, acyl-CoA synthesis	0.0181	0.0236	0.0193	0.0245	
M00443	Functional set	Environmental information processing	Two-component regulatory system	SenX3-RegX3 (phosphate starvation response) two-component regulatory system	0.0006	0.0018	0.0005	0.0018	
**KEGG Modules with changes over time uniquely in PD, mean relative pathway abundance**									
**Module**	**X**	**X.1**	**X.2**	**X.3**	**PDT0**	**PDT1**			
M00492	Functional set	Environmental information processing	Two-component regulatory system	LytS-LytR two-component regulatory system	0.0011	0.0009			decrease
M00627	Signature module	Gene set	Drug resistance	beta-Lactam resistance, Bla system	0.0028	0.0022			
M00434	Functional set	Environmental information processing	Two-component regulatory system	PhoR-PhoB (phosphate starvation response) two-component regulatory system	0.0109	0.0093			
M00159	Structural complex	Energy metabolism	ATP synthesis	V/A-type ATPase, prokaryotes	0.0022	0.0018			
M00001	Pathway module	Carbohydrate and lipid metabolism	Central carbohydrate metabolism	Glycolysis (Embden-Meyerhof pathway), glucose => pyruvate	0.0104	0.0096			
M00616	Signature module	Gene set	Metabolic capacity	Sulfate-sulfur assimilation	0.0012	0.0008			
M00308	Pathway module	Carbohydrate and lipid metabolism	Central carbohydrate metabolism	Semi-phosphorylative Entner-Doudoroff pathway, gluconate => glycerate-3P	0.003	0.0026			
M00240	Structural complex	Environmental information processing	Metallic cation, iron-siderophore and vitamin	Iron complex transport system	0.0105	0.0093			

			B12 transport system						
M00577	Pathway module	Nucleotide and amino acid metabolism	Cofactor and vitamin biosynthesis	Biotin biosynthesis, BioW pathway, pimelate => pimeloyl-CoA => biotin	0.0026	0.002			
M00454	Functional set	Environmental information processing	Two-component regulatory system	KdpD-KdpE (potassium transport) two-component regulatory system	0.0058	0.0045			
M00236	Structural complex	Environmental information processing	Phosphate and amino acid transport system	Putative polar amino acid transport system	0.0077	0.0091			increase
M00565	Pathway module	Carbohydrate and lipid metabolism	Sugar metabolism	Trehalose biosynthesis, D-glucose 1P => trehalose	0.0209	0.024			
M00237	Structural complex	Environmental information processing	Phosphate and amino acid transport system	Branched-chain amino acid transport system	0.0046	0.0062			
M00020	Pathway module	Nucleotide and amino acid metabolism	Serine and threonine metabolism	Serine biosynthesis, glycerate-3P => serine	0.0056	0.0068			
M00256	Structural complex	Environmental information processing	ABC-2 type and other transport systems	Cell division transport system	0.0047	0.0055			
M00260	Structural complex	Genetic information processing	DNA polymerase	DNA polymerase III complex, bacteria	0.0105	0.0117			
M00005	Pathway module	Carbohydrate and lipid metabolism	Central carbohydrate metabolism	PRPP biosynthesis, ribose 5P => PRPP	0.008	0.0094			
M00118	Pathway module	Nucleotide and amino acid metabolism	Cofactor and vitamin biosynthesis	Glutathione biosynthesis, glutamate => glutathione	0.0008	0.0015			
M00239	Structural complex	Environmental information processing	Peptide and nickel transport system	Peptides/nickel transport system	0.0069	0.0088			
M00018	Pathway module	Nucleotide and amino acid metabolism	Serine and threonine metabolism	Threonine biosynthesis, aspartate => homoserine => threonine	0.0058	0.0067			
**KEGG Modules with changes over time uniquely in CO, mean relative pathway abundance**									

KEGG Module	X.1	X.2	X.3		COT0	COT1			
M00254	Structural complex	Environmental information processing	ABC-2 type and other transport systems	ABC-2 type transport system	0.0368	0.0348			decrease
M00364	Pathway module	Carbohydrate and lipid metabolism	Terpenoid backbone biosynthesis	C10-C20 isoprenoid biosynthesis, bacteria	0.0046	0.0041			
M00022	Pathway module	Nucleotide and amino acid metabolism	Aromatic amino acid metabolism	Shikimate pathway, phosphoenolpyruvate + erythrose-4P => chorismate	0.0063	0.0059			
M00089	Pathway module	Carbohydrate and lipid metabolism	Lipid metabolism	Triacylglycerol biosynthesis	0.0051	0.0045			
M00026	Pathway module	Nucleotide and amino acid metabolism	Histidine metabolism	Histidine biosynthesis, PRPP => histidine	0.0037	0.0033			
M00190	Structural complex	Environmental information processing	Mineral and organic ion transport system	Iron(III) transport system	0.0024	0.0017			
M00216	Structural complex	Environmental information processing	Saccharide, polyol, and lipid transport system	Multiple sugar transport system	0.0012	0.0017			increase
Data is presented as mean values of the relative pathway abundance									

KEGG Before vs. after comparisons, Effect sizes								
**Module**	**X**	**X.1**	**X.2**	**X.3**	**PDpvals**	**COpvals**	**effPD**	**effCO**
M00254	Structural complex	Environmental information processing	ABC-2 type and other transport systems	ABC-2 type transport system	0,105	0,037	0,532	0,661
M00364	Pathway module	Carbohydrate and lipid metabolism	Terpenoid backbone biosynthesis	C10-C20 isoprenoid biosynthesis, bacteria	0,557	0,027	0,210	0,693
M00236	Structural complex	Environmental information processing	Phosphate and amino acid transport system	Putative polar amino acid transport system	0,049	0,432	0,629	0,274
M00492	Functional set	Environmental information processing	Two-component regulatory system	LytS-LytR two-component regulatory system	0,010	0,322	0,790	0,338

M00627	Signature module	Gene set	Drug resistance	beta-Lactam resistance, Bla system	0,014	0,625	0,757	0,177
M00565	Pathway module	Carbohydrate and lipid metabolism	Sugar metabolism	Trehalose biosynthesis, D-glucose 1P => trehalose	0,037	0,160	0,661	0,467
M00173	Pathway module	Energy metabolism	Carbon fixation Signature	Reductive citrate cycle (Arnon-Buchanan cycle)	0,084	0,193	0,564	0,435
M00434	Functional set	Environmental information processing	Two-component regulatory system	PhoR-PhoB (phosphate starvation response) two-component regulatory system	0,037	0,131	0,661	0,500
M00021	Pathway module	Nucleotide and amino acid metabolism	Cysteine and methionine metabolism	Cysteine biosynthesis, serine => cysteine	0,004	0,004	0,854	0,854
M00006	Pathway module	Carbohydrate and lipid metabolism	Central carbohydrate metabolism	Pentose phosphate pathway, oxidative phase, glucose 6P => ribulose 5P	0,037	0,006	0,661	0,822
M00579	Pathway module	Energy metabolism	Carbon fixation Signature	Phosphate acetyltransferase-acetate kinase pathway, acetyl-CoA => acetate	0,131	0,084	0,500	0,564
M00237	Structural complex	Environmental information processing	Phosphate and amino acid transport system	Branched-chain amino acid transport system	0,010	0,557	0,790	0,210
M00207	Structural complex	Environmental information processing	Saccharide, polyol, and lipid transport system	Putative multiple sugar transport system	0,014	0,049	0,757	0,629
M00022	Pathway module	Nucleotide and amino acid metabolism	Aromatic amino acid metabolism	Shikimate pathway, phosphoenolpyruvate + erythrose-4P => chorismate	0,375	0,049	0,306	0,629
M00119	Pathway module	Nucleotide and amino acid metabolism	Cofactor and vitamin biosynthesis	Pantothenate biosynthesis, valine/L-aspartate => pantothenate	0,064	0,064	0,596	0,596
M00345	Pathway module	Energy metabolism	Methane metabolism	Formaldehyde assimilation, ribulose monophosphate pathway	0,006	0,027	0,822	0,693
M00009	Pathway module	Carbohydrate and lipid metabolism	Central carbohydrate metabolism	Citrate cycle (TCA cycle, Krebs cycle)	0,084	0,275	0,564	0,371

M00089	Pathway module	Carbohydrate and lipid metabolism	Lipid metabolism	Triacylglycerol biosynthesis	0,275	0,002	0,371	0,886
M00189	Structural complex	Environmental information processing	Mineral and organic ion transport system	Molybdate transport system	0,084	0,160	0,564	0,467
M00212	Structural complex	Environmental information processing	Saccharide, polyol, and lipid transport system	Ribose transport system	0,105	0,064	0,532	0,596
M00020	Pathway module	Nucleotide and amino acid metabolism	Serine and threonine metabolism	Serine biosynthesis, glycerate-3P => serine	0,002	0,322	0,886	0,338
M00256	Structural complex	Environmental information processing	ABC-2 type and other transport systems	Cell division transport system	0,002	0,084	0,886	0,564
M00196	Structural complex	Environmental information processing	Saccharide, polyol, and lipid transport system	Raffinose/stachyose/melibiose transport system	0,014	0,027	0,757	0,693
M00394	Structural complex	Genetic information processing	RNA processing	RNA degradosome	0,002	0,014	0,886	0,757
M00076	Pathway module	Carbohydrate and lipid metabolism	Glycosaminoglycan metabolism	Dermatan sulfate degradation	0,064	0,375	0,596	0,306
M00618	Signature module	Gene set	Metabolic capacity	Acetogen	0,049	0,049	0,629	0,629
M00250	Structural complex	Environmental information processing	ABC-2 type and other transport systems	Lipopolysaccharide transport system	0,131	0,084	0,500	0,564
M00299	Structural complex	Environmental information processing	Mineral and organic ion transport system	Spermidine/putrescine transport system	0,037	0,037	0,661	0,661
M00260	Structural complex	Genetic information processing	DNA polymerase	DNA polymerase III complex, bacteria	0,014	0,084	0,757	0,564
M00083	Pathway module	Carbohydrate and lipid metabolism	Fatty acid metabolism	Fatty acid biosynthesis, elongation	0,002	0,010	0,886	0,790
M00082	Pathway module	Carbohydrate and lipid metabolism	Fatty acid metabolism	Fatty acid biosynthesis, initiation	0,004	0,020	0,854	0,725
M00307	Pathway module	Carbohydrate and lipid metabolism	Central carbohydrate metabolism	Pyruvate oxidation, pyruvate => acetyl-CoA	0,010	0,020	0,790	0,725
M00026	Pathway module	Nucleotide and amino acid metabolism	Histidine metabolism	Histidine biosynthesis, PRPP => histidine	0,322	0,006	0,338	0,822

M00159	Structural complex	Energy metabolism	ATP synthesis	V/A-type ATPase, prokaryotes	0,037	0,322	0,661	0,338
M00004	Pathway module	Carbohydrate and lipid metabolism	Central carbohydrate metabolism	Pentose phosphate pathway (Pentose phosphate cycle)	0,131	0,105	0,500	0,532
M00001	Pathway module	Carbohydrate and lipid metabolism	Central carbohydrate metabolism	Glycolysis (Embden-Meyerhof pathway), glucose => pyruvate	0,014	0,432	0,757	0,274
M00247	Structural complex	Environmental information processing	Metallic cation, iron-siderophore and vitamin B12 transport system	Putative ABC transport system	0,084	0,131	0,564	0,500
M00729	Functional set	Environmental information processing	Drug resistance	Fluoroquinolone resistance, gyrase-protecting protein Qnr	0,105	0,131	0,532	0,500
M00126	Pathway module	Nucleotide and amino acid metabolism	Cofactor and vitamin biosynthesis	Tetrahydrofolate biosynthesis, GTP => THF	0,020	0,004	0,725	0,854
M00616	Signature module	Gene set	Metabolic capacity	Sulfate-sulfur assimilation	0,014	0,232	0,757	0,403
M00308	Pathway module	Carbohydrate and lipid metabolism	Central carbohydrate metabolism	Semi-phosphorylative Entner-Doudoroff pathway, gluconate => glycerate-3P	0,020	0,232	0,725	0,403
M00373	Pathway module	Carbohydrate and lipid metabolism	Other carbohydrate metabolism	Ethylmalonyl pathway	0,084	0,084	0,564	0,564
M00532	Pathway module	Carbohydrate and lipid metabolism	Other carbohydrate metabolism	Photorespiration	0,064	0,232	0,596	0,403
M00052	Pathway module	Nucleotide and amino acid metabolism	Pyrimidine metabolism	Pyrimidine ribonucleotide biosynthesis, UMP => UDP/UTP,CDP/CTP	0,049	0,027	0,629	0,693
M00190	Structural complex	Environmental information processing	Mineral and organic ion transport system	Iron(III) transport system	0,131	0,020	0,500	0,725
M00240	Structural complex	Environmental information processing	Metallic cation, iron-siderophore and vitamin	Iron complex transport system	0,027	0,105	0,693	0,532

			B12 transport system					
M00216	Structural complex	Environmental information processing	Saccharide, polyol, and lipid transport system	Multiple sugar transport system	0,160	0,037	0,467	0,661
M00742	Functional set	Environmental information processing	Drug resistance	Aminoglycoside resistance, protease FtsH	0,020	0,027	0,725	0,693
M00005	Pathway module	Carbohydrate and lipid metabolism	Central carbohydrate metabolism	PRPP biosynthesis, ribose 5P => PRPP	0,002	0,557	0,886	0,210
M00007	Pathway module	Carbohydrate and lipid metabolism	Central carbohydrate metabolism	Pentose phosphate pathway, non-oxidative phase, fructose 6P => ribose 5P	0,131	0,084	0,500	0,564
M00051	Pathway module	Nucleotide and amino acid metabolism	Pyrimidine metabolism	Uridine monophosphate biosynthesis, glutamine (+ PRPP) => UMP	0,002	0,049	0,886	0,629
M00121	Pathway module	Nucleotide and amino acid metabolism	Cofactor and vitamin biosynthesis	Heme biosynthesis, glutamate => protoheme/siroheme	0,037	0,006	0,661	0,822
M00577	Pathway module	Nucleotide and amino acid metabolism	Cofactor and vitamin biosynthesis	Biotin biosynthesis, BioW pathway, pimelate => pimeloyl-CoA => biotin	0,037	0,432	0,661	0,274
M00086	Pathway module	Carbohydrate and lipid metabolism	Fatty acid metabolism	beta-Oxidation, acyl-CoA synthesis	0,010	0,002	0,790	0,886
M00118	Pathway module	Nucleotide and amino acid metabolism	Cofactor and vitamin biosynthesis	Glutathione biosynthesis, glutamate => glutathione	0,004	0,064	0,854	0,596
M00454	Functional set	Environmental information processing	Two-component regulatory system	KdpD-KdpE (potassium transport) two-component regulatory system	0,037	0,084	0,661	0,564
M00614	Signature module	Gene set	Metabolic capacity	Anoxygenic photosynthesis in green sulfur bacteria	0,084	0,193	0,564	0,435
M00443	Functional set	Environmental information processing	Two-component regulatory system	SenX3-RegX3 (phosphate starvation response) two-component	0,002	0,010	0,886	0,790

				regulatory system				
M00239	Structural complex	Environmental information processing	Peptide and nickel transport system	Peptides/nickel transport system	0,037	0,193	0,661	0,435
M00018	Pathway module	Nucleotide and amino acid metabolism	Serine and threonine metabolism	Threonine biosynthesis, aspartate => homoserine => threonine	0,027	0,432	0,693	0,274
M00122	Pathway module	Nucleotide and amino acid metabolism	Cofactor and vitamin biosynthesis	Cobalamin biosynthesis, cobinamide => cobalamin	0,002	0,027	0,886	0,693

**GBM PD vs. CO comparisons**				
**GBM**	**prepvals**	**preqvals**	**postpvals**	**postqvals**
Kynurenine synthesis	0,770	0,932	0,846	0,865
Tryptophan synthesis	0,432	0,882	0,432	0,559
Glutamate synthesis I	0,557	0,932	0,275	0,505
Glutamate synthesis II	0,193	0,594	0,432	0,559
Histamine synthesis	0,695	0,932	0,375	0,550
Histamine degradation	0,590	0,932	0,590	0,701
p-Cresol synthesis	1,000	1,000	0,275	0,505
p-Cresol degradation	0,076	0,451	0,080	0,370
Kynurenine degradation	NA	NA	0,371	0,550
GABA degradation	0,846	0,932	0,084	0,370
GABA synthesis I	0,353	0,842	0,234	0,490
GABA synthesis II	0,625	0,932	0,002	0,029
GABA synthesis III	0,232	0,625	0,193	0,448
Dopamine degradation	0,906	0,974	0,722	0,815
DOPAC synthesis	0,232	0,625	0,002	0,029
Nitric oxide synthesis II (nitrite reductase)	0,813	0,932	0,722	0,815
Nitric oxide degradation I (NO dioxygenase)	0,625	0,932	0,375	0,550
Nitric oxide degradation II (NO reductase)	0,787	0,932	0,178	0,448
ClpB (ATP-dependent chaperone protein)	0,846	0,932	0,492	0,619
17-beta-Estradiol degradation	0,193	0,594	0,002	0,029
Quinolinic acid synthesis	0,492	0,882	0,131	0,443
Quinolinic acid degradation	1,000	1,000	0,193	0,448
Isovaleric acid synthesis I (KADH pathway)	0,492	0,882	0,770	0,846
Isovaleric acid synthesis II (KADC pathway)	0,064	0,451	0,010	0,107
S-Adenosylmethionine (SAM) synthesis	0,014	0,294	0,275	0,505
Inositol synthesis	0,193	0,594	0,105	0,422
Inositol degradation	0,322	0,815	0,922	0,922
g-Hydroxybutyric acid (GHB) degradation	0,846	0,932	0,160	0,448
Menaquinone synthesis (vitamin K2) I	0,375	0,849	0,432	0,559
Menaquinone synthesis (vitamin K2) II (alternative pathway: futalosine pathway)	0,492	0,882	0,375	0,550

Acetate synthesis I	0,846	0,932	0,232	0,490
Acetate synthesis II	0,695	0,932	0,160	0,448
Acetate synthesis III	0,846	0,932	0,193	0,448
Acetate synthesis IV	1,000	1,000	0,789	0,847
Acetate degradation	0,105	0,504	0,084	0,370
Propionate synthesis I	0,722	0,932	0,846	0,865
Tryptophan degradation	0,084	0,451	0,027	0,201
Glutamate degradation I	0,037	0,378	0,375	0,550
Glutamate degradation II	0,160	0,594	0,131	0,443
Butyrate synthesis I	0,037	0,378	0,020	0,172
Butyrate synthesis II	0,432	0,882	0,557	0,680
Propionate synthesis II	0,193	0,594	0,064	0,370
Propionate synthesis III	0,006	0,252	0,375	0,550
Propionate degradation I	0,044	0,378	0,407	0,559

**GBM Before vs. after comparisons mean relative pathway abundance**					
**GBM Modules with changes over time shared in PD and CO, mean relative pathway abundance**					
**Module**	**X**	**X.1**	**COT0**	**COT1**	
MGB022	GABA synthesis	GABA synthesis III	0,010	0,015	increase
MGB029	ClpB	ClpB (ATP-dependent chaperone protein)	0,120	0,133	
**GBM Modules with changes over time uniquely in PD, mean relative pathway abundance**					
**Module**	**X.1**	**X.2**	**PDT0**	**PDT1**	
MGB015	p-Cresol synthesis	p-Cresol synthesis	0,044	0,036	decrease
MGB038	Inositol degradation	Inositol degradation	0,011	0,010	
MGB044	Acetate synthesis	Acetate synthesis II	0,093	0,086	
MGB045	Acetate synthesis	Acetate synthesis III	0,096	0,089	
MGB006	Glutamate synthesis	Glutamate synthesis I	0,072	0,081	increase
MGB033	Quinolinic acid degradation	Quinolinic acid degradation	0,035	0,039	
MGB037	Inositol synthesis	Inositol synthesis	0,013	0,018	
**GBM Modules with changes over time uniquely in CO, mean relative pathway abundance**					
X	X.1	X.2	COT0	**COT1**	
MGB043	Acetate synthesis	Acetate synthesis I	0,049	0,044	decrease
MGB024	DOPAC synthesis	DOPAC synthesis	0,013	0,019	increase
Data is presented as mean values of the relative pathway abundance					

GBM Before vs. after comparisons, Effect sizes						
Module	X	**X.1**	**PDpvals**	**COpvals**	**effPD**	**effCO**
MGB006	Glutamate synthesis	Glutamate synthesis I	0,049	0,105	0,629	0,532
MGB015	p-Cresol synthesis	p-Cresol synthesis	0,006	0,131	0,822	0,500
MGB022	GABA synthesis	GABA synthesis III	0,037	0,037	0,661	0,661
MGB024	DOPAC synthesis	DOPAC synthesis	1,000	0,004	0,016	0,854
MGB029	ClpB	ClpB (ATP-dependent chaperone protein)	0,002	0,010	0,886	0,790
MGB033	Quinolinic acid degradation	Quinolinic acid degradation	0,014	0,922	0,757	0,048
MGB037	Inositol synthesis	Inositol synthesis	0,027	0,064	0,693	0,596
MGB038	Inositol degradation	Inositol degradation	0,049	0,084	0,629	0,564
MGB043	Acetate synthesis	Acetate synthesis I	0,064	0,027	0,596	0,693
MGB044	Acetate synthesis	Acetate synthesis II	0,020	0,322	0,725	0,338
MGB045	Acetate synthesis	Acetate synthesis III	0,020	0,275	0,725	0,371
MGB047	Acetate degradation	Acetate degradation	0,131	0,232	0,500	0,403
MGB049	Tryptophan degradation	Tryptophan degradation	0,084	0,322	0,564	0,338
MGB054	Propionate synthesis	Propionate synthesis II	0,084	0,105	0,564	0,532

Supplementary Table 7, correlation analyses

Correlations in PD and CO KEGG Modules				
**MGS**	**Pathway/KEGG Module**	**p-Value**	**q-Value**	**corr-coeff**
Bacteria_Actinobacteriota_Actinomycetia_Actinomycetales_Bifidobacteriaceae_Bifidobacterium_Bifidobacterium longum_MB2bin141	Pathway module_ Nucleotide and amino acid metabolism_ Cysteine and methionine metabolism_Cysteine biosynthesis, serine => cysteine	0,000	0,012	-0,594
Bacteria_Bacteroidota_Bacteroidia_Bacteroidales_Rikenellaceae_Alistipes_Alistipes putredinis_MB2bin34	Pathway module_ Nucleotide and amino acid metabolism_ Cysteine and methionine metabolism_Cysteine biosynthesis, serine => cysteine	0,001	0,080	0,494
Bacteria_Actinobacteriota_Actinobacteria_Actinomycetales_Bifidobacteriaceae_Bifidobacterium_Bifidobacterium breve_specI_v3_Cluster1098	Pathway module_ Nucleotide and amino acid metabolism_ Cysteine and methionine metabolism_Cysteine biosynthesis, serine => cysteine	0,000	0,025	-0,550
Bacteria_Actinobacteriota_Actinomycetia_Actinomycetales_Bifidobacteriaceae_Bifidobacterium_Bifidobacterium longum_MB2bin141	Pathway module_ Carbohydrate and lipid metabolism_ Central carbohydrate metabolism_Pentose phosphate pathway, oxidative phase, glucose 6P => ribulose 5P	0,000	0,041	0,556

Bacteria_Actinobacteriota_Actinomycetia_Actinomycetales_Bifidobacteriaceae_Bifidobacterium_Bifidobacterium adolescentis_MB2bin135	Structural complex_ Environmental information processing_ Saccharide, polyol, and lipid transport system_Putative multiple sugar transport system	0,000	0,021	0,577
Bacteria_Firmicutes_A_Clostridia_A_Christensenellales_QAND01_UMGS1975_UMGS1975 sp900546685_MB2bin272	Structural complex_ Environmental information processing_ Saccharide, polyol, and lipid transport system_Putative multiple sugar transport system	0,000	0,021	0,542
Bacteria_Bacteroidota_Bacteroidia_Bacteroidales_Rikenellaceae_Alistipes_Alistipes obesi_MB2bin28	Structural complex_ Environmental information processing_ Saccharide, polyol, and lipid transport system_Putative multiple sugar transport system	0,001	0,025	-0,527
Bacteria_Actinobacteriota_Actinomycetia_Actinomycetales_Bifidobacteriaceae_Bifidobacterium_Bifidobacterium angulatum_MB2bin288	Structural complex_ Environmental information processing_ Saccharide, polyol, and lipid transport system_Putative multiple sugar transport system	0,002	0,064	0,475
Bacteria_Bacteroidota_Bacteroidia_Bacteroidales_Rikenellaceae_Alistipes_Alistipes finegoldii_MB2bin46	Structural complex_ Environmental information processing_ Saccharide, polyol, and lipid transport system_Putative multiple sugar transport system	0,001	0,032	-0,509
Bacteria_Actinobacteriota_Actinobacteria_Actinomycetales_Bifidobacteriaceae_Bifidobacterium_Bifidobacterium ruminantium_specI_v3_Cluster2702	Structural complex_ Environmental information processing_ Saccharide, polyol, and lipid transport system_Putative multiple sugar transport system	0,000	0,021	0,543
Bacteria_Bacteroidota_Bacteroidia_Bacteroidales_Rikenellaceae_Alistipes_Alistipes shahii_MB2bin10	Structural complex_ Environmental information processing_ Saccharide, polyol, and lipid transport system_Raffinose/stachyose/melibiose transport system	0,003	0,043	-0,460
Bacteria_Bacteroidota_Bacteroidia_Bacteroidales_Bacteroidaceae_Bacteroides_Bacteroides ovatus_MB2bin110	Structural complex_ Environmental information processing_ Saccharide, polyol, and lipid transport system_Raffinose/stachyose/melibiose transport system	0,002	0,035	-0,480
Bacteria_Bacteroidota_Bacteroidia_Bacteroidales_Barnesiellaceae_Barnesiella_Barnesiella intestinihominis_MB2bin112	Structural complex_ Environmental information processing_ Saccharide, polyol, and lipid transport	0,005	0,050	-0,445

	system_Raffinose/stachyose/melibiose transport system			
Bacteria_Firmicutes_A_Clostridia_Lachnospirales_Lachnospiraceae_Anaerobutyricum_?_MB2bin124	Structural complex_ Environmental information processing_ Saccharide, polyol, and lipid transport system_Raffinose/stachyose/melibiose transport system	0,006	0,055	0,429
Bacteria_Actinobacteriota_Actinomycetia_Actinomycetales_Bifidobacteriaceae_Bifidobacterium_Bifidobacterium adolescentis_MB2bin135	Structural complex_ Environmental information processing_ Saccharide, polyol, and lipid transport system_Raffinose/stachyose/melibiose transport system	0,000	0,000	0,797
Bacteria_Bacteroidota_Bacteroidia_Bacteroidales_Bacteroidaceae_Bacteroides_Bacteroides thetaiotaomicron_MB2bin137	Structural complex_ Environmental information processing_ Saccharide, polyol, and lipid transport system_Raffinose/stachyose/melibiose transport system	0,001	0,020	-0,505
Bacteria_Bacteroidota_Bacteroidia_Bacteroidales_Bacteroidaceae_Bacteroides_Bacteroides eggerthii_MB2bin146	Structural complex_ Environmental information processing_ Saccharide, polyol, and lipid transport system_Raffinose/stachyose/melibiose transport system	0,005	0,050	-0,439
Bacteria_Firmicutes_A_Clostridia_Oscillospirales_Oscillospiraceae_Oscillibacter_?_MB2bin147	Structural complex_ Environmental information processing_ Saccharide, polyol, and lipid transport system_Raffinose/stachyose/melibiose transport system	0,010	0,070	-0,408
Bacteria_Firmicutes_A_Clostridia_Oscillospirales_Oscillospiraceae_CAG-83_?_MB2bin179	Structural complex_ Environmental information processing_ Saccharide, polyol, and lipid transport system_Raffinose/stachyose/melibiose transport system	0,006	0,053	-0,433
Bacteria_Bacteroidota_Bacteroidia_Bacteroidales_Tannerellaceae_Parabacteroides_Parabacteroides johnsonii_MB2bin185	Structural complex_ Environmental information processing_ Saccharide, polyol, and lipid transport system_Raffinose/stachyose/melibiose transport system	0,009	0,067	-0,413
Bacteria_Actinobacteriota_Coriobacteriia_Coriobacteriales_Coriobacteriaceae_Collinsella_Collinsella sp000763055_MB2bin202	Structural complex_ Environmental information processing_ Saccharide, polyol, and lipid transport system_Raffinose/stachyose/melibiose transport system	0,005	0,050	0,440

Bacteria_Actinobacteriota_Actinomycetia_Actinomycetales_Bifidobacteriaceae_Bifidobacterium_Bifidobacterium pseudocatenulatum_MB2bin265	Structural complex_ Environmental information processing_ Saccharide, polyol, and lipid transport system_Raffinose/stachyose/melibiose transport system	0,003	0,043	0,462
Bacteria_Firmicutes_A_Clostridia_A_Christensenellales_QAND01_UMGS1975_UMGS1975 sp900546685_MB2bin272	Structural complex_ Environmental information processing_ Saccharide, polyol, and lipid transport system_Raffinose/stachyose/melibiose transport system	0,001	0,018	0,517
Bacteria_Bacteroidota_Bacteroidia_Bacteroidales_Rikenellaceae_Alistipes_Alistipes obesi_MB2bin28	Structural complex_ Environmental information processing_ Saccharide, polyol, and lipid transport system_Raffinose/stachyose/melibiose transport system	0,000	0,003	-0,578
Bacteria_Actinobacteriota_Actinomycetia_Actinomycetales_Bifidobacteriaceae_Bifidobacterium_Bifidobacterium angulatum_MB2bin288	Structural complex_ Environmental information processing_ Saccharide, polyol, and lipid transport system_Raffinose/stachyose/melibiose transport system	0,000	0,000	0,709
Bacteria_Bacteroidota_Bacteroidia_Bacteroidales_Bacteroidaceae_Phocaeicola_Phocaeicola vulgatus_MB2bin4	Structural complex_ Environmental information processing_ Saccharide, polyol, and lipid transport system_Raffinose/stachyose/melibiose transport system	0,000	0,001	-0,607
Bacteria_Bacteroidota_Bacteroidia_Bacteroidales_Bacteroidaceae_Bacteroides_Bacteroides stercoris_MB2bin42	Structural complex_ Environmental information processing_ Saccharide, polyol, and lipid transport system_Raffinose/stachyose/melibiose transport system	0,004	0,044	-0,455
Bacteria_Bacteroidota_Bacteroidia_Bacteroidales_Rikenellaceae_Alistipes_Alistipes finegoldii_MB2bin46	Structural complex_ Environmental information processing_ Saccharide, polyol, and lipid transport system_Raffinose/stachyose/melibiose transport system	0,005	0,050	-0,441
Bacteria_Firmicutes_A_Clostridia_Lachnospirales_Lachnospiraceae_Anaerostipes_Anaerostipes hadrus_MB2bin47	Structural complex_ Environmental information processing_ Saccharide, polyol, and lipid transport system_Raffinose/stachyose/melibiose transport system	0,001	0,020	0,510
Bacteria_Bacteroidota_Bacteroidia_Bacteroidales_Tannerellaceae_Parabacteroides_Parabacteroides merdae_MB2bin5	Structural complex_ Environmental information processing_ Saccharide, polyol, and lipid transport	0,009	0,067	-0,415

	system_Raffinose/stachyose/melibiose transport system			
Bacteria_Actinobacteriota_Coriobacteriia_Coriobacteriales_Coriobacteriaceae_Collinsella_?_MB2bin75	Structural complex_ Environmental information processing_ Saccharide, polyol, and lipid transport system_Raffinose/stachyose/melibiose transport system	0,007	0,055	0,427
Bacteria_Firmicutes_A_Clostridia_Lachnospirales_Lachnospiraceae_Ruminococcus_A_Ruminococcus_A sp003011855_MB2bin82	Structural complex_ Environmental information processing_ Saccharide, polyol, and lipid transport system_Raffinose/stachyose/melibiose transport system	0,015	0,096	0,388
Bacteria_Bacteroidota_Bacteroidia_Bacteroidales_Rikenellaceae_Alistipes_Alistipes sp900083545_specI_v3_Cluster1016	Structural complex_ Environmental information processing_ Saccharide, polyol, and lipid transport system_Raffinose/stachyose/melibiose transport system	0,010	0,070	-0,407
Bacteria_Bacteroidota_Bacteroidia_Bacteroidales_Bacteroidaceae_Bacteroides_B_Bacteroides_B sartorii_specI_v3_Cluster2366	Structural complex_ Environmental information processing_ Saccharide, polyol, and lipid transport system_Raffinose/stachyose/melibiose transport system	0,000	0,001	-0,617
Bacteria_Actinobacteriota_Actinobacteria_Actinomycetales_Bifidobacteriaceae_Bifidobacterium_Bifidobacterium ruminantium_specI_v3_Cluster2702	Structural complex_ Environmental information processing_ Saccharide, polyol, and lipid transport system_Raffinose/stachyose/melibiose transport system	0,000	0,000	0,712
Bacteria_Firmicutes_A_Clostridia_Lachnospirales_Lachnospiraceae_Anaerostipes_Anaerostipes hadrus_A_specI_v3_Cluster856	Structural complex_ Environmental information processing_ Saccharide, polyol, and lipid transport system_Raffinose/stachyose/melibiose transport system	0,003	0,043	0,460
Bacteria_Actinobacteriota_Actinomycetia_Actinomycetales_Bifidobacteriaceae_Bifidobacterium_Bifidobacterium adolescentis_MB2bin135	Structural complex_ Genetic information processing_ RNA processing_RNA degradosome	0,000	0,008	0,585
Bacteria_Actinobacteriota_Actinomycetia_Actinomycetales_Bifidobacteriaceae_Bifidobacterium_Bifidobacterium longum_MB2bin141	Structural complex_ Genetic information processing_ RNA processing_RNA degradosome	0,000	0,008	0,590

Bacteria_Actinobacteriota_Actinomycetia_Actinomycetales_Bifidobacteriaceae_Bifidobacterium_Bifidobacterium bifidum_MB2bin203	Structural complex_ Genetic information processing_ RNA processing_RNA degradosome	0,002	0,044	0,490
Bacteria_Actinobacteriota_Actinomycetia_Actinomycetales_Bifidobacteriaceae_Bifidobacterium_Bifidobacterium pseudocatenulatum_MB2bin265	Structural complex_ Genetic information processing_ RNA processing_RNA degradosome	0,001	0,030	0,530
Bacteria_Actinobacteriota_Actinomycetia_Actinomycetales_Bifidobacteriaceae_Bifidobacterium_Bifidobacterium angulatum_MB2bin288	Structural complex_ Genetic information processing_ RNA processing_RNA degradosome	0,001	0,044	0,497
Bacteria_Firmicutes_A_Clostridia_Lachnospirales_Lachnospiraceae_Anaerostipes_Anaerostipes hadrus_MB2bin47	Structural complex_ Genetic information processing_ RNA processing_RNA degradosome	0,002	0,047	0,482
Bacteria_Actinobacteriota_Actinobacteria_Actinomycetales_Bifidobacteriaceae_Bifidobacterium_Bifidobacterium breve_specI_v3_Cluster1098	Structural complex_ Genetic information processing_ RNA processing_RNA degradosome	0,001	0,030	0,520
Bacteria_Actinobacteriota_Actinobacteria_Actinomycetales_Bifidobacteriaceae_Bifidobacterium_Bifidobacterium ruminantium_specI_v3_Cluster2702	Structural complex_ Genetic information processing_ RNA processing_RNA degradosome	0,003	0,054	0,470
Bacteria_Bacteroidota_Bacteroidia_Bacteroidales_Bacteroidaceae_Prevotella_Prevotella copri_A_MB2bin109	Signature module_ Gene set_ Metabolic capacity_Acetogen	0,002	0,036	0,487
Bacteria_Firmicutes_A_Clostridia_A_Christensenellales_CAG-74_SFFH01_SFFH01 sp900542445_MB2bin113	Signature module_ Gene set_ Metabolic capacity_Acetogen	0,005	0,048	0,445
Bacteria_Firmicutes_A_Clostridia_Lachnospirales_Lachnospirales_Dorea_Dorea formicigenerans_MB2bin120	Signature module_ Gene set_ Metabolic capacity_Acetogen	0,002	0,036	0,487
Bacteria_Verrucomicrobiota_Verrucomicrobiae_Verrucomicrobiales_Akkermansiaceae_Akkermansia_Akkermansia muciniphila_B_MB2bin125	Signature module_ Gene set_ Metabolic capacity_Acetogen	0,004	0,042	-0,454
Bacteria_Firmicutes_A_Clostridia_Lachnospirales_Lachnospiraceae_Mediterraneibacter_Mediterraneibacter faecis_MB2bin127	Signature module_ Gene set_ Metabolic capacity_Acetogen	0,013	0,094	0,394

Bacteria_Firmicutes_I_Bacilli_A_Lactobacillales_Streptococcaceae_Streptococcus_Streptococcus thermophilus_MB2bin145	Signature module_ Gene set_ Metabolic capacity_Acetogen	0,001	0,036	0,494
Bacteria_Bacteroidota_Bacteroidia_Bacteroidales_Bacteroidaceae_Bacteroides_Bacteroides eggerthii_MB2bin146	Signature module_ Gene set_ Metabolic capacity_Acetogen	0,015	0,099	-0,387
Bacteria_Firmicutes_A_Clostridia_Lachnospirales_Lachnospiraceae_Coprococcus_Coprococcus eutactus_MB2bin153	Signature module_ Gene set_ Metabolic capacity_Acetogen	0,001	0,036	0,497
Bacteria_Firmicutes_A_Clostridia_Lachnospirales_Lachnospiraceae_Ruminococcus_A_Ruminococcus_A sp000437095_MB2bin155	Signature module_ Gene set_ Metabolic capacity_Acetogen	0,001	0,036	0,504
Bacteria_Firmicutes_A_Clostridia_Lachnospirales_Lachnospiraceae_Acetatifactor_Acetatifactor sp900066565_MB2bin18	Signature module_ Gene set_ Metabolic capacity_Acetogen	0,012	0,090	0,401
Bacteria_Firmicutes_A_Clostridia_Lachnospirales_Lachnospiraceae_Bariatricus_Bariatricus comes_MB2bin19	Signature module_ Gene set_ Metabolic capacity_Acetogen	0,001	0,036	0,509
Bacteria_Firmicutes_A_Clostridia_Lachnospirales_Lachnospiraceae_Mediterraneibacter_Mediterraneibacter lactaris_MB2bin26	Signature module_ Gene set_ Metabolic capacity_Acetogen	0,006	0,059	0,433
Bacteria_Firmicutes_A_Clostridia_Lachnospirales_Lachnospiraceae_Roseburia_Roseburia hominis_MB2bin32	Signature module_ Gene set_ Metabolic capacity_Acetogen	0,013	0,094	0,395
Bacteria_Firmicutes_A_Clostridia_Lachnospirales_Lachnospiraceae_Coprococcus_A_Coprococcus_A catus_MB2bin38	Signature module_ Gene set_ Metabolic capacity_Acetogen	0,002	0,037	0,477
Bacteria_Firmicutes_A_Clostridia_Oscillospirales_Ruminococcaceae_Gemmiger_Gemmiger sp900539695_MB2bin39	Signature module_ Gene set_ Metabolic capacity_Acetogen	0,002	0,037	0,479
Bacteria_Bacteroidota_Bacteroidia_Bacteroidales_Bacteroidaceae_Bacteroides_Bacteroides stercoris_MB2bin42	Signature module_ Gene set_ Metabolic capacity_Acetogen	0,014	0,097	-0,390
Bacteria_Firmicutes_A_Clostridia_Oscillospirales_Ruminococcaceae_Gemmiger_Gemmiger sp900540595_MB2bin67	Signature module_ Gene set_ Metabolic capacity_Acetogen	0,007	0,064	0,425
Bacteria_Firmicutes_A_Clostridia_Lachnospirales_Lachnospiraceae_Coprococcus_Coprococcus eutactus_A_MB2bin7	Signature module_ Gene set_ Metabolic capacity_Acetogen	0,003	0,042	0,459

Bacteria_Firmicutes_A_Clostridia_Oscillospirales_Butyricicoccaceae_Agathobaculum_Agathobaculum butyriciproducens_MB2bin73	Signature module_ Gene set_ Metabolic capacity_Acetogen	0,001	0,036	0,504
Bacteria_Firmicutes_A_Clostridia_Oscillospirales_Ruminococcaceae_Faecalibacterium_Faecalibacterium prausnitzii_A_MB2bin78	Signature module_ Gene set_ Metabolic capacity_Acetogen	0,003	0,042	0,459
Bacteria_Firmicutes_A_Clostridia_Lachnospirales_Lachnospiraceae_Blautia_A_Blautia_A massiliensis_MB2bin79	Signature module_ Gene set_ Metabolic capacity_Acetogen	0,011	0,089	0,404
Bacteria_Firmicutes_A_Clostridia_Lachnospirales_Lachnospirales_Dorea_Dorea longicatena_MB2bin81	Signature module_ Gene set_ Metabolic capacity_Acetogen	0,004	0,042	0,455
Bacteria_Firmicutes_A_Clostridia_Lachnospirales_Lachnospiraceae_Ruminococcus_A_Ruminococcus_A sp003011855_MB2bin82	Signature module_ Gene set_ Metabolic capacity_Acetogen	0,001	0,036	0,509
Bacteria_Firmicutes_A_Clostridia_Oscillospirales_Acutalibacteraceae_Ruminococcus_H_Ruminococcus_H sp003531055_MB2bin93	Signature module_ Gene set_ Metabolic capacity_Acetogen	0,004	0,042	0,456
Bacteria_Firmicutes_A_Clostridia_Lachnospirales_Lachnospiraceae_Blautia_A_Blautia_A sp900066355_MGS00045	Signature module_ Gene set_ Metabolic capacity_Acetogen	0,009	0,075	0,415
Bacteria_Firmicutes_A_Clostridia_TANB77_CAG-508_CAG-269_CAG-269 sp000437215_specI_v3_Cluster7628	Signature module_ Gene set_ Metabolic capacity_Acetogen	0,006	0,060	0,430
Bacteria_Firmicutes_A_Clostridia_Oscillospirales_Ruminococcaceae_Faecalibacterium_Faecalibacterium prausnitzii_D_MB2bin102	Structural complex_ Environmental information processing_ Mineral and organic ion transport system_Spermidine/putrescine transport system	0,004	0,100	0,452
Bacteria_Bacteroidota_Bacteroidia_Bacteroidales_Bacteroidaceae_Prevotella_Prevotella copri_A_MB2bin109	Structural complex_ Environmental information processing_ Mineral and organic ion transport system_Spermidine/putrescine transport system	0,004	0,100	0,451
Bacteria_Firmicutes_A_Clostridia_Lachnospirales_Lachnospiraceae_Coprococcus_Coprococcus eutactus_MB2bin153	Structural complex_ Environmental information processing_ Mineral and organic ion transport system_Spermidine/putrescine transport system	0,002	0,098	0,474

Bacteria_Firmicutes_C_Negativicutes_Acidaminococcales_Acidaminococcaceae_Phascolarctobacterium_A_?_MB2bin381	Structural complex_ Environmental information processing_ Mineral and organic ion transport system_Spermidine/putrescine transport system	0,002	0,098	0,477
Bacteria_Firmicutes_A_Clostridia_Lachnospirales_Lachnospirales_Dorea_Dorea longicatena_MB2bin81	Structural complex_ Environmental information processing_ Mineral and organic ion transport system_Spermidine/putrescine transport system	0,001	0,098	0,501
Bacteria_Firmicutes_A_Clostridia_Oscillospirales_Ruminococcaceae_Faecalibacterium_Faecalibacterium prausnitzii_C_MB2bin90	Structural complex_ Environmental information processing_ Mineral and organic ion transport system_Spermidine/putrescine transport system	0,004	0,100	0,450
Bacteria_Firmicutes_C_Negativicutes_Veillonellales_Dialisteraceae_Dialister_Dialister invisus_specI_v3_Cluster3691	Structural complex_ Environmental information processing_ Mineral and organic ion transport system_Spermidine/putrescine transport system	0,002	0,098	-0,480
Bacteria_Bacteroidota_Bacteroidia_Bacteroidales_Barnesiellaceae_Barnesiella_Barnesiella intestinihominis_MB2bin112	Pathway module_ Carbohydrate and lipid metabolism_ Fatty acid metabolism_Fatty acid biosynthesis, elongation	0,003	0,071	-0,459
Bacteria_Actinobacteriota_Actinomycetia_Actinomycetales_Bifidobacteriaceae_Bifidobacterium_Bifidobacterium adolescentis_MB2bin135	Pathway module_ Carbohydrate and lipid metabolism_ Fatty acid metabolism_Fatty acid biosynthesis, elongation	0,000	0,000	0,720
Bacteria_Actinobacteriota_Actinomycetia_Actinomycetales_Bifidobacteriaceae_Bifidobacterium_Bifidobacterium longum_MB2bin141	Pathway module_ Carbohydrate and lipid metabolism_ Fatty acid metabolism_Fatty acid biosynthesis, elongation	0,000	0,000	0,666
Bacteria_Actinobacteriota_Actinomycetia_Actinomycetales_Bifidobacteriaceae_Bifidobacterium_Bifidobacterium pseudocatenulatum_MB2bin265	Pathway module_ Carbohydrate and lipid metabolism_ Fatty acid metabolism_Fatty acid biosynthesis, elongation	0,001	0,023	0,508
Bacteria_Firmicutes_A_Clostridia_A_Christensenellales_QAND01_UMGS1975_UMGS1975 sp900546685_MB2bin272	Pathway module_ Carbohydrate and lipid metabolism_ Fatty acid metabolism_Fatty acid biosynthesis, elongation	0,001	0,017	0,525
Bacteria_Actinobacteriota_Actinomycetia_Actinomycetales_Bifidobacteriaceae_Bifidobacterium_Bifidobacterium angulatum_MB2bin288	Pathway module_ Carbohydrate and lipid metabolism_ Fatty acid metabolism_Fatty acid biosynthesis, elongation	0,000	0,000	0,651

Bacteria_Actinobacteriota_Actinobacteria_Actinomycetales_Bifidobacteriaceae_Bifidobacterium_Bifidobacterium breve_specI_v3_Cluster1098	Pathway module_ Carbohydrate and lipid metabolism_ Fatty acid metabolism_Fatty acid biosynthesis, elongation	0,000	0,003	0,583
Bacteria_Actinobacteriota_Actinobacteria_Actinomycetales_Bifidobacteriaceae_Bifidobacterium_Bifidobacterium ruminantium_specI_v3_Cluster2702	Pathway module_ Carbohydrate and lipid metabolism_ Fatty acid metabolism_Fatty acid biosynthesis, elongation	0,000	0,001	0,632
Bacteria_Bacteroidota_Bacteroidia_Bacteroidales_Barnesiellaceae_Barnesiella_Barnesiella intestinihominis_MB2bin112	Pathway module_ Carbohydrate and lipid metabolism_ Fatty acid metabolism_Fatty acid biosynthesis, initiation	0,004	0,080	-0,453
Bacteria_Actinobacteriota_Actinomycetia_Actinomycetales_Bifidobacteriaceae_Bifidobacterium_Bifidobacterium adolescentis_MB2bin135	Pathway module_ Carbohydrate and lipid metabolism_ Fatty acid metabolism_Fatty acid biosynthesis, initiation	0,000	0,000	0,728
Bacteria_Actinobacteriota_Actinomycetia_Actinomycetales_Bifidobacteriaceae_Bifidobacterium_Bifidobacterium longum_MB2bin141	Pathway module_ Carbohydrate and lipid metabolism_ Fatty acid metabolism_Fatty acid biosynthesis, initiation	0,000	0,010	0,551
Bacteria_Actinobacteriota_Actinomycetia_Actinomycetales_Bifidobacteriaceae_Bifidobacterium_Bifidobacterium pseudocatenulatum_MB2bin265	Pathway module_ Carbohydrate and lipid metabolism_ Fatty acid metabolism_Fatty acid biosynthesis, initiation	0,006	0,094	0,432
Bacteria_Firmicutes_A_Clostridia_A_Christensenellales_QAND01_UMGS1975_UMGS1975 sp900546685_MB2bin272	Pathway module_ Carbohydrate and lipid metabolism_ Fatty acid metabolism_Fatty acid biosynthesis, initiation	0,000	0,001	0,615
Bacteria_Bacteroidota_Bacteroidia_Bacteroidales_Rikenellaceae_Alistipes_Alistipes obesi_MB2bin28	Pathway module_ Carbohydrate and lipid metabolism_ Fatty acid metabolism_Fatty acid biosynthesis, initiation	0,003	0,064	-0,469
Bacteria_Actinobacteriota_Actinomycetia_Actinomycetales_Bifidobacteriaceae_Bifidobacterium_Bifidobacterium angulatum_MB2bin288	Pathway module_ Carbohydrate and lipid metabolism_ Fatty acid metabolism_Fatty acid biosynthesis, initiation	0,000	0,000	0,680
Bacteria_Bacteroidota_Bacteroidia_Bacteroidales_Bacteroidaceae_Phocaeicola_Phocaeicola vulgatus_MB2bin4	Pathway module_ Carbohydrate and lipid metabolism_ Fatty acid metabolism_Fatty acid biosynthesis, initiation	0,005	0,092	-0,437
Bacteria_Bacteroidota_Bacteroidia_Bacteroidales_Rikenellaceae_Alistipes_Alistipes finegoldii_MB2bin46	Pathway module_ Carbohydrate and lipid metabolism_ Fatty acid metabolism_Fatty acid biosynthesis, initiation	0,003	0,064	-0,469

Bacteria_Actinobacteriota_Actinobacteria_Actinomycetales_Bifidobacteriaceae_Bifidobacterium_Bifidobacterium breve_specI_v3_Cluster1098	Pathway module_ Carbohydrate and lipid metabolism_ Fatty acid metabolism_Fatty acid biosynthesis, initiation	0,004	0,080	0,449
Bacteria_Actinobacteriota_Actinobacteria_Actinomycetales_Bifidobacteriaceae_Bifidobacterium_Bifidobacterium ruminantium_specI_v3_Cluster2702	Pathway module_ Carbohydrate and lipid metabolism_ Fatty acid metabolism_Fatty acid biosynthesis, initiation	0,000	0,000	0,675
Bacteria_Bacteroidota_Bacteroidia_Bacteroidales_Rikenellaceae_Alistipes_Alistipes shahii_MB2bin10	Pathway module_ Carbohydrate and lipid metabolism_ Central carbohydrate metabolism_Pyruvate oxidation, pyruvate => acetyl-CoA	0,000	0,034	0,539
Bacteria_Firmicutes_A_Clostridia_Oscillospirales_Oscillospiraceae_CAG-83_?_MB2bin100	Pathway module_ Carbohydrate and lipid metabolism_ Central carbohydrate metabolism_Pyruvate oxidation, pyruvate => acetyl-CoA	0,001	0,040	0,493
Bacteria_Firmicutes_A_Clostridia_Oscillospirales_Oscillospiraceae_CAG-103_?_MB2bin101	Pathway module_ Carbohydrate and lipid metabolism_ Central carbohydrate metabolism_Pyruvate oxidation, pyruvate => acetyl-CoA	0,002	0,040	0,488
Bacteria_Bacteroidota_Bacteroidia_Bacteroidales_Bacteroidaceae_Paraprevotella_Paraprevotella clara_MB2bin115	Pathway module_ Carbohydrate and lipid metabolism_ Central carbohydrate metabolism_Pyruvate oxidation, pyruvate => acetyl-CoA	0,004	0,062	0,451
Bacteria_Actinobacteriota_Actinomycetia_Actinomycetales_Bifidobacteriaceae_Bifidobacterium_Bifidobacterium adolescentis_MB2bin135	Pathway module_ Carbohydrate and lipid metabolism_ Central carbohydrate metabolism_Pyruvate oxidation, pyruvate => acetyl-CoA	0,002	0,044	-0,479
Bacteria_Bacteroidota_Bacteroidia_Bacteroidales_Tannerellaceae_Parabacteroides_Parabacteroides johnsonii_MB2bin185	Pathway module_ Carbohydrate and lipid metabolism_ Central carbohydrate metabolism_Pyruvate oxidation, pyruvate => acetyl-CoA	0,003	0,051	0,463
Bacteria_Firmicutes_A_Clostridia_Oscillospirales_Ruminococcaceae_Ruminiclostridium_E_Ruminiclostridium_E sp003512525_MB2bin228	Pathway module_ Carbohydrate and lipid metabolism_ Central carbohydrate metabolism_Pyruvate oxidation, pyruvate => acetyl-CoA	0,006	0,085	0,429
Bacteria_Firmicutes_A_Clostridia_A_Christensenellales_QAND01_UMGS1975_UMGS1975 sp900546685_MB2bin272	Pathway module_ Carbohydrate and lipid metabolism_ Central carbohydrate metabolism_Pyruvate oxidation, pyruvate => acetyl-CoA	0,002	0,040	-0,490

Bacteria_Firmicutes_A_Clostridia_Oscillospirales_Ruminococcaceae_Faecalibacterium_Faecalibacterium prausnitzii_G_MB2bin45	Pathway module_ Carbohydrate and lipid metabolism_ Central carbohydrate metabolism_Pyruvate oxidation, pyruvate => acetyl-CoA	0,001	0,040	-0,500
Bacteria_Firmicutes_A_Clostridia_Lachnospirales_Lachnospiraceae_Anaerostipes_Anaerostipes hadrus_MB2bin47	Pathway module_ Carbohydrate and lipid metabolism_ Central carbohydrate metabolism_Pyruvate oxidation, pyruvate => acetyl-CoA	0,003	0,051	-0,466
Bacteria_Bacteroidota_Bacteroidia_Bacteroidales_Tannerellaceae_Parabacteroides_Parabacteroides merdae_MB2bin5	Pathway module_ Carbohydrate and lipid metabolism_ Central carbohydrate metabolism_Pyruvate oxidation, pyruvate => acetyl-CoA	0,001	0,040	0,510
Bacteria_Firmicutes_A_Clostridia_Oscillospirales_Ruminococcaceae_Ruminiclostridium_E_Ruminiclostridium_E siraeum_MB2bin52	Pathway module_ Carbohydrate and lipid metabolism_ Central carbohydrate metabolism_Pyruvate oxidation, pyruvate => acetyl-CoA	0,005	0,078	0,437
Bacteria_Bacteroidota_Bacteroidia_Bacteroidales_Rikenellaceae_Alistipes_Alistipes sp900083545_specI_v3_Cluster1016	Pathway module_ Carbohydrate and lipid metabolism_ Central carbohydrate metabolism_Pyruvate oxidation, pyruvate => acetyl-CoA	0,000	0,003	0,626
Bacteria_Verrucomicrobiota_Verrucomicrobiae_Verrucomicrobiales_Akkermansiaceae_Akkermansia_Akkermansia muciniphila_B_MB2bin125	Pathway module_ Nucleotide and amino acid metabolism_ Pyrimidine metabolism_Pyrimidine ribonucleotide biosynthesis, UMP => UDP/UTP,CDP/CTP	0,002	0,054	-0,489
Bacteria_Actinobacteriota_Actinomycetia_Actinomycetales_Bifidobacteriaceae_Bifidobacterium_Bifidobacterium adolescentis_MB2bin135	Pathway module_ Nucleotide and amino acid metabolism_ Pyrimidine metabolism_Pyrimidine ribonucleotide biosynthesis, UMP => UDP/UTP,CDP/CTP	0,003	0,077	-0,461
Bacteria_Firmicutes_A_Clostridia_Oscillospirales_Oscillospiraceae_CAG-170_CAG-170 sp003516765_MB2bin143	Pathway module_ Nucleotide and amino acid metabolism_ Pyrimidine metabolism_Pyrimidine ribonucleotide biosynthesis, UMP => UDP/UTP,CDP/CTP	0,001	0,044	0,516
Bacteria_Firmicutes_A_Clostridia_Oscillospirales_Ruminococcaceae_Gemmiger_Gemmiger formicilis_MB2bin15	Pathway module_ Nucleotide and amino acid metabolism_ Pyrimidine metabolism_Pyrimidine ribonucleotide biosynthesis, UMP => UDP/UTP,CDP/CTP	0,006	0,077	-0,431
Bacteria_Firmicutes_A_Clostridia_Lachnospirales_Lachnospiraceae	Pathway module_ Nucleotide and amino acid metabolism_ Pyrimidine	0,001	0,044	0,520

_Acetatifactor_Acetatifactor sp900066565_MB2bin18	metabolism_Pyrimidine ribonucleotide biosynthesis, UMP => UDP/UTP,CDP/CTP			
Bacteria_Actinobacteriota_Coriobacteriia_Coriobacteriales_Eggerthellaceae_CAG-1427_CAG-1427 sp000436075_MB2bin206	Pathway module_ Nucleotide and amino acid metabolism_ Pyrimidine metabolism_Pyrimidine ribonucleotide biosynthesis, UMP => UDP/UTP,CDP/CTP	0,002	0,061	0,477
Bacteria_Firmicutes_A_Clostridia_Lachnospirales_Lachnospiraceae_CAG-45_CAG-45 sp000438375_MB2bin229	Pathway module_ Nucleotide and amino acid metabolism_ Pyrimidine metabolism_Pyrimidine ribonucleotide biosynthesis, UMP => UDP/UTP,CDP/CTP	0,006	0,077	0,433
Bacteria_Firmicutes_A_Clostridia_Peptostreptococcales_Anaerovoracaceae_Mogibacterium_Mogibacterium sp002299625_MB2bin244	Pathway module_ Nucleotide and amino acid metabolism_ Pyrimidine metabolism_Pyrimidine ribonucleotide biosynthesis, UMP => UDP/UTP,CDP/CTP	0,000	0,035	-0,561
Archaea_Methanobacteriota_Methanobacteria_Methanobacteriales_Methanobacteriaceae_Methanobrevibacter_A_Methanobrevibacter_A smithii_MB2bin267	Pathway module_ Nucleotide and amino acid metabolism_ Pyrimidine metabolism_Pyrimidine ribonucleotide biosynthesis, UMP => UDP/UTP,CDP/CTP	0,004	0,077	-0,451
Bacteria_Firmicutes_A_Clostridia_Oscillospirales_Acutalibacteraceae_Eubacterium_R_Eubacterium_R sp000434995_MB2bin275	Pathway module_ Nucleotide and amino acid metabolism_ Pyrimidine metabolism_Pyrimidine ribonucleotide biosynthesis, UMP => UDP/UTP,CDP/CTP	0,005	0,077	0,437
Bacteria_Firmicutes_A_Clostridia_A_Christensenellales_CAG-74_UBA11524_UBA11524 sp000437595_MB2bin287	Pathway module_ Nucleotide and amino acid metabolism_ Pyrimidine metabolism_Pyrimidine ribonucleotide biosynthesis, UMP => UDP/UTP,CDP/CTP	0,007	0,077	0,424
Bacteria_Firmicutes_A_Clostridia_Oscillospirales_Ruminococcaceae_Faecalibacterium_Faecalibacterium sp900539945_MB2bin59	Pathway module_ Nucleotide and amino acid metabolism_ Pyrimidine metabolism_Pyrimidine ribonucleotide biosynthesis, UMP => UDP/UTP,CDP/CTP	0,007	0,077	0,427
Bacteria_Verrucomicrobiota_Verrucomicrobiae_Verrucomicrobiales_Akkermansiaceae_Akkermansia_Akkermansia muciniphila_MB2bin64	Pathway module_ Nucleotide and amino acid metabolism_ Pyrimidine metabolism_Pyrimidine ribonucleotide biosynthesis, UMP => UDP/UTP,CDP/CTP	0,002	0,054	-0,489

Bacteria_Firmicutes_A_Clostridia_Oscillospirales_Butyricicoccaceae_Agathobaculum_Agathobaculum butyriciproducens_MB2bin73	Pathway module_ Nucleotide and amino acid metabolism_ Pyrimidine metabolism_Pyrimidine ribonucleotide biosynthesis, UMP => UDP/UTP,CDP/CTP	0,007	0,077	0,428
Bacteria_Firmicutes_A_Clostridia_Lachnospirales_Lachnospiraceae_Acetatifactor_Acetatifactor sp900066365_MB2bin8	Pathway module_ Nucleotide and amino acid metabolism_ Pyrimidine metabolism_Pyrimidine ribonucleotide biosynthesis, UMP => UDP/UTP,CDP/CTP	0,004	0,077	0,446
Bacteria_Firmicutes_I_Bacilli_A_Erysipelotrichales_Erysipelotrichaceae_Absiella_Absiella sp000163515_specI_v3_Cluster2380	Pathway module_ Nucleotide and amino acid metabolism_ Pyrimidine metabolism_Pyrimidine ribonucleotide biosynthesis, UMP => UDP/UTP,CDP/CTP	0,007	0,077	0,428
Bacteria_Firmicutes_A_Clostridia_Oscillospirales_Ruminococcaceae_Faecalibacterium_Faecalibacterium prausnitzii_D_MB2bin102	Functional set_ Environmental information processing_ Drug resistance_Aminoglycoside resistance, protease FtsH	0,010	0,074	0,408
Bacteria_Bacteroidota_Bacteroidia_Bacteroidales_Bacteroidaceae_Prevotella_Prevotella copri_A_MB2bin109	Functional set_ Environmental information processing_ Drug resistance_Aminoglycoside resistance, protease FtsH	0,004	0,053	0,446
Bacteria_Firmicutes_A_Clostridia_A_Christensenellales_CAG-74_SFFH01_SFFH01 sp900542445_MB2bin113	Functional set_ Environmental information processing_ Drug resistance_Aminoglycoside resistance, protease FtsH	0,000	0,021	0,538
Bacteria_Firmicutes_A_Clostridia_Lachnospirales_Lachnospirales_Dorea_Dorea formicigenerans_MB2bin120	Functional set_ Environmental information processing_ Drug resistance_Aminoglycoside resistance, protease FtsH	0,001	0,024	0,507
Bacteria_Verrucomicrobiota_Verrucomicrobiae_Verrucomicrobiales_Akkermansiaceae_Akkermansia_Akkermansia muciniphila_B_MB2bin125	Functional set_ Environmental information processing_ Drug resistance_Aminoglycoside resistance, protease FtsH	0,000	0,021	-0,532
Bacteria_Firmicutes_A_Clostridia_Lachnospirales_Lachnospiraceae_Mediterraneibacter_Mediterraneibacter faecis_MB2bin127	Functional set_ Environmental information processing_ Drug resistance_Aminoglycoside resistance, protease FtsH	0,005	0,053	0,441
Bacteria_Bacteroidota_Bacteroidia_Bacteroidales_Bacteroidaceae_Bacteroides_Bacteroides thetaiotaomicron_MB2bin137	Functional set_ Environmental information processing_ Drug resistance_Aminoglycoside resistance, protease FtsH	0,009	0,073	-0,411
Bacteria_Firmicutes_A_Clostridia_Lachnospirales_Lachnospiraceae_Ruminococcus_A_Ruminococcus_A sp000437095_MB2bin155	Functional set_ Environmental information processing_ Drug resistance_Aminoglycoside resistance, protease FtsH	0,001	0,027	0,494

Bacteria_Firmicutes_A_Clostridia_Lachnospirales_Lachnospiraceae_KLE1615_KLE1615 sp900066985_MB2bin161	Functional set_ Environmental information processing_ Drug resistance_Aminoglycoside resistance, protease FtsH	0,004	0,053	0,446
Bacteria_Bacteroidota_Bacteroidia_Bacteroidales_Bacteroidaceae_Bacteroides_Bacteroides fragilis_MB2bin178	Functional set_ Environmental information processing_ Drug resistance_Aminoglycoside resistance, protease FtsH	0,012	0,088	-0,397
Bacteria_Firmicutes_A_Clostridia_Lachnospirales_Lachnospiraceae_Bariatricus_Bariatricus comes_MB2bin19	Functional set_ Environmental information processing_ Drug resistance_Aminoglycoside resistance, protease FtsH	0,002	0,039	0,471
Bacteria_Actinobacteriota_Actinomycetia_Actinomycetales_Bifidobacteriaceae_Bifidobacterium_Bifidobacterium bifidum_MB2bin203	Functional set_ Environmental information processing_ Drug resistance_Aminoglycoside resistance, protease FtsH	0,001	0,024	-0,509
Bacteria_Actinobacteriota_Coriobacteriia_Coriobacteriales_Eggerthellaceae_CAG-1427_CAG-1427 sp000436075_MB2bin206	Functional set_ Environmental information processing_ Drug resistance_Aminoglycoside resistance, protease FtsH	0,001	0,026	0,500
Bacteria_Firmicutes_A_Clostridia_Lachnospirales_Lachnospiraceae_Blautia_A_Blautia_A sp900066335_MB2bin25	Functional set_ Environmental information processing_ Drug resistance_Aminoglycoside resistance, protease FtsH	0,007	0,067	0,425
Bacteria_Firmicutes_A_Clostridia_Lachnospirales_Lachnospiraceae_Mediterraneibacter_Mediterraneibacter lactaris_MB2bin26	Functional set_ Environmental information processing_ Drug resistance_Aminoglycoside resistance, protease FtsH	0,005	0,054	0,438
Bacteria_Firmicutes_A_Clostridia_Oscillospirales_Ruminococcaceae_Faecalibacterium_Faecalibacterium prausnitzii_H_MB2bin261	Functional set_ Environmental information processing_ Drug resistance_Aminoglycoside resistance, protease FtsH	0,005	0,053	0,443
Bacteria_Firmicutes_A_Clostridia_A_Christensenellales_CAG-74_UBA11524_UBA11524 sp000437595_MB2bin287	Functional set_ Environmental information processing_ Drug resistance_Aminoglycoside resistance, protease FtsH	0,002	0,039	0,474
Bacteria_Actinobacteriota_Coriobacteriia_Coriobacteriales_Eggerthellaceae_CAG-1427_?_MB2bin289	Functional set_ Environmental information processing_ Drug resistance_Aminoglycoside resistance, protease FtsH	0,001	0,024	0,514
Bacteria_Firmicutes_A_Clostridia_Lachnospirales_Lachnospiraceae_Coprococcus_A_Coprococcus_A catus_MB2bin38	Functional set_ Environmental information processing_ Drug resistance_Aminoglycoside resistance, protease FtsH	0,014	0,095	0,391
Bacteria_Bacteroidota_Bacteroidia_Bacteroidales_Bacteroidaceae_Phocaeicola_Phocaeicola plebeius_A_MB2bin65	Functional set_ Environmental information processing_ Drug resistance_Aminoglycoside resistance, protease FtsH	0,004	0,053	0,449

Bacteria_Firmicutes_A_Clostridia _Lachnospirales_Lachnospiraceae _Blautia_A_Blautia_A sp900548245_MB2bin70	Functional set_ Environmental information processing_ Drug resistance_Aminoglycoside resistance, protease FtsH	0,015	0,100	0,387
Bacteria_Firmicutes_A_Clostridia _Oscillospirales_Butyricicoccacea e_Agathobaculum_Agathobaculu m butyriciproducens_MB2bin73	Functional set_ Environmental information processing_ Drug resistance_Aminoglycoside resistance, protease FtsH	0,000	0,018	0,560
Bacteria_Firmicutes_A_Clostridia _Lachnospirales_Lachnospiraceae _Blautia_A_Blautia_A massiliensis_MB2bin79	Functional set_ Environmental information processing_ Drug resistance_Aminoglycoside resistance, protease FtsH	0,008	0,068	0,421
Bacteria_Firmicutes_A_Clostridia _Lachnospirales_Lachnospirales_ Dorea_Dorea longicatena_MB2bin81	Functional set_ Environmental information processing_ Drug resistance_Aminoglycoside resistance, protease FtsH	0,000	0,000	0,674
Bacteria_Firmicutes_A_Clostridia _Lachnospirales_Lachnospiraceae _Ruminococcus_A_Ruminococcus _A sp003011855_MB2bin82	Functional set_ Environmental information processing_ Drug resistance_Aminoglycoside resistance, protease FtsH	0,009	0,073	0,411
Bacteria_Firmicutes_C_Negativic utes_Veillonellales_Dialisteracea e_Dialister_Dialister invisus_specI_v3_Cluster3691	Functional set_ Environmental information processing_ Drug resistance_Aminoglycoside resistance, protease FtsH	0,008	0,069	-0,418
Bacteria_Bacteroidota_Bacteroidi a_Bacteroidales_Bacteroidaceae_ Bacteroides_Bacteroides ovatus_MB2bin110	Pathway module_ Nucleotide and amino acid metabolism_ Pyrimidine metabolism_Uridine monophosphate biosynthesis, glutamine (+ PRPP) => UMP	0,002	0,056	-0,481
Bacteria_Actinobacteriota_Actino mycetia_Actinomycetales_Bifido bacteriaceae_Bifidobacterium_Bif idobacterium adolescentis_MB2bin135	Pathway module_ Nucleotide and amino acid metabolism_ Pyrimidine metabolism_Uridine monophosphate biosynthesis, glutamine (+ PRPP) => UMP	0,000	0,000	0,699
Bacteria_Actinobacteriota_Actino mycetia_Actinomycetales_Bifido bacteriaceae_Bifidobacterium_Bif idobacterium pseudocatenulatum_MB2bin265	Pathway module_ Nucleotide and amino acid metabolism_ Pyrimidine metabolism_Uridine monophosphate biosynthesis, glutamine (+ PRPP) => UMP	0,001	0,047	0,495
Bacteria_Firmicutes_A_Clostridia _A_Christensenellales_QAND01_ UMGS1975_UMGS1975 sp900546685_MB2bin272	Pathway module_ Nucleotide and amino acid metabolism_ Pyrimidine metabolism_Uridine monophosphate biosynthesis, glutamine (+ PRPP) => UMP	0,000	0,001	0,628
Bacteria_Actinobacteriota_Actino mycetia_Actinomycetales_Bifido bacteriaceae_Bifidobacterium_Bif idobacterium angulatum_MB2bin288	Pathway module_ Nucleotide and amino acid metabolism_ Pyrimidine metabolism_Uridine monophosphate biosynthesis, glutamine (+ PRPP) => UMP	0,000	0,002	0,598
Bacteria_Bacteroidota_Bacteroidi a_Bacteroidales_Rikenellaceae_A listipes_Alistipes	Pathway module_ Nucleotide and amino acid metabolism_ Pyrimidine metabolism_Uridine	0,004	0,094	-0,452

sp900083545_specI_v3_Cluster1016	monophosphate biosynthesis, glutamine (+ PRPP) => UMP			
Bacteria_Actinobacteriota_Actinobacteria_Actinomycetales_Bifidobacteriaceae_Bifidobacterium_Bifidobacterium ruminantium_specI_v3_Cluster2702	Pathway module_ Nucleotide and amino acid metabolism_ Pyrimidine metabolism_Uridine monophosphate biosynthesis, glutamine (+ PRPP) => UMP	0,000	0,000	0,669
Bacteria_Bacteroidota_Bacteroidia_Bacteroidales_Rikenellaceae_Alistipes_Alistipes shahii_MB2bin10	Pathway module_ Nucleotide and amino acid metabolism_ Cofactor and vitamin biosynthesis_Heme biosynthesis, glutamate => protoheme/siroheme	0,009	0,077	-0,416
Bacteria_Firmicutes_A_Clostridia_Oscillospirales_Oscillospiraceae_CAG-83_?_MB2bin100	Pathway module_ Nucleotide and amino acid metabolism_ Cofactor and vitamin biosynthesis_Heme biosynthesis, glutamate => protoheme/siroheme	0,008	0,077	-0,417
Bacteria_Firmicutes_A_Clostridia_Oscillospirales_Oscillospiraceae_CAG-103_?_MB2bin101	Pathway module_ Nucleotide and amino acid metabolism_ Cofactor and vitamin biosynthesis_Heme biosynthesis, glutamate => protoheme/siroheme	0,000	0,011	-0,538
Bacteria_Firmicutes_A_Clostridia_Lachnospirales_Lachnospiraceae_Eubacterium_I_Eubacterium_I ramulus_MB2bin11	Pathway module_ Nucleotide and amino acid metabolism_ Cofactor and vitamin biosynthesis_Heme biosynthesis, glutamate => protoheme/siroheme	0,009	0,077	0,413
Bacteria_Firmicutes_A_Clostridia_Lachnospirales_Lachnospiraceae_Blautia_A_?_MB2bin128	Pathway module_ Nucleotide and amino acid metabolism_ Cofactor and vitamin biosynthesis_Heme biosynthesis, glutamate => protoheme/siroheme	0,002	0,028	0,474
Bacteria_Firmicutes_A_Clostridia_Lachnospirales_Lachnospiraceae_Blautia_A_?_MB2bin14	Pathway module_ Nucleotide and amino acid metabolism_ Cofactor and vitamin biosynthesis_Heme biosynthesis, glutamate => protoheme/siroheme	0,001	0,023	0,500
Bacteria_Firmicutes_A_Clostridia_Oscillospirales_Oscillospiraceae_CAG-170_CAG-170 sp003516765_MB2bin143	Pathway module_ Nucleotide and amino acid metabolism_ Cofactor and vitamin biosynthesis_Heme biosynthesis, glutamate => protoheme/siroheme	0,000	0,011	-0,543

Bacteria_Firmicutes_A_Clostridia_Oscillospirales_Acutalibacteraceae_CAG-180_CAG-180 sp000432435_MB2bin173	Pathway module_ Nucleotide and amino acid metabolism_ Cofactor and vitamin biosynthesis_Heme biosynthesis, glutamate => protoheme/siroheme	0,001	0,023	0,495
Bacteria_Firmicutes_A_Clostridia_Lachnospirales_Lachnospiraceae_Eubacterium_I_Eubacterium_I sp900546495_MB2bin174	Pathway module_ Nucleotide and amino acid metabolism_ Cofactor and vitamin biosynthesis_Heme biosynthesis, glutamate => protoheme/siroheme	0,002	0,024	0,487
Bacteria_Firmicutes_A_Clostridia_Lachnospirales_Lachnospiraceae_Blautia_A_?_MB2bin175	Pathway module_ Nucleotide and amino acid metabolism_ Cofactor and vitamin biosynthesis_Heme biosynthesis, glutamate => protoheme/siroheme	0,008	0,077	0,418
Bacteria_Firmicutes_A_Clostridia_Oscillospirales_Oscillospiraceae_CAG-83_?_MB2bin179	Pathway module_ Nucleotide and amino acid metabolism_ Cofactor and vitamin biosynthesis_Heme biosynthesis, glutamate => protoheme/siroheme	0,009	0,077	-0,411
Bacteria_Bacteroidota_Bacteroidia_Bacteroidales_Tannerellaceae_Parabacteroides_Parabacteroides johnsonii_MB2bin185	Pathway module_ Nucleotide and amino acid metabolism_ Cofactor and vitamin biosynthesis_Heme biosynthesis, glutamate => protoheme/siroheme	0,002	0,024	-0,490
Bacteria_Actinobacteriota_Coriobacteriia_Coriobacteriales_Eggerthellaceae_Adlercreutzia_Adlercreutzia celatus_A_MB2bin193	Pathway module_ Nucleotide and amino acid metabolism_ Cofactor and vitamin biosynthesis_Heme biosynthesis, glutamate => protoheme/siroheme	0,000	0,009	0,601
Bacteria_Firmicutes_A_Clostridia_Oscillospirales_Oscillospiraceae_ER4_ER4 sp000765235_MB2bin20	Pathway module_ Nucleotide and amino acid metabolism_ Cofactor and vitamin biosynthesis_Heme biosynthesis, glutamate => protoheme/siroheme	0,002	0,026	-0,481
Bacteria_Firmicutes_A_Clostridia_Oscillospirales_Ruminococcaceae_Ruminiclostridium_E_Ruminiclostridium_E sp003512525_MB2bin228	Pathway module_ Nucleotide and amino acid metabolism_ Cofactor and vitamin biosynthesis_Heme biosynthesis, glutamate => protoheme/siroheme	0,000	0,011	-0,563
Bacteria_Firmicutes_A_Clostridia_Oscillospirales_Acutalibacteraceae_Eubacterium_R_Eubacterium_R sp000434995_MB2bin275	Pathway module_ Nucleotide and amino acid metabolism_ Cofactor and vitamin biosynthesis_Heme	0,008	0,077	-0,418

	biosynthesis, glutamate => protoheme/siroheme			
Bacteria_Firmicutes_A_Clostridia_Oscillospirales_Oscillospiraceae_CAG-170_CAG-170 sp900545925_MB2bin33	Pathway module_ Nucleotide and amino acid metabolism_ Cofactor and vitamin biosynthesis_Heme biosynthesis, glutamate => protoheme/siroheme	0,000	0,011	-0,549
Bacteria_Firmicutes_A_Clostridia_Lachnospirales_Lachnospiraceae_UBA11774_UBA11774 sp003507655_MB2bin41	Pathway module_ Nucleotide and amino acid metabolism_ Cofactor and vitamin biosynthesis_Heme biosynthesis, glutamate => protoheme/siroheme	0,012	0,090	-0,398
Bacteria_Firmicutes_A_Clostridia_Oscillospirales_Ruminococcaceae_Faecalibacterium_Faecalibacterium prausnitzii_G_MB2bin45	Pathway module_ Nucleotide and amino acid metabolism_ Cofactor and vitamin biosynthesis_Heme biosynthesis, glutamate => protoheme/siroheme	0,014	0,099	0,391
Bacteria_Bacteroidota_Bacteroidia_Bacteroidales_Tannerellaceae_Parabacteroides_Parabacteroides merdae_MB2bin5	Pathway module_ Nucleotide and amino acid metabolism_ Cofactor and vitamin biosynthesis_Heme biosynthesis, glutamate => protoheme/siroheme	0,000	0,011	-0,535
Bacteria_Firmicutes_A_Clostridia_Oscillospirales_Ruminococcaceae_Ruminiclostridium_E_Ruminiclostridium_E siraeum_MB2bin52	Pathway module_ Nucleotide and amino acid metabolism_ Cofactor and vitamin biosynthesis_Heme biosynthesis, glutamate => protoheme/siroheme	0,001	0,022	-0,506
Bacteria_Firmicutes_A_Clostridia_Lachnospirales_Lachnospiraceae_Mediterraneibacter_Mediterraneibacter torques_MB2bin56	Pathway module_ Nucleotide and amino acid metabolism_ Cofactor and vitamin biosynthesis_Heme biosynthesis, glutamate => protoheme/siroheme	0,000	0,011	0,552
Bacteria_Bacteroidota_Bacteroidia_Bacteroidales_Rikenellaceae_Alistipes_Alistipes sp900083545_specI_v3_Cluster1016	Pathway module_ Nucleotide and amino acid metabolism_ Cofactor and vitamin biosynthesis_Heme biosynthesis, glutamate => protoheme/siroheme	0,004	0,049	-0,447
Bacteria_Firmicutes_I_Bacilli_A_Lactobacillales_Streptococcaceae_Streptococcus_Streptococcus sp000187445_specI_v3_Cluster1349	Pathway module_ Nucleotide and amino acid metabolism_ Cofactor and vitamin biosynthesis_Heme biosynthesis, glutamate => protoheme/siroheme	0,010	0,079	0,407

Bacteria_Actinobacteriota_Actinomycetia_Actinomycetales_Bifidobacteriaceae_Bifidobacterium_Bifidobacterium longum_MB2bin141	Pathway module_ Carbohydrate and lipid metabolism_ Fatty acid metabolism_beta-Oxidation, acyl-CoA synthesis	0,000	0,068	0,539
Bacteria_Actinobacteriota_Actinobacteria_Actinomycetales_Bifidobacteriaceae_Bifidobacterium_Bifidobacterium breve_specI_v3_Cluster1098	Pathway module_ Carbohydrate and lipid metabolism_ Fatty acid metabolism_beta-Oxidation, acyl-CoA synthesis	0,001	0,097	0,502
Bacteria_Actinobacteriota_Actinomycetia_Actinomycetales_Bifidobacteriaceae_Bifidobacterium_Bifidobacterium adolescentis_MB2bin135	Functional set_ Environmental information processing_ Two-component regulatory system_SenX3-RegX3 (phosphate starvation response) two-component regulatory system	0,000	0,000	0,658
Bacteria_Actinobacteriota_Actinomycetia_Actinomycetales_Bifidobacteriaceae_Bifidobacterium_Bifidobacterium longum_MB2bin141	Functional set_ Environmental information processing_ Two-component regulatory system_SenX3-RegX3 (phosphate starvation response) two-component regulatory system	0,000	0,000	0,671
Bacteria_Actinobacteriota_Actinomycetia_Actinomycetales_Bifidobacteriaceae_Bifidobacterium_Bifidobacterium pseudocatenulatum_MB2bin265	Functional set_ Environmental information processing_ Two-component regulatory system_SenX3-RegX3 (phosphate starvation response) two-component regulatory system	0,001	0,019	0,521
Bacteria_Firmicutes_A_Clostridia_A_Christensenellales_QAND01_UMGS1975_UMGS1975 sp900546685_MB2bin272	Functional set_ Environmental information processing_ Two-component regulatory system_SenX3-RegX3 (phosphate starvation response) two-component regulatory system	0,002	0,058	0,473
Bacteria_Actinobacteriota_Actinomycetia_Actinomycetales_Bifidobacteriaceae_Bifidobacterium_Bifidobacterium angulatum_MB2bin288	Functional set_ Environmental information processing_ Two-component regulatory system_SenX3-RegX3 (phosphate starvation response) two-component regulatory system	0,000	0,003	0,593
Bacteria_Actinobacteriota_Actinobacteria_Actinomycetales_Bifidobacteriaceae_Bifidobacterium_Bifidobacterium breve_specI_v3_Cluster1098	Functional set_ Environmental information processing_ Two-component regulatory system_SenX3-RegX3 (phosphate starvation response) two-component regulatory system	0,000	0,003	0,591

Bacteria_Actinobacteriota_Actinobacteria_Actinomycetales_Bifidobacteriaceae_Bifidobacterium_Bifidobacterium ruminantium_specI_v3_Cluster2702	Functional set_ Environmental information processing_ Two-component regulatory system_SenX3-RegX3 (phosphate starvation response) two-component regulatory system	0,000	0,005	0,574

**Correlations in PD KEGG Modules**				
**MGS**	**Pathway/KEGG Module**	**p-Value**	**q-Value**	**corr-coeff**
Bacteria_Firmicutes_A_Clostridia_Lachnospirales_Lachnospiraceae_Bariatricus_Bariatricus comes_MB2bin19	Functional set_ Environmental information processing_ Two-component regulatory system_KdpD-KdpE (potassium transport) two-component regulatory system	0,000	0,015	0,586
Bacteria_Actinobacteriota_Coriobacteriia_Coriobacteriales_Coriobacteriaceae_Collinsella_?_MB2bin75	Functional set_ Environmental information processing_ Two-component regulatory system_LytS-LytR two-component regulatory system	0,003	0,034	0,466
Bacteria_Actinobacteriota_Coriobacteriia_Coriobacteriales_Coriobacteriaceae_Collinsella_Collinsella sp000763055_MB2bin202	Functional set_ Environmental information processing_ Two-component regulatory system_LytS-LytR two-component regulatory system	0,006	0,042	0,435
Bacteria_Bacteroidota_Bacteroidia_Bacteroidales_Bacteroidaceae_Bacteroides_B_Bacteroides_B sartorii_specI_v3_Cluster2366	Functional set_ Environmental information processing_ Two-component regulatory system_LytS-LytR two-component regulatory system	0,001	0,022	-0,511
Bacteria_Bacteroidota_Bacteroidia_Bacteroidales_Bacteroidaceae_Bacteroides_Bacteroides thetaiotaomicron_MB2bin137	Functional set_ Environmental information processing_ Two-component regulatory system_LytS-LytR two-component regulatory system	0,006	0,042	-0,436
Bacteria_Bacteroidota_Bacteroidia_Bacteroidales_Bacteroidaceae_Phocaeicola_Phocaeicola vulgatus_MB2bin4	Functional set_ Environmental information processing_ Two-component regulatory system_LytS-LytR two-component regulatory system	0,002	0,034	-0,472
Bacteria_Bacteroidota_Bacteroidia_Bacteroidales_Rikenellaceae_Alistipes_Alistipes finegoldii_MB2bin46	Functional set_ Environmental information processing_ Two-component regulatory system_LytS-LytR two-component regulatory system	0,009	0,052	-0,412
Bacteria_Bacteroidota_Bacteroidia_Bacteroidales_Rikenellaceae_Alistipes_Alistipes obesi_MB2bin28	Functional set_ Environmental information processing_ Two-component regulatory system_LytS-LytR two-component regulatory system	0,002	0,034	-0,476

Bacteria_Bacteroidota_Bacteroidia_Bacteroidales_Rikenellaceae_Alistipes_Alistipes onderdonkii_MB2bin22	Functional set_ Environmental information processing_ Two-component regulatory system_LytS-LytR two-component regulatory system	0,019	0,092	-0,373
Bacteria_Bacteroidota_Bacteroidia_Bacteroidales_Rikenellaceae_Alistipes_Alistipes shahii_MB2bin10	Functional set_ Environmental information processing_ Two-component regulatory system_LytS-LytR two-component regulatory system	0,008	0,049	-0,417
Bacteria_Bacteroidota_Bacteroidia_Bacteroidales_Rikenellaceae_Alistipes_Alistipes sp900083545_specI_v3_Cluster1016	Functional set_ Environmental information processing_ Two-component regulatory system_LytS-LytR two-component regulatory system	0,012	0,063	-0,399
Bacteria_Firmicutes_A_Clostridia_Lachnospirales_Lachnospiraceae_Anaerobutyricum_Anaerobutyricum hallii_MB2bin3	Functional set_ Environmental information processing_ Two-component regulatory system_LytS-LytR two-component regulatory system	0,013	0,067	0,395
Bacteria_Firmicutes_A_Clostridia_Lachnospirales_Lachnospiraceae_Anaerostipes_Anaerostipes hadrus_A_specI_v3_Cluster856	Functional set_ Environmental information processing_ Two-component regulatory system_LytS-LytR two-component regulatory system	0,007	0,045	0,427
Bacteria_Firmicutes_A_Clostridia_Lachnospirales_Lachnospiraceae_Anaerostipes_Anaerostipes hadrus_MB2bin47	Functional set_ Environmental information processing_ Two-component regulatory system_LytS-LytR two-component regulatory system	0,001	0,022	0,517
Bacteria_Firmicutes_A_Clostridia_Lachnospirales_Lachnospiraceae_Bariatricus_Bariatricus comes_MB2bin19	Functional set_ Environmental information processing_ Two-component regulatory system_LytS-LytR two-component regulatory system	0,003	0,036	0,457
Bacteria_Firmicutes_A_Clostridia_Lachnospirales_Lachnospiraceae_Blautia_A_?_MB2bin175	Functional set_ Environmental information processing_ Two-component regulatory system_LytS-LytR two-component regulatory system	0,010	0,058	0,405
Bacteria_Firmicutes_A_Clostridia_Lachnospirales_Lachnospiraceae_Blautia_A_Blautia_A sp900548245_MB2bin70	Functional set_ Environmental information processing_ Two-component regulatory system_LytS-LytR two-component regulatory system	0,007	0,045	0,425
Bacteria_Firmicutes_A_Clostridia_Lachnospirales_Lachnospiraceae_Eubacterium_I_Eubacterium_I sp900546495_MB2bin174	Functional set_ Environmental information processing_ Two-component regulatory system_LytS-LytR two-component regulatory system	0,003	0,036	0,457

Bacteria_Firmicutes_A_Clostridia_Lachnospirales_Lachnospiraceae_Mediterraneibacter_Mediterraneibacter faecis_MB2bin127	Functional set_ Environmental information processing_ Two-component regulatory system_LytS-LytR two-component regulatory system	0,000	0,001	0,645
Bacteria_Firmicutes_A_Clostridia_Lachnospirales_Lachnospiraceae_Ruminococcus_A_Ruminococcus_A sp003011855_MB2bin82	Functional set_ Environmental information processing_ Two-component regulatory system_LytS-LytR two-component regulatory system	0,005	0,042	0,437
Bacteria_Firmicutes_A_Clostridia_Lachnospirales_Lachnospirales_Dorea_Dorea longicatena_B_specI_v3_Cluster3693	Functional set_ Environmental information processing_ Two-component regulatory system_LytS-LytR two-component regulatory system	0,003	0,034	0,469
Bacteria_Firmicutes_A_Clostridia_Lachnospirales_Lachnospirales_Dorea_Dorea longicatena_MB2bin81	Functional set_ Environmental information processing_ Two-component regulatory system_LytS-LytR two-component regulatory system	0,018	0,087	0,378
Bacteria_Firmicutes_A_Clostridia_Monoglobales_A_UBA1381_CAG-41_?_MB2bin97	Functional set_ Environmental information processing_ Two-component regulatory system_LytS-LytR two-component regulatory system	0,005	0,042	0,441
Bacteria_Firmicutes_A_Clostridia_Oscillospirales_Acutalibacteraceae_Ruminococcus_E_Ruminococcus_E bromii_B_MB2bin205	Functional set_ Environmental information processing_ Two-component regulatory system_LytS-LytR two-component regulatory system	0,020	0,092	0,372
Bacteria_Firmicutes_A_Clostridia_Oscillospirales_Oscillospiraceae_CAG-103_?_MB2bin101	Functional set_ Environmental information processing_ Two-component regulatory system_LytS-LytR two-component regulatory system	0,000	0,006	-0,582
Bacteria_Firmicutes_A_Clostridia_Oscillospirales_Oscillospiraceae_CAG-103_CAG-103 sp000432375_MB2bin162	Functional set_ Environmental information processing_ Two-component regulatory system_LytS-LytR two-component regulatory system	0,006	0,045	-0,428
Bacteria_Firmicutes_A_Clostridia_Oscillospirales_Oscillospiraceae_CAG-170_CAG-170 sp000432135_MB2bin27	Functional set_ Environmental information processing_ Two-component regulatory system_LytS-LytR two-component regulatory system	0,002	0,031	-0,488
Bacteria_Firmicutes_A_Clostridia_Oscillospirales_Oscillospiraceae_CAG-170_CAG-170 sp900545925_MB2bin33	Functional set_ Environmental information processing_ Two-component regulatory system_LytS-LytR two-component regulatory system	0,002	0,034	-0,481

Bacteria_Firmicutes_A_Clostridia_Oscillospirales_Oscillospiraceae_CAG-83_?_MB2bin179	Functional set_ Environmental information processing_ Two-component regulatory system_LytS-LytR two-component regulatory system	0,002	0,031	-0,488
Bacteria_Firmicutes_A_Clostridia_Oscillospirales_Oscillospiraceae_ER4_ER4 sp000765235_MB2bin20	Functional set_ Environmental information processing_ Two-component regulatory system_LytS-LytR two-component regulatory system	0,005	0,042	-0,443
Bacteria_Firmicutes_A_Clostridia_Oscillospirales_Oscillospiraceae_Oscillibacter_?_MB2bin147	Functional set_ Environmental information processing_ Two-component regulatory system_LytS-LytR two-component regulatory system	0,006	0,045	-0,430
Bacteria_Firmicutes_A_Clostridia_Oscillospirales_Ruminococcaceae_Gemmiger_Gemmiger sp900539695_MB2bin39	Functional set_ Environmental information processing_ Two-component regulatory system_LytS-LytR two-component regulatory system	0,000	0,016	0,541
Bacteria_Firmicutes_A_Clostridia_Oscillospirales_Ruminococcaceae_Gemmiger_Gemmiger sp900540595_MB2bin67	Functional set_ Environmental information processing_ Two-component regulatory system_LytS-LytR two-component regulatory system	0,008	0,049	0,418
Bacteria_Firmicutes_A_Clostridia_Peptostreptococcales_Peptostreptococcaceae_Romboutsia_Romboutsia lituseburensis_specI_v3_Cluster6795	Functional set_ Environmental information processing_ Two-component regulatory system_LytS-LytR two-component regulatory system	0,000	0,001	0,630
Bacteria_Firmicutes_C_Negativicutes_Veillonellales_Dialisteraceae_Dialister_Dialister succinatiphilus_specI_v3_Cluster11863	Functional set_ Environmental information processing_ Two-component regulatory system_LytS-LytR two-component regulatory system	0,000	0,016	0,534
Bacteria_Firmicutes_I_Bacilli_A_Lactobacillales_Streptococcaceae_Streptococcus_?_MB2bin209	Functional set_ Environmental information processing_ Two-component regulatory system_LytS-LytR two-component regulatory system	0,004	0,042	0,446
Bacteria_Firmicutes_I_Bacilli_A_Lactobacillales_Streptococcaceae_Streptococcus_Streptococcus sp000187445_specI_v3_Cluster1349	Functional set_ Environmental information processing_ Two-component regulatory system_LytS-LytR two-component regulatory system	0,015	0,077	0,386
Bacteria_Firmicutes_I_Bacilli_A_Lactobacillales_Streptococcaceae_Streptococcus_Streptococcus thermophilus_MB2bin145	Functional set_ Environmental information processing_ Two-component regulatory system_LytS-LytR two-component regulatory system	0,004	0,036	0,455

Bacteria_Actinobacteriota_Actinomycetia_Actinomycetales_Bifidobacteriaceae_Bifidobacterium_Bifidobacterium angulatum_MB2bin288	Functional set_ Environmental information processing_ Two-component regulatory system_PhoR-PhoB (phosphate starvation response) two-component regulatory system	0,002	0,085	-0,474
Bacteria_Bacteroidota_Bacteroidia_Bacteroidales_Bacteroidaceae_Bacteroides_B_Bacteroides_B sartorii_specI_v3_Cluster2366	Functional set_ Environmental information processing_ Two-component regulatory system_PhoR-PhoB (phosphate starvation response) two-component regulatory system	0,003	0,085	0,468
Bacteria_Bacteroidota_Bacteroidia_Bacteroidales_Bacteroidaceae_Bacteroides_Bacteroides thetaiotaomicron_MB2bin137	Functional set_ Environmental information processing_ Two-component regulatory system_PhoR-PhoB (phosphate starvation response) two-component regulatory system	0,001	0,069	0,500
Bacteria_Bacteroidota_Bacteroidia_Bacteroidales_Bacteroidaceae_Phocaeicola_Phocaeicola vulgatus_MB2bin4	Functional set_ Environmental information processing_ Two-component regulatory system_PhoR-PhoB (phosphate starvation response) two-component regulatory system	0,001	0,051	0,525
Bacteria_Bacteroidota_Bacteroidia_Bacteroidales_Barnesiellaceae_Barnesiella_Barnesiella intestinihominis_MB2bin112	Functional set_ Environmental information processing_ Two-component regulatory system_PhoR-PhoB (phosphate starvation response) two-component regulatory system	0,003	0,085	0,463
Bacteria_Bacteroidota_Bacteroidia_Bacteroidales_Rikenellaceae_Alistipes_Alistipes obesi_MB2bin28	Functional set_ Environmental information processing_ Two-component regulatory system_PhoR-PhoB (phosphate starvation response) two-component regulatory system	0,000	0,051	0,535
Bacteria_Bacteroidota_Bacteroidia_Bacteroidales_Bacteroidaceae_Bacteroides_B_Bacteroides_B sartorii_specI_v3_Cluster2366	Pathway module_ Carbohydrate and lipid metabolism_ Central carbohydrate metabolism_Glycolysis (Embden-Meyerhof pathway), glucose => pyruvate	0,000	0,009	0,568
Bacteria_Bacteroidota_Bacteroidia_Bacteroidales_Bacteroidaceae_Bacteroides_Bacteroides ovatus_MB2bin110	Pathway module_ Carbohydrate and lipid metabolism_ Central carbohydrate metabolism_Glycolysis (Embden-Meyerhof pathway), glucose => pyruvate	0,000	0,009	0,583
Bacteria_Bacteroidota_Bacteroidia_Bacteroidales_Bacteroidaceae_Phocaeicola_Phocaeicola vulgatus_MB2bin4	Pathway module_ Carbohydrate and lipid metabolism_ Central carbohydrate metabolism_Glycolysis	0,000	0,009	0,590

	(Embden-Meyerhof pathway), glucose => pyruvate			
Bacteria_Actinobacteriota_Actinobacteria_Actinomycetales_Bifidobacteriaceae_Bifidobacterium_Bifidobacterium ruminantium_specI_v3_Cluster2702	Pathway module_ Carbohydrate and lipid metabolism_ Central carbohydrate metabolism_PRPP biosynthesis, ribose 5P => PRPP	0,000	0,000	0,760
Bacteria_Actinobacteriota_Actinomycetia_Actinomycetales_Bifidobacteriaceae_Bifidobacterium_Bifidobacterium adolescentis_MB2bin135	Pathway module_ Carbohydrate and lipid metabolism_ Central carbohydrate metabolism_PRPP biosynthesis, ribose 5P => PRPP	0,000	0,000	0,743
Bacteria_Actinobacteriota_Actinomycetia_Actinomycetales_Bifidobacteriaceae_Bifidobacterium_Bifidobacterium angulatum_MB2bin288	Pathway module_ Carbohydrate and lipid metabolism_ Central carbohydrate metabolism_PRPP biosynthesis, ribose 5P => PRPP	0,000	0,000	0,680
Bacteria_Actinobacteriota_Coriobacteriia_Coriobacteriales_Coriobacteriaceae_Collinsella_?_MB2bin75	Pathway module_ Carbohydrate and lipid metabolism_ Central carbohydrate metabolism_PRPP biosynthesis, ribose 5P => PRPP	0,001	0,009	0,519
Bacteria_Actinobacteriota_Coriobacteriia_Coriobacteriales_Coriobacteriaceae_Collinsella_Collinsella aerofaciens_F_specI_v3_Cluster3625	Pathway module_ Carbohydrate and lipid metabolism_ Central carbohydrate metabolism_PRPP biosynthesis, ribose 5P => PRPP	0,018	0,067	0,378
Bacteria_Actinobacteriota_Coriobacteriia_Coriobacteriales_Coriobacteriaceae_Collinsella_Collinsella sp000763055_MB2bin202	Pathway module_ Carbohydrate and lipid metabolism_ Central carbohydrate metabolism_PRPP biosynthesis, ribose 5P => PRPP	0,000	0,004	0,566
Bacteria_Bacteroidota_Bacteroidia_Bacteroidales_Bacteroidaceae_Bacteroides_B_Bacteroides_B sartorii_specI_v3_Cluster2366	Pathway module_ Carbohydrate and lipid metabolism_ Central carbohydrate metabolism_PRPP biosynthesis, ribose 5P => PRPP	0,001	0,009	-0,517
Bacteria_Bacteroidota_Bacteroidia_Bacteroidales_Bacteroidaceae_Bacteroides_Bacteroides eggerthii_MB2bin146	Pathway module_ Carbohydrate and lipid metabolism_ Central carbohydrate metabolism_PRPP biosynthesis, ribose 5P => PRPP	0,000	0,003	-0,590
Bacteria_Bacteroidota_Bacteroidia_Bacteroidales_Bacteroidaceae_Bacteroides_Bacteroides fragilis_MB2bin178	Pathway module_ Carbohydrate and lipid metabolism_ Central carbohydrate metabolism_PRPP biosynthesis, ribose 5P => PRPP	0,000	0,005	-0,559
Bacteria_Bacteroidota_Bacteroidia_Bacteroidales_Bacteroidaceae_Bacteroides_Bacteroides ovatus_MB2bin110	Pathway module_ Carbohydrate and lipid metabolism_ Central carbohydrate metabolism_PRPP biosynthesis, ribose 5P => PRPP	0,000	0,004	-0,573
Bacteria_Bacteroidota_Bacteroidia_Bacteroidales_Bacteroidaceae_	Pathway module_ Carbohydrate and lipid metabolism_ Central	0,001	0,010	-0,505

Bacteroides_Bacteroides stercoris_MB2bin42	carbohydrate metabolism_PRPP biosynthesis, ribose 5P => PRPP			
Bacteria_Bacteroidota_Bacteroidia_Bacteroidales_Bacteroidaceae_Bacteroides_Bacteroides thetaiotaomicron_MB2bin137	Pathway module_ Carbohydrate and lipid metabolism_ Central carbohydrate metabolism_PRPP biosynthesis, ribose 5P => PRPP	0,002	0,012	-0,491
Bacteria_Bacteroidota_Bacteroidia_Bacteroidales_Bacteroidaceae_Bacteroides_Bacteroides uniformis_MB2bin1	Pathway module_ Carbohydrate and lipid metabolism_ Central carbohydrate metabolism_PRPP biosynthesis, ribose 5P => PRPP	0,001	0,011	-0,501
Bacteria_Bacteroidota_Bacteroidia_Bacteroidales_Bacteroidaceae_Phocaeicola_Phocaeicola vulgatus_MB2bin4	Pathway module_ Carbohydrate and lipid metabolism_ Central carbohydrate metabolism_PRPP biosynthesis, ribose 5P => PRPP	0,001	0,009	-0,521
Bacteria_Bacteroidota_Bacteroidia_Bacteroidales_Bacteroidaceae_Prevotella_?_MB2bin51	Pathway module_ Carbohydrate and lipid metabolism_ Central carbohydrate metabolism_PRPP biosynthesis, ribose 5P => PRPP	0,010	0,047	0,409
Bacteria_Bacteroidota_Bacteroidia_Bacteroidales_Bacteroidaceae_Prevotella_Prevotella copri_A_MB2bin109	Pathway module_ Carbohydrate and lipid metabolism_ Central carbohydrate metabolism_PRPP biosynthesis, ribose 5P => PRPP	0,001	0,011	0,496
Bacteria_Bacteroidota_Bacteroidia_Bacteroidales_Barnesiellaceae_Barnesiella_Barnesiella intestinihominis_MB2bin112	Pathway module_ Carbohydrate and lipid metabolism_ Central carbohydrate metabolism_PRPP biosynthesis, ribose 5P => PRPP	0,006	0,036	-0,433
Bacteria_Bacteroidota_Bacteroidia_Bacteroidales_Rikenellaceae_Alistipes_Alistipes finegoldii_MB2bin46	Pathway module_ Carbohydrate and lipid metabolism_ Central carbohydrate metabolism_PRPP biosynthesis, ribose 5P => PRPP	0,005	0,034	-0,439
Bacteria_Bacteroidota_Bacteroidia_Bacteroidales_Rikenellaceae_Alistipes_Alistipes obesi_MB2bin28	Pathway module_ Carbohydrate and lipid metabolism_ Central carbohydrate metabolism_PRPP biosynthesis, ribose 5P => PRPP	0,022	0,082	-0,366
Bacteria_Bacteroidota_Bacteroidia_Bacteroidales_Rikenellaceae_Alistipes_Alistipes shahii_MB2bin10	Pathway module_ Carbohydrate and lipid metabolism_ Central carbohydrate metabolism_PRPP biosynthesis, ribose 5P => PRPP	0,004	0,026	-0,455
Bacteria_Bacteroidota_Bacteroidia_Bacteroidales_Rikenellaceae_Alistipes_Alistipes sp900083545_specI_v3_Cluster1016	Pathway module_ Carbohydrate and lipid metabolism_ Central carbohydrate metabolism_PRPP biosynthesis, ribose 5P => PRPP	0,016	0,065	-0,382
Bacteria_Bacteroidota_Bacteroidia_Bacteroidales_Tannerellaceae_Parabacteroides_Parabacteroides johnsonii_MB2bin185	Pathway module_ Carbohydrate and lipid metabolism_ Central carbohydrate metabolism_PRPP biosynthesis, ribose 5P => PRPP	0,008	0,042	-0,417
Bacteria_Bacteroidota_Bacteroidia_Bacteroidales_Tannerellaceae_Parabacteroides_Parabacteroides merdae_MB2bin5	Pathway module_ Carbohydrate and lipid metabolism_ Central carbohydrate metabolism_PRPP biosynthesis, ribose 5P => PRPP	0,028	0,096	-0,352

Bacteria_Firmicutes_A_Clostridia_A_Christensenellales_CAG-74_SFFH01_SFFH01 sp900542445_MB2bin113	Pathway module_ Carbohydrate and lipid metabolism_ Central carbohydrate metabolism_PRPP biosynthesis, ribose 5P => PRPP	0,012	0,053	0,398
Bacteria_Firmicutes_A_Clostridia_A_Christensenellales_QAND01_UMGS1975_UMGS1975 sp900546685_MB2bin272	Pathway module_ Carbohydrate and lipid metabolism_ Central carbohydrate metabolism_PRPP biosynthesis, ribose 5P => PRPP	0,000	0,009	0,534
Bacteria_Firmicutes_A_Clostridia_Lachnospirales_Lachnospiraceae_Anaerobutyricum_?_MB2bin124	Pathway module_ Carbohydrate and lipid metabolism_ Central carbohydrate metabolism_PRPP biosynthesis, ribose 5P => PRPP	0,001	0,009	0,520
Bacteria_Firmicutes_A_Clostridia_Lachnospirales_Lachnospiraceae_Anaerobutyricum_Anaerobutyricum hallii_MB2bin3	Pathway module_ Carbohydrate and lipid metabolism_ Central carbohydrate metabolism_PRPP biosynthesis, ribose 5P => PRPP	0,002	0,014	0,483
Bacteria_Firmicutes_A_Clostridia_Lachnospirales_Lachnospiraceae_Anaerostipes_Anaerostipes hadrus_A_specI_v3_Cluster856	Pathway module_ Carbohydrate and lipid metabolism_ Central carbohydrate metabolism_PRPP biosynthesis, ribose 5P => PRPP	0,006	0,037	0,431
Bacteria_Firmicutes_A_Clostridia_Lachnospirales_Lachnospiraceae_Blautia_A_?_MB2bin14	Pathway module_ Carbohydrate and lipid metabolism_ Central carbohydrate metabolism_PRPP biosynthesis, ribose 5P => PRPP	0,007	0,039	0,426
Bacteria_Firmicutes_A_Clostridia_Lachnospirales_Lachnospiraceae_Blautia_A_?_MB2bin175	Pathway module_ Carbohydrate and lipid metabolism_ Central carbohydrate metabolism_PRPP biosynthesis, ribose 5P => PRPP	0,004	0,026	0,454
Bacteria_Firmicutes_A_Clostridia_Lachnospirales_Lachnospiraceae_Blautia_A_?_MB2bin9	Pathway module_ Carbohydrate and lipid metabolism_ Central carbohydrate metabolism_PRPP biosynthesis, ribose 5P => PRPP	0,009	0,044	-0,413
Bacteria_Firmicutes_A_Clostridia_Lachnospirales_Lachnospiraceae_Blautia_A_Blautia_A massiliensis_MB2bin79	Pathway module_ Carbohydrate and lipid metabolism_ Central carbohydrate metabolism_PRPP biosynthesis, ribose 5P => PRPP	0,001	0,009	0,526
Bacteria_Firmicutes_A_Clostridia_Lachnospirales_Lachnospiraceae_Blautia_A_Blautia_A sp900066145_MB2bin111	Pathway module_ Carbohydrate and lipid metabolism_ Central carbohydrate metabolism_PRPP biosynthesis, ribose 5P => PRPP	0,008	0,039	0,421
Bacteria_Firmicutes_A_Clostridia_Lachnospirales_Lachnospiraceae_Blautia_A_Blautia_A sp900066165_MB2bin2	Pathway module_ Carbohydrate and lipid metabolism_ Central carbohydrate metabolism_PRPP biosynthesis, ribose 5P => PRPP	0,014	0,059	0,390
Bacteria_Firmicutes_A_Clostridia_Lachnospirales_Lachnospiraceae_Blautia_A_Blautia_A sp900066335_MB2bin25	Pathway module_ Carbohydrate and lipid metabolism_ Central carbohydrate metabolism_PRPP biosynthesis, ribose 5P => PRPP	0,001	0,011	0,496
Bacteria_Firmicutes_A_Clostridia_Lachnospirales_Lachnospiraceae_Blautia_A_Blautia_A sp900066355_MGS00045	Pathway module_ Carbohydrate and lipid metabolism_ Central carbohydrate metabolism_PRPP biosynthesis, ribose 5P => PRPP	0,010	0,047	0,407

Bacteria_Firmicutes_A_Clostridia_Lachnospirales_Lachnospiraceae_Coprococcus_A_Coprococcus_A catus_MB2bin38	Pathway module_ Carbohydrate and lipid metabolism_ Central carbohydrate metabolism_PRPP biosynthesis, ribose 5P => PRPP	0,017	0,067	0,379
Bacteria_Firmicutes_A_Clostridia_Lachnospirales_Lachnospiraceae_Coprococcus_Coprococcus eutactus_A_MB2bin7	Pathway module_ Carbohydrate and lipid metabolism_ Central carbohydrate metabolism_PRPP biosynthesis, ribose 5P => PRPP	0,011	0,050	0,402
Bacteria_Firmicutes_A_Clostridia_Lachnospirales_Lachnospiraceae_Coprococcus_Coprococcus eutactus_MB2bin153	Pathway module_ Carbohydrate and lipid metabolism_ Central carbohydrate metabolism_PRPP biosynthesis, ribose 5P => PRPP	0,008	0,039	0,422
Bacteria_Firmicutes_A_Clostridia_Lachnospirales_Lachnospiraceae_Mediterraneibacter_Mediterraneibacter faecis_MB2bin127	Pathway module_ Carbohydrate and lipid metabolism_ Central carbohydrate metabolism_PRPP biosynthesis, ribose 5P => PRPP	0,001	0,009	0,528
Bacteria_Firmicutes_A_Clostridia_Lachnospirales_Lachnospiraceae_Ruminococcus_A_Ruminococcus_A sp000437095_MB2bin155	Pathway module_ Carbohydrate and lipid metabolism_ Central carbohydrate metabolism_PRPP biosynthesis, ribose 5P => PRPP	0,001	0,010	0,509
Bacteria_Firmicutes_A_Clostridia_Lachnospirales_Lachnospiraceae_Ruminococcus_A_Ruminococcus_A sp003011855_MB2bin82	Pathway module_ Carbohydrate and lipid metabolism_ Central carbohydrate metabolism_PRPP biosynthesis, ribose 5P => PRPP	0,000	0,001	0,626
Bacteria_Firmicutes_A_Clostridia_Lachnospirales_Lachnospiraceae_UMGS1375_UMGS1375 sp900066615_MB2bin12	Pathway module_ Carbohydrate and lipid metabolism_ Central carbohydrate metabolism_PRPP biosynthesis, ribose 5P => PRPP	0,006	0,036	0,435
Bacteria_Firmicutes_A_Clostridia_Lachnospirales_Lachnospirales_Dorea_Dorea formicigenerans_MB2bin120	Pathway module_ Carbohydrate and lipid metabolism_ Central carbohydrate metabolism_PRPP biosynthesis, ribose 5P => PRPP	0,001	0,010	0,503
Bacteria_Firmicutes_A_Clostridia_Oscillospirales_Ruminococcaceae_Gemmiger_Gemmiger sp900539695_MB2bin39	Pathway module_ Carbohydrate and lipid metabolism_ Central carbohydrate metabolism_PRPP biosynthesis, ribose 5P => PRPP	0,015	0,059	0,389
Bacteria_Firmicutes_A_Clostridia_Oscillospirales_Ruminococcaceae_Gemmiger_Gemmiger sp900540595_MB2bin67	Pathway module_ Carbohydrate and lipid metabolism_ Central carbohydrate metabolism_PRPP biosynthesis, ribose 5P => PRPP	0,025	0,091	0,358
Bacteria_Firmicutes_A_Clostridia_Oscillospirales_Ruminococcaceae_Ruminococcus_C_Ruminococcus_C sp000433635_MB2bin321	Pathway module_ Carbohydrate and lipid metabolism_ Central carbohydrate metabolism_PRPP biosynthesis, ribose 5P => PRPP	0,026	0,091	0,357
Bacteria_Firmicutes_A_Clostridia_Peptostreptococcales_Peptostreptococcaceae_Romboutsia_Romboutsia lituseburensis_specI_v3_Cluster6795	Pathway module_ Carbohydrate and lipid metabolism_ Central carbohydrate metabolism_PRPP biosynthesis, ribose 5P => PRPP	0,028	0,096	0,351

Bacteria_Firmicutes_C_Negativicutes_Veillonellales_Dialisteraceae_Dialister_Dialister succinatiphilus_specI_v3_Cluster 11863	Pathway module_ Carbohydrate and lipid metabolism_ Central carbohydrate metabolism_PRPP biosynthesis, ribose 5P => PRPP	0,024	0,089	0,360
Bacteria_Firmicutes_I_Bacilli_A_Erysipelotrichales_Erysipelotrichaceae_Holdemanella_Holdemanella sp002299315_MB2bin140	Pathway module_ Carbohydrate and lipid metabolism_ Central carbohydrate metabolism_PRPP biosynthesis, ribose 5P => PRPP	0,013	0,057	0,394
Bacteria_Firmicutes_I_Bacilli_A_Lactobacillales_Streptococcaceae_Streptococcus_?_MB2bin209	Pathway module_ Carbohydrate and lipid metabolism_ Central carbohydrate metabolism_PRPP biosynthesis, ribose 5P => PRPP	0,007	0,039	0,423
Archaea_Methanobacteriota_Methanobacteria_Methanobacteriales_Methanobacteriaceae_Methanobrevibacter_A_Methanobrevibacter_A smithii_MB2bin267	Pathway module_ Carbohydrate and lipid metabolism_ Central carbohydrate metabolism_Semi-phosphorylative Entner-Doudoroff pathway, gluconate => glycerate-3P	0,012	0,081	-0,397
Bacteria_Actinobacteriota_Coriobacteriia_Coriobacteriales_Eggerthellaceae_Adlercreutzia_Adlercreutzia celatus_A_MB2bin193	Pathway module_ Carbohydrate and lipid metabolism_ Central carbohydrate metabolism_Semi-phosphorylative Entner-Doudoroff pathway, gluconate => glycerate-3P	0,014	0,087	-0,392
Bacteria_Bacteroidota_Bacteroidia_Bacteroidales_Bacteroidaceae_Paraprevotella_Paraprevotella clara_MB2bin115	Pathway module_ Carbohydrate and lipid metabolism_ Central carbohydrate metabolism_Semi-phosphorylative Entner-Doudoroff pathway, gluconate => glycerate-3P	0,015	0,091	0,387
Bacteria_Bacteroidota_Bacteroidia_Bacteroidales_Bacteroidaceae_Prevotella_Prevotella copri_A_MB2bin109	Pathway module_ Carbohydrate and lipid metabolism_ Central carbohydrate metabolism_Semi-phosphorylative Entner-Doudoroff pathway, gluconate => glycerate-3P	0,017	0,095	0,380
Bacteria_Bacteroidota_Bacteroidia_Bacteroidales_Muribaculaceae_CAG-279_CAG-279 sp000437795_MB2bin226	Pathway module_ Carbohydrate and lipid metabolism_ Central carbohydrate metabolism_Semi-phosphorylative Entner-Doudoroff pathway, gluconate => glycerate-3P	0,000	0,002	0,596
Bacteria_Bacteroidota_Bacteroidia_Bacteroidales_Rikenellaceae_Tidjanibacter_Tidjanibacter inops_MB2bin306	Pathway module_ Carbohydrate and lipid metabolism_ Central carbohydrate metabolism_Semi-	0,002	0,030	-0,485

	phosphorylative Entner-Doudoroff pathway, gluconate => glycerate-3P			
Bacteria_Firmicutes_A_Clostridia_A_Christensenellales_CAG-314_CAG-314_CAG-314 sp000437915_MB2bin422	Pathway module_ Carbohydrate and lipid metabolism_ Central carbohydrate metabolism_Semi-phosphorylative Entner-Doudoroff pathway, gluconate => glycerate-3P	0,005	0,050	-0,445
Bacteria_Firmicutes_A_Clostridia_Lachnospirales_Lachnospiraceae_Acetatifactor_Acetatifactor sp900066365_MB2bin8	Pathway module_ Carbohydrate and lipid metabolism_ Central carbohydrate metabolism_Semi-phosphorylative Entner-Doudoroff pathway, gluconate => glycerate-3P	0,006	0,052	0,435
Bacteria_Firmicutes_A_Clostridia_Lachnospirales_Lachnospiraceae_Acetatifactor_Acetatifactor sp900066565_MB2bin18	Pathway module_ Carbohydrate and lipid metabolism_ Central carbohydrate metabolism_Semi-phosphorylative Entner-Doudoroff pathway, gluconate => glycerate-3P	0,010	0,071	0,406
Bacteria_Firmicutes_A_Clostridia_Lachnospirales_Lachnospiraceae_CAG-127_CAG-127 sp900319515_MB2bin98	Pathway module_ Carbohydrate and lipid metabolism_ Central carbohydrate metabolism_Semi-phosphorylative Entner-Doudoroff pathway, gluconate => glycerate-3P	0,000	0,005	0,555
Bacteria_Firmicutes_A_Clostridia_Lachnospirales_Lachnospiraceae_CAG-45_CAG-45 sp000438375_MB2bin229	Pathway module_ Carbohydrate and lipid metabolism_ Central carbohydrate metabolism_Semi-phosphorylative Entner-Doudoroff pathway, gluconate => glycerate-3P	0,006	0,052	0,430
Bacteria_Firmicutes_A_Clostridia_Lachnospirales_Lachnospiraceae_Coprococcus_A_Coprococcus_A catus_MB2bin38	Pathway module_ Carbohydrate and lipid metabolism_ Central carbohydrate metabolism_Semi-phosphorylative Entner-Doudoroff pathway, gluconate => glycerate-3P	0,005	0,050	0,438
Bacteria_Firmicutes_A_Clostridia_Lachnospirales_Lachnospiraceae_Coprococcus_Coprococcus eutactus_MB2bin153	Pathway module_ Carbohydrate and lipid metabolism_ Central carbohydrate metabolism_Semi-phosphorylative Entner-	0,005	0,050	0,444

	Doudoroff pathway, gluconate => glycerate-3P			
Bacteria_Firmicutes_A_Clostridia_Lachnospirales_Lachnospiraceae_KLE1615_KLE1615 sp900066985_MB2bin161	Pathway module_ Carbohydrate and lipid metabolism_ Central carbohydrate metabolism_Semi-phosphorylative Entner-Doudoroff pathway, gluconate => glycerate-3P	0,000	0,002	0,603
Bacteria_Firmicutes_A_Clostridia_Oscillospirales_Acutalibacteraceae_Eubacterium_R_Eubacterium_R sp000434995_MB2bin275	Pathway module_ Carbohydrate and lipid metabolism_ Central carbohydrate metabolism_Semi-phosphorylative Entner-Doudoroff pathway, gluconate => glycerate-3P	0,018	0,096	0,377
Bacteria_Firmicutes_A_Clostridia_Oscillospirales_Acutalibacteraceae_Ruminococcus_E_Ruminococcus_E bromii_B_MB2bin205	Pathway module_ Carbohydrate and lipid metabolism_ Central carbohydrate metabolism_Semi-phosphorylative Entner-Doudoroff pathway, gluconate => glycerate-3P	0,005	0,050	-0,440
Bacteria_Firmicutes_A_Clostridia_Oscillospirales_Butyricicoccaceae_Agathobaculum_Agathobaculum sp003481705_MB2bin186	Pathway module_ Carbohydrate and lipid metabolism_ Central carbohydrate metabolism_Semi-phosphorylative Entner-Doudoroff pathway, gluconate => glycerate-3P	0,004	0,048	0,454
Bacteria_Firmicutes_A_Clostridia_Oscillospirales_Oscillospiraceae_CAG-170_CAG-170 sp000432135_MB2bin27	Pathway module_ Carbohydrate and lipid metabolism_ Central carbohydrate metabolism_Semi-phosphorylative Entner-Doudoroff pathway, gluconate => glycerate-3P	0,006	0,052	0,429
Bacteria_Firmicutes_A_Clostridia_Oscillospirales_Oscillospiraceae_CAG-170_CAG-170 sp003516765_MB2bin143	Pathway module_ Carbohydrate and lipid metabolism_ Central carbohydrate metabolism_Semi-phosphorylative Entner-Doudoroff pathway, gluconate => glycerate-3P	0,005	0,050	0,442
Bacteria_Firmicutes_A_Clostridia_Oscillospirales_Oscillospiraceae_CAG-170_CAG-170 sp900545925_MB2bin33	Pathway module_ Carbohydrate and lipid metabolism_ Central carbohydrate metabolism_Semi-phosphorylative Entner-	0,010	0,071	0,406

	Doudoroff pathway, gluconate => glycerate-3P			
Bacteria_Firmicutes_A_Clostridia_Oscillospirales_Oscillospiraceae_CAG-83_?_MB2bin100	Pathway module_ Carbohydrate and lipid metabolism_ Central carbohydrate metabolism_Semi-phosphorylative Entner-Doudoroff pathway, gluconate => glycerate-3P	0,002	0,034	0,476
Bacteria_Firmicutes_A_Clostridia_Oscillospirales_Oscillospiraceae_CAG-83_?_MB2bin179	Pathway module_ Carbohydrate and lipid metabolism_ Central carbohydrate metabolism_Semi-phosphorylative Entner-Doudoroff pathway, gluconate => glycerate-3P	0,009	0,067	0,413
Bacteria_Firmicutes_A_Clostridia_Oscillospirales_Ruminococcaceae_Faecalibacterium_Faecalibacterium prausnitzii_C_MB2bin90	Pathway module_ Carbohydrate and lipid metabolism_ Central carbohydrate metabolism_Semi-phosphorylative Entner-Doudoroff pathway, gluconate => glycerate-3P	0,015	0,091	0,386
Bacteria_Firmicutes_A_Clostridia_Oscillospirales_Ruminococcaceae_Faecalibacterium_Faecalibacterium prausnitzii_I_MB2bin63	Pathway module_ Carbohydrate and lipid metabolism_ Central carbohydrate metabolism_Semi-phosphorylative Entner-Doudoroff pathway, gluconate => glycerate-3P	0,000	0,001	0,638
Bacteria_Firmicutes_A_Clostridia_Oscillospirales_Ruminococcaceae_Faecalibacterium_Faecalibacterium sp900539945_MB2bin59	Pathway module_ Carbohydrate and lipid metabolism_ Central carbohydrate metabolism_Semi-phosphorylative Entner-Doudoroff pathway, gluconate => glycerate-3P	0,000	0,000	0,673
Bacteria_Firmicutes_A_Clostridia_Oscillospirales_Ruminococcaceae_Ruminococcus_D_?_MB2bin60	Pathway module_ Carbohydrate and lipid metabolism_ Central carbohydrate metabolism_Semi-phosphorylative Entner-Doudoroff pathway, gluconate => glycerate-3P	0,000	0,007	-0,543
Bacteria_Firmicutes_C_Negativicutes_Acidaminococcales_Acidaminococcaceae_Phascolarctobacterium_A_?_MB2bin381	Pathway module_ Carbohydrate and lipid metabolism_ Central carbohydrate metabolism_Semi-phosphorylative Entner-	0,000	0,002	0,604

	Doudoroff pathway, gluconate => glycerate-3P			
Bacteria_Firmicutes_C_Negativicutes_Veillonellales_Dialisteraceae_Dialister_Dialister invisus_specI_v3_Cluster3691	Pathway module_ Carbohydrate and lipid metabolism_ Central carbohydrate metabolism_Semi-phosphorylative Entner-Doudoroff pathway, gluconate => glycerate-3P	0,000	0,005	-0,559
Bacteria_Firmicutes_I_Bacilli_A_Erysipelotrichales_Erysipelotrichaceae_Absiella_Absiella sp000163515_specI_v3_Cluster2380	Pathway module_ Carbohydrate and lipid metabolism_ Central carbohydrate metabolism_Semi-phosphorylative Entner-Doudoroff pathway, gluconate => glycerate-3P	0,008	0,064	0,417
Bacteria_Firmicutes_I_Bacilli_A_Erysipelotrichales_Erysipelotrichaceae_Holdemanella_Holdemanella sp002299315_MB2bin140	Pathway module_ Carbohydrate and lipid metabolism_ Central carbohydrate metabolism_Semi-phosphorylative Entner-Doudoroff pathway, gluconate => glycerate-3P	0,003	0,047	0,459
Bacteria_Proteobacteria_Gammaproteobacteria_Enterobacterales_Enterobacteriaceae_Klebsiella_B_Klebsiella_B aerogenes_specI_v3_Cluster84	Pathway module_ Carbohydrate and lipid metabolism_ Central carbohydrate metabolism_Semi-phosphorylative Entner-Doudoroff pathway, gluconate => glycerate-3P	0,017	0,095	0,380
Bacteria_Proteobacteria_Gammaproteobacteria_Enterobacterales_Enterobacteriaceae_Klebsiella_Klebsiella variicola_MB2bin427	Pathway module_ Carbohydrate and lipid metabolism_ Central carbohydrate metabolism_Semi-phosphorylative Entner-Doudoroff pathway, gluconate => glycerate-3P	0,000	0,005	0,567
Bacteria_Actinobacteriota_Actinobacteria_Actinomycetales_Bifidobacteriaceae_Bifidobacterium_Bifidobacterium ruminantium_specI_v3_Cluster2702	Pathway module_ Carbohydrate and lipid metabolism_ Sugar metabolism_Trehalose biosynthesis, D-glucose 1P => trehalose	0,003	0,016	0,465
Bacteria_Actinobacteriota_Actinomycetia_Actinomycetales_Bifidobacteriaceae_Bifidobacterium_Bifidobacterium adolescentis_MB2bin135	Pathway module_ Carbohydrate and lipid metabolism_ Sugar metabolism_Trehalose biosynthesis, D-glucose 1P => trehalose	0,001	0,008	0,526

Bacteria_Actinobacteriota_Actinomycetia_Actinomycetales_Bifidobacteriaceae_Bifidobacterium_Bifidobacterium angulatum_MB2bin288	Pathway module_ Carbohydrate and lipid metabolism_ Sugar metabolism_Trehalose biosynthesis, D-glucose 1P => trehalose	0,003	0,018	0,459
Bacteria_Actinobacteriota_Coriobacteriia_Coriobacteriales_Coriobacteriaceae_Collinsella_?_MB2bin75	Pathway module_ Carbohydrate and lipid metabolism_ Sugar metabolism_Trehalose biosynthesis, D-glucose 1P => trehalose	0,034	0,096	0,340
Bacteria_Actinobacteriota_Coriobacteriia_Coriobacteriales_Coriobacteriaceae_Collinsella_Collinsella sp000763055_MB2bin202	Pathway module_ Carbohydrate and lipid metabolism_ Sugar metabolism_Trehalose biosynthesis, D-glucose 1P => trehalose	0,031	0,091	0,345
Bacteria_Bacteroidota_Bacteroidia_Bacteroidales_Bacteroidaceae_Bacteroides_B_Bacteroides_B sartorii_specI_v3_Cluster2366	Pathway module_ Carbohydrate and lipid metabolism_ Sugar metabolism_Trehalose biosynthesis, D-glucose 1P => trehalose	0,000	0,001	-0,630
Bacteria_Bacteroidota_Bacteroidia_Bacteroidales_Bacteroidaceae_Bacteroides_Bacteroides ovatus_MB2bin110	Pathway module_ Carbohydrate and lipid metabolism_ Sugar metabolism_Trehalose biosynthesis, D-glucose 1P => trehalose	0,021	0,070	-0,367
Bacteria_Bacteroidota_Bacteroidia_Bacteroidales_Bacteroidaceae_Bacteroides_Bacteroides stercoris_MB2bin42	Pathway module_ Carbohydrate and lipid metabolism_ Sugar metabolism_Trehalose biosynthesis, D-glucose 1P => trehalose	0,004	0,019	-0,450
Bacteria_Bacteroidota_Bacteroidia_Bacteroidales_Bacteroidaceae_Bacteroides_Bacteroides thetaiotaomicron_MB2bin137	Pathway module_ Carbohydrate and lipid metabolism_ Sugar metabolism_Trehalose biosynthesis, D-glucose 1P => trehalose	0,002	0,015	-0,474
Bacteria_Bacteroidota_Bacteroidia_Bacteroidales_Bacteroidaceae_Phocaeicola_Phocaeicola vulgatus_MB2bin4	Pathway module_ Carbohydrate and lipid metabolism_ Sugar metabolism_Trehalose biosynthesis, D-glucose 1P => trehalose	0,000	0,001	-0,617
Bacteria_Bacteroidota_Bacteroidia_Bacteroidales_Barnesiellaceae_Barnesiella_Barnesiella intestinihominis_MB2bin112	Pathway module_ Carbohydrate and lipid metabolism_ Sugar metabolism_Trehalose biosynthesis, D-glucose 1P => trehalose	0,017	0,059	-0,380
Bacteria_Bacteroidota_Bacteroidia_Bacteroidales_Rikenellaceae_Alistipes_Alistipes finegoldii_MB2bin46	Pathway module_ Carbohydrate and lipid metabolism_ Sugar metabolism_Trehalose biosynthesis, D-glucose 1P => trehalose	0,005	0,022	-0,441

Bacteria_Bacteroidota_Bacteroidia_Bacteroidales_Rikenellaceae_Alistipes_Alistipes obesi_MB2bin28	Pathway module_ Carbohydrate and lipid metabolism_ Sugar metabolism_Trehalose biosynthesis, D-glucose 1P => trehalose	0,000	0,001	-0,612
Bacteria_Bacteroidota_Bacteroidia_Bacteroidales_Rikenellaceae_Alistipes_Alistipes onderdonkii_MB2bin22	Pathway module_ Carbohydrate and lipid metabolism_ Sugar metabolism_Trehalose biosynthesis, D-glucose 1P => trehalose	0,029	0,086	-0,350
Bacteria_Bacteroidota_Bacteroidia_Bacteroidales_Rikenellaceae_Alistipes_Alistipes shahii_MB2bin10	Pathway module_ Carbohydrate and lipid metabolism_ Sugar metabolism_Trehalose biosynthesis, D-glucose 1P => trehalose	0,001	0,008	-0,521
Bacteria_Bacteroidota_Bacteroidia_Bacteroidales_Rikenellaceae_Alistipes_Alistipes sp900083545_specI_v3_Cluster1016	Pathway module_ Carbohydrate and lipid metabolism_ Sugar metabolism_Trehalose biosynthesis, D-glucose 1P => trehalose	0,001	0,009	-0,507
Bacteria_Bacteroidota_Bacteroidia_Bacteroidales_Tannerellaceae_Parabacteroides_Parabacteroides johnsonii_MB2bin185	Pathway module_ Carbohydrate and lipid metabolism_ Sugar metabolism_Trehalose biosynthesis, D-glucose 1P => trehalose	0,000	0,008	-0,542
Bacteria_Bacteroidota_Bacteroidia_Bacteroidales_Tannerellaceae_Parabacteroides_Parabacteroides merdae_MB2bin5	Pathway module_ Carbohydrate and lipid metabolism_ Sugar metabolism_Trehalose biosynthesis, D-glucose 1P => trehalose	0,000	0,006	-0,561
Bacteria_Firmicutes_A_Clostridia_A_Christensenellales_QAND01_UMGS1975_UMGS1975 sp900546685_MB2bin272	Pathway module_ Carbohydrate and lipid metabolism_ Sugar metabolism_Trehalose biosynthesis, D-glucose 1P => trehalose	0,023	0,072	0,363
Bacteria_Firmicutes_A_Clostridia_Lachnospirales_Lachnospiraceae_Agathobacter_Agathobacter faecis_MB2bin13	Pathway module_ Carbohydrate and lipid metabolism_ Sugar metabolism_Trehalose biosynthesis, D-glucose 1P => trehalose	0,009	0,034	0,414
Bacteria_Firmicutes_A_Clostridia_Lachnospirales_Lachnospiraceae_Anaerobutyricum_?_MB2bin124	Pathway module_ Carbohydrate and lipid metabolism_ Sugar metabolism_Trehalose biosynthesis, D-glucose 1P => trehalose	0,001	0,008	0,521
Bacteria_Firmicutes_A_Clostridia_Lachnospirales_Lachnospiraceae_Anaerobutyricum_Anaerobutyricum hallii_MB2bin3	Pathway module_ Carbohydrate and lipid metabolism_ Sugar metabolism_Trehalose biosynthesis, D-glucose 1P => trehalose	0,003	0,016	0,466

Bacteria_Firmicutes_A_Clostridia _Lachnospirales_Lachnospiraceae _Anaerostipes_Anaerostipes hadrus_A_specI_v3_Cluster856	Pathway module_ Carbohydrate and lipid metabolism_ Sugar metabolism_Trehalose biosynthesis, D-glucose 1P => trehalose	0,001	0,008	0,521
Bacteria_Firmicutes_A_Clostridia _Lachnospirales_Lachnospiraceae _Anaerostipes_Anaerostipes hadrus_MB2bin47	Pathway module_ Carbohydrate and lipid metabolism_ Sugar metabolism_Trehalose biosynthesis, D-glucose 1P => trehalose	0,000	0,001	0,633
Bacteria_Firmicutes_A_Clostridia _Lachnospirales_Lachnospiraceae _Bariatricus_Bariatricus comes_MB2bin19	Pathway module_ Carbohydrate and lipid metabolism_ Sugar metabolism_Trehalose biosynthesis, D-glucose 1P => trehalose	0,006	0,025	0,432
Bacteria_Firmicutes_A_Clostridia _Lachnospirales_Lachnospiraceae _Blautia_A_?_MB2bin128	Pathway module_ Carbohydrate and lipid metabolism_ Sugar metabolism_Trehalose biosynthesis, D-glucose 1P => trehalose	0,003	0,016	0,468
Bacteria_Firmicutes_A_Clostridia _Lachnospirales_Lachnospiraceae _Blautia_A_?_MB2bin14	Pathway module_ Carbohydrate and lipid metabolism_ Sugar metabolism_Trehalose biosynthesis, D-glucose 1P => trehalose	0,001	0,009	0,515
Bacteria_Firmicutes_A_Clostridia _Lachnospirales_Lachnospiraceae _Blautia_A_?_MB2bin175	Pathway module_ Carbohydrate and lipid metabolism_ Sugar metabolism_Trehalose biosynthesis, D-glucose 1P => trehalose	0,001	0,009	0,504
Bacteria_Firmicutes_A_Clostridia _Lachnospirales_Lachnospiraceae _Blautia_A_Blautia_A massiliensis_MB2bin79	Pathway module_ Carbohydrate and lipid metabolism_ Sugar metabolism_Trehalose biosynthesis, D-glucose 1P => trehalose	0,002	0,011	0,489
Bacteria_Firmicutes_A_Clostridia _Lachnospirales_Lachnospiraceae _Blautia_A_Blautia_A sp900066145_MB2bin111	Pathway module_ Carbohydrate and lipid metabolism_ Sugar metabolism_Trehalose biosynthesis, D-glucose 1P => trehalose	0,001	0,009	0,509
Bacteria_Firmicutes_A_Clostridia _Lachnospirales_Lachnospiraceae _Blautia_A_Blautia_A sp900066165_MB2bin2	Pathway module_ Carbohydrate and lipid metabolism_ Sugar metabolism_Trehalose biosynthesis, D-glucose 1P => trehalose	0,006	0,025	0,431
Bacteria_Firmicutes_A_Clostridia _Lachnospirales_Lachnospiraceae _Blautia_A_Blautia_A sp900066335_MB2bin25	Pathway module_ Carbohydrate and lipid metabolism_ Sugar metabolism_Trehalose biosynthesis, D-glucose 1P => trehalose	0,001	0,009	0,505

Bacteria_Firmicutes_A_Clostridia _Lachnospirales_Lachnospiraceae _Blautia_A_Blautia_A sp900066355_MGS00045	Pathway module_ Carbohydrate and lipid metabolism_ Sugar metabolism_Trehalose biosynthesis, D-glucose 1P => trehalose	0,006	0,025	0,429
Bacteria_Firmicutes_A_Clostridia _Lachnospirales_Lachnospiraceae _Blautia_A_Blautia_A sp900548245_MB2bin70	Pathway module_ Carbohydrate and lipid metabolism_ Sugar metabolism_Trehalose biosynthesis, D-glucose 1P => trehalose	0,016	0,057	0,382
Bacteria_Firmicutes_A_Clostridia _Lachnospirales_Lachnospiraceae _Eubacterium_I_Eubacterium_I ramulus_MB2bin11	Pathway module_ Carbohydrate and lipid metabolism_ Sugar metabolism_Trehalose biosynthesis, D-glucose 1P => trehalose	0,001	0,009	0,508
Bacteria_Firmicutes_A_Clostridia _Lachnospirales_Lachnospiraceae _Eubacterium_I_Eubacterium_I sp900546495_MB2bin174	Pathway module_ Carbohydrate and lipid metabolism_ Sugar metabolism_Trehalose biosynthesis, D-glucose 1P => trehalose	0,000	0,001	0,612
Bacteria_Firmicutes_A_Clostridia _Lachnospirales_Lachnospiraceae _Fusicatenibacter_Fusicatenibact er saccharivorans_MB2bin29	Pathway module_ Carbohydrate and lipid metabolism_ Sugar metabolism_Trehalose biosynthesis, D-glucose 1P => trehalose	0,010	0,036	0,408
Bacteria_Firmicutes_A_Clostridia _Lachnospirales_Lachnospiraceae _Mediterraneibacter_Mediterran eibacter faecis_MB2bin127	Pathway module_ Carbohydrate and lipid metabolism_ Sugar metabolism_Trehalose biosynthesis, D-glucose 1P => trehalose	0,000	0,008	0,541
Bacteria_Firmicutes_A_Clostridia _Lachnospirales_Lachnospiraceae _Ruminococcus_A_Ruminococcus _A sp000437095_MB2bin155	Pathway module_ Carbohydrate and lipid metabolism_ Sugar metabolism_Trehalose biosynthesis, D-glucose 1P => trehalose	0,026	0,080	0,356
Bacteria_Firmicutes_A_Clostridia _Lachnospirales_Lachnospiraceae _Ruminococcus_A_Ruminococcus _A sp003011855_MB2bin82	Pathway module_ Carbohydrate and lipid metabolism_ Sugar metabolism_Trehalose biosynthesis, D-glucose 1P => trehalose	0,001	0,008	0,531
Bacteria_Firmicutes_A_Clostridia _Lachnospirales_Lachnospiraceae _UMGS1375_UMGS1375 sp900066615_MB2bin12	Pathway module_ Carbohydrate and lipid metabolism_ Sugar metabolism_Trehalose biosynthesis, D-glucose 1P => trehalose	0,010	0,036	0,410
Bacteria_Firmicutes_A_Clostridia _Lachnospirales_Lachnospirales_ Dorea_Dorea formicigenerans_MB2bin120	Pathway module_ Carbohydrate and lipid metabolism_ Sugar metabolism_Trehalose biosynthesis, D-glucose 1P => trehalose	0,002	0,014	0,479

Bacteria_Firmicutes_A_Clostridia _Lachnospirales_Lachnospirales_ Dorea_Dorea longicatena_MB2bin81	Pathway module_ Carbohydrate and lipid metabolism_ Sugar metabolism_Trehalose biosynthesis, D-glucose 1P => trehalose	0,020	0,067	0,370
Bacteria_Firmicutes_A_Clostridia _Monoglobales_A_UBA1381_CA G-41_?_MB2bin97	Pathway module_ Carbohydrate and lipid metabolism_ Sugar metabolism_Trehalose biosynthesis, D-glucose 1P => trehalose	0,004	0,019	0,450
Bacteria_Firmicutes_A_Clostridia _Oscillospirales_Acutalibacterace ae_Ruminococcus_E_Ruminococc us_E bromii_B_MB2bin205	Pathway module_ Carbohydrate and lipid metabolism_ Sugar metabolism_Trehalose biosynthesis, D-glucose 1P => trehalose	0,027	0,081	0,355
Bacteria_Firmicutes_A_Clostridia _Oscillospirales_Acutalibacterace ae_Ruminococcus_H_Ruminococ cus_H sp003531055_MB2bin93	Pathway module_ Carbohydrate and lipid metabolism_ Sugar metabolism_Trehalose biosynthesis, D-glucose 1P => trehalose	0,032	0,092	0,344
Bacteria_Firmicutes_A_Clostridia _Oscillospirales_Butyricicoccacea e_Agathobaculum_Agathobaculu m butyriciproducens_MB2bin73	Pathway module_ Carbohydrate and lipid metabolism_ Sugar metabolism_Trehalose biosynthesis, D-glucose 1P => trehalose	0,023	0,072	0,364
Bacteria_Firmicutes_A_Clostridia _Oscillospirales_Oscillospiraceae _CAG-103_?_MB2bin101	Pathway module_ Carbohydrate and lipid metabolism_ Sugar metabolism_Trehalose biosynthesis, D-glucose 1P => trehalose	0,001	0,011	-0,493
Bacteria_Firmicutes_A_Clostridia _Oscillospirales_Oscillospiraceae _CAG-170_CAG-170 sp900545925_MB2bin33	Pathway module_ Carbohydrate and lipid metabolism_ Sugar metabolism_Trehalose biosynthesis, D-glucose 1P => trehalose	0,004	0,019	-0,452
Bacteria_Firmicutes_A_Clostridia _Oscillospirales_Oscillospiraceae _CAG-83_?_MB2bin100	Pathway module_ Carbohydrate and lipid metabolism_ Sugar metabolism_Trehalose biosynthesis, D-glucose 1P => trehalose	0,018	0,062	-0,376
Bacteria_Firmicutes_A_Clostridia _Oscillospirales_Oscillospiraceae _CAG-83_?_MB2bin179	Pathway module_ Carbohydrate and lipid metabolism_ Sugar metabolism_Trehalose biosynthesis, D-glucose 1P => trehalose	0,002	0,011	-0,487
Bacteria_Firmicutes_A_Clostridia _Oscillospirales_Oscillospiraceae _ER4_ER4 sp000765235_MB2bin20	Pathway module_ Carbohydrate and lipid metabolism_ Sugar metabolism_Trehalose biosynthesis, D-glucose 1P => trehalose	0,004	0,019	-0,451

Bacteria_Firmicutes_A_Clostridia_Oscillospirales_Oscillospiraceae_Oscillibacter_?_MB2bin147	Pathway module_ Carbohydrate and lipid metabolism_ Sugar metabolism_Trehalose biosynthesis, D-glucose 1P => trehalose	0,004	0,019	-0,453
Bacteria_Firmicutes_A_Clostridia_Oscillospirales_Ruminococcaceae_Faecalibacterium_Faecalibacterium prausnitzii_A_MB2bin78	Pathway module_ Carbohydrate and lipid metabolism_ Sugar metabolism_Trehalose biosynthesis, D-glucose 1P => trehalose	0,004	0,019	0,454
Bacteria_Firmicutes_A_Clostridia_Oscillospirales_Ruminococcaceae_Faecalibacterium_Faecalibacterium prausnitzii_C_MB2bin90	Pathway module_ Carbohydrate and lipid metabolism_ Sugar metabolism_Trehalose biosynthesis, D-glucose 1P => trehalose	0,001	0,008	0,531
Bacteria_Firmicutes_A_Clostridia_Oscillospirales_Ruminococcaceae_Faecalibacterium_Faecalibacterium prausnitzii_G_MB2bin45	Pathway module_ Carbohydrate and lipid metabolism_ Sugar metabolism_Trehalose biosynthesis, D-glucose 1P => trehalose	0,004	0,020	0,445
Bacteria_Firmicutes_A_Clostridia_Oscillospirales_Ruminococcaceae_Faecalibacterium_Faecalibacterium prausnitzii_H_MB2bin261	Pathway module_ Carbohydrate and lipid metabolism_ Sugar metabolism_Trehalose biosynthesis, D-glucose 1P => trehalose	0,006	0,025	0,433
Bacteria_Firmicutes_A_Clostridia_Oscillospirales_Ruminococcaceae_Gemmiger_Gemmiger sp900539695_MB2bin39	Pathway module_ Carbohydrate and lipid metabolism_ Sugar metabolism_Trehalose biosynthesis, D-glucose 1P => trehalose	0,006	0,024	0,435
Bacteria_Firmicutes_A_Clostridia_Oscillospirales_Ruminococcaceae_Ruminiclostridium_E_Ruminiclostridium_E siraeum_MB2bin52	Pathway module_ Carbohydrate and lipid metabolism_ Sugar metabolism_Trehalose biosynthesis, D-glucose 1P => trehalose	0,002	0,011	-0,488
Bacteria_Firmicutes_A_Clostridia_Oscillospirales_Ruminococcaceae_Ruminiclostridium_E_Ruminiclostridium_E sp003512525_MB2bin228	Pathway module_ Carbohydrate and lipid metabolism_ Sugar metabolism_Trehalose biosynthesis, D-glucose 1P => trehalose	0,001	0,009	-0,505
Bacteria_Firmicutes_I_Bacilli_A_Lactobacillales_Streptococcaceae_Streptococcus_?_MB2bin209	Pathway module_ Carbohydrate and lipid metabolism_ Sugar metabolism_Trehalose biosynthesis, D-glucose 1P => trehalose	0,015	0,053	0,388
Bacteria_Actinobacteriota_Actinobacteria_Actinomycetales_Bifidobacteriaceae_Bifidobacterium_Bifidobacterium ruminantium_specI_v3_Cluster2702	Pathway module_ Nucleotide and amino acid metabolism_ Cofactor and vitamin biosynthesis_Biotin biosynthesis, BioW pathway, pimelate => pimeloyl-CoA => biotin	0,002	0,016	-0,485

Bacteria_Actinobacteriota_Actinomycetia_Actinomycetales_Bifidobacteriaceae_Bifidobacterium_Bifidobacterium adolescentis_MB2bin135	Pathway module_ Nucleotide and amino acid metabolism_ Cofactor and vitamin biosynthesis_Biotin biosynthesis, BioW pathway, pimelate => pimeloyl-CoA => biotin	0,004	0,025	-0,455
Bacteria_Actinobacteriota_Actinomycetia_Actinomycetales_Bifidobacteriaceae_Bifidobacterium_Bifidobacterium angulatum_MB2bin288	Pathway module_ Nucleotide and amino acid metabolism_ Cofactor and vitamin biosynthesis_Biotin biosynthesis, BioW pathway, pimelate => pimeloyl-CoA => biotin	0,016	0,061	-0,382
Bacteria_Actinobacteriota_Coriobacteriia_Coriobacteriales_Coriobacteriaceae_Collinsella_Collinsella aerofaciens_F_specI_v3_Cluster3625	Pathway module_ Nucleotide and amino acid metabolism_ Cofactor and vitamin biosynthesis_Biotin biosynthesis, BioW pathway, pimelate => pimeloyl-CoA => biotin	0,028	0,087	-0,353
Bacteria_Actinobacteriota_Coriobacteriia_Coriobacteriales_Eggerthellaceae_CAG-1427_?_MB2bin289	Pathway module_ Nucleotide and amino acid metabolism_ Cofactor and vitamin biosynthesis_Biotin biosynthesis, BioW pathway, pimelate => pimeloyl-CoA => biotin	0,030	0,093	-0,347
Bacteria_Bacteroidota_Bacteroidia_Bacteroidales_Bacteroidaceae_Bacteroides_B_Bacteroides_B sartorii_specI_v3_Cluster2366	Pathway module_ Nucleotide and amino acid metabolism_ Cofactor and vitamin biosynthesis_Biotin biosynthesis, BioW pathway, pimelate => pimeloyl-CoA => biotin	0,000	0,000	0,664
Bacteria_Bacteroidota_Bacteroidia_Bacteroidales_Bacteroidaceae_Bacteroides_Bacteroides eggerthii_MB2bin146	Pathway module_ Nucleotide and amino acid metabolism_ Cofactor and vitamin biosynthesis_Biotin biosynthesis, BioW pathway, pimelate => pimeloyl-CoA => biotin	0,000	0,000	0,738
Bacteria_Bacteroidota_Bacteroidia_Bacteroidales_Bacteroidaceae_Bacteroides_Bacteroides fragilis_MB2bin178	Pathway module_ Nucleotide and amino acid metabolism_ Cofactor and vitamin biosynthesis_Biotin biosynthesis, BioW pathway, pimelate => pimeloyl-CoA => biotin	0,013	0,052	0,392

Bacteria_Bacteroidota_Bacteroidia_Bacteroidales_Bacteroidaceae_Bacteroides_Bacteroides ovatus_MB2bin110	Pathway module_ Nucleotide and amino acid metabolism_ Cofactor and vitamin biosynthesis_Biotin biosynthesis, BioW pathway, pimelate => pimeloyl-CoA => biotin	0,000	0,000	0,693
Bacteria_Bacteroidota_Bacteroidia_Bacteroidales_Bacteroidaceae_Bacteroides_Bacteroides stercoris_MB2bin42	Pathway module_ Nucleotide and amino acid metabolism_ Cofactor and vitamin biosynthesis_Biotin biosynthesis, BioW pathway, pimelate => pimeloyl-CoA => biotin	0,000	0,000	0,756
Bacteria_Bacteroidota_Bacteroidia_Bacteroidales_Bacteroidaceae_Bacteroides_Bacteroides thetaiotaomicron_MB2bin137	Pathway module_ Nucleotide and amino acid metabolism_ Cofactor and vitamin biosynthesis_Biotin biosynthesis, BioW pathway, pimelate => pimeloyl-CoA => biotin	0,000	0,000	0,643
Bacteria_Bacteroidota_Bacteroidia_Bacteroidales_Bacteroidaceae_Bacteroides_Bacteroides uniformis_MB2bin1	Pathway module_ Nucleotide and amino acid metabolism_ Cofactor and vitamin biosynthesis_Biotin biosynthesis, BioW pathway, pimelate => pimeloyl-CoA => biotin	0,000	0,001	0,618
Bacteria_Bacteroidota_Bacteroidia_Bacteroidales_Bacteroidaceae_Phocaeicola_Phocaeicola massiliensis_MB2bin187	Pathway module_ Nucleotide and amino acid metabolism_ Cofactor and vitamin biosynthesis_Biotin biosynthesis, BioW pathway, pimelate => pimeloyl-CoA => biotin	0,002	0,015	0,489
Bacteria_Bacteroidota_Bacteroidia_Bacteroidales_Bacteroidaceae_Phocaeicola_Phocaeicola vulgatus_MB2bin4	Pathway module_ Nucleotide and amino acid metabolism_ Cofactor and vitamin biosynthesis_Biotin biosynthesis, BioW pathway, pimelate => pimeloyl-CoA => biotin	0,000	0,000	0,665
Bacteria_Bacteroidota_Bacteroidia_Bacteroidales_Bacteroidaceae_Prevotella_?_MB2bin51	Pathway module_ Nucleotide and amino acid metabolism_ Cofactor and vitamin biosynthesis_Biotin biosynthesis, BioW pathway, pimelate => pimeloyl-CoA => biotin	0,012	0,049	-0,398

Bacteria_Bacteroidota_Bacteroidia_Bacteroidales_Bacteroidaceae_Prevotella_Prevotella copri_A_MB2bin109	Pathway module_ Nucleotide and amino acid metabolism_ Cofactor and vitamin biosynthesis_Biotin biosynthesis, BioW pathway, pimelate => pimeloyl-CoA => biotin	0,000	0,003	-0,564
Bacteria_Bacteroidota_Bacteroidia_Bacteroidales_Barnesiellaceae_Barnesiella_Barnesiella intestinihominis_MB2bin112	Pathway module_ Nucleotide and amino acid metabolism_ Cofactor and vitamin biosynthesis_Biotin biosynthesis, BioW pathway, pimelate => pimeloyl-CoA => biotin	0,000	0,003	0,573
Bacteria_Bacteroidota_Bacteroidia_Bacteroidales_Rikenellaceae_Alistipes_A_Alistipes_A ihumii_MB2bin204	Pathway module_ Nucleotide and amino acid metabolism_ Cofactor and vitamin biosynthesis_Biotin biosynthesis, BioW pathway, pimelate => pimeloyl-CoA => biotin	0,007	0,038	0,425
Bacteria_Bacteroidota_Bacteroidia_Bacteroidales_Rikenellaceae_Alistipes_Alistipes finegoldii_MB2bin46	Pathway module_ Nucleotide and amino acid metabolism_ Cofactor and vitamin biosynthesis_Biotin biosynthesis, BioW pathway, pimelate => pimeloyl-CoA => biotin	0,004	0,025	0,454
Bacteria_Bacteroidota_Bacteroidia_Bacteroidales_Rikenellaceae_Alistipes_Alistipes obesi_MB2bin28	Pathway module_ Nucleotide and amino acid metabolism_ Cofactor and vitamin biosynthesis_Biotin biosynthesis, BioW pathway, pimelate => pimeloyl-CoA => biotin	0,001	0,007	0,523
Bacteria_Bacteroidota_Bacteroidia_Bacteroidales_Rikenellaceae_Alistipes_Alistipes onderdonkii_MB2bin22	Pathway module_ Nucleotide and amino acid metabolism_ Cofactor and vitamin biosynthesis_Biotin biosynthesis, BioW pathway, pimelate => pimeloyl-CoA => biotin	0,028	0,087	0,353
Bacteria_Bacteroidota_Bacteroidia_Bacteroidales_Rikenellaceae_Alistipes_Alistipes shahii_MB2bin10	Pathway module_ Nucleotide and amino acid metabolism_ Cofactor and vitamin biosynthesis_Biotin biosynthesis, BioW pathway, pimelate => pimeloyl-CoA => biotin	0,000	0,000	0,652

Bacteria_Bacteroidota_Bacteroidia_Bacteroidales_Rikenellaceae_Alistipes_Alistipes sp900083545_specI_v3_Cluster1016	Pathway module_ Nucleotide and amino acid metabolism_ Cofactor and vitamin biosynthesis_Biotin biosynthesis, BioW pathway, pimelate => pimeloyl-CoA => biotin	0,000	0,004	0,545
Bacteria_Bacteroidota_Bacteroidia_Bacteroidales_Tannerellaceae_Parabacteroides_Parabacteroides distasonis_MB2bin71	Pathway module_ Nucleotide and amino acid metabolism_ Cofactor and vitamin biosynthesis_Biotin biosynthesis, BioW pathway, pimelate => pimeloyl-CoA => biotin	0,003	0,023	0,461
Bacteria_Bacteroidota_Bacteroidia_Bacteroidales_Tannerellaceae_Parabacteroides_Parabacteroides johnsonii_MB2bin185	Pathway module_ Nucleotide and amino acid metabolism_ Cofactor and vitamin biosynthesis_Biotin biosynthesis, BioW pathway, pimelate => pimeloyl-CoA => biotin	0,000	0,003	0,556
Bacteria_Bacteroidota_Bacteroidia_Bacteroidales_Tannerellaceae_Parabacteroides_Parabacteroides merdae_MB2bin5	Pathway module_ Nucleotide and amino acid metabolism_ Cofactor and vitamin biosynthesis_Biotin biosynthesis, BioW pathway, pimelate => pimeloyl-CoA => biotin	0,000	0,003	0,564
Bacteria_Firmicutes_A_Clostridia_A_Christensenellales_CAG-74_SFFH01_SFFH01 sp900542445_MB2bin113	Pathway module_ Nucleotide and amino acid metabolism_ Cofactor and vitamin biosynthesis_Biotin biosynthesis, BioW pathway, pimelate => pimeloyl-CoA => biotin	0,014	0,052	-0,392
Bacteria_Firmicutes_A_Clostridia_Lachnospirales_Lachnospiraceae_Anaerobutyricum_?_MB2bin124	Pathway module_ Nucleotide and amino acid metabolism_ Cofactor and vitamin biosynthesis_Biotin biosynthesis, BioW pathway, pimelate => pimeloyl-CoA => biotin	0,007	0,038	-0,428
Bacteria_Firmicutes_A_Clostridia_Lachnospirales_Lachnospiraceae_Anaerobutyricum_Anaerobutyricum hallii_MB2bin3	Pathway module_ Nucleotide and amino acid metabolism_ Cofactor and vitamin biosynthesis_Biotin biosynthesis, BioW pathway, pimelate => pimeloyl-CoA => biotin	0,027	0,087	-0,354

Bacteria_Firmicutes_A_Clostridia_Lachnospirales_Lachnospiraceae_Anaerostipes_Anaerostipes hadrus_A_specI_v3_Cluster856	Pathway module_ Nucleotide and amino acid metabolism_ Cofactor and vitamin biosynthesis_Biotin biosynthesis, BioW pathway, pimelate => pimeloyl-CoA => biotin	0,006	0,038	-0,429
Bacteria_Firmicutes_A_Clostridia_Lachnospirales_Lachnospiraceae_Bariatricus_Bariatricus comes_MB2bin19	Pathway module_ Nucleotide and amino acid metabolism_ Cofactor and vitamin biosynthesis_Biotin biosynthesis, BioW pathway, pimelate => pimeloyl-CoA => biotin	0,026	0,087	-0,356
Bacteria_Firmicutes_A_Clostridia_Lachnospirales_Lachnospiraceae_Blautia_A_?_MB2bin128	Pathway module_ Nucleotide and amino acid metabolism_ Cofactor and vitamin biosynthesis_Biotin biosynthesis, BioW pathway, pimelate => pimeloyl-CoA => biotin	0,018	0,064	-0,377
Bacteria_Firmicutes_A_Clostridia_Lachnospirales_Lachnospiraceae_Blautia_A_?_MB2bin14	Pathway module_ Nucleotide and amino acid metabolism_ Cofactor and vitamin biosynthesis_Biotin biosynthesis, BioW pathway, pimelate => pimeloyl-CoA => biotin	0,024	0,081	-0,362
Bacteria_Firmicutes_A_Clostridia_Lachnospirales_Lachnospiraceae_Blautia_A_?_MB2bin175	Pathway module_ Nucleotide and amino acid metabolism_ Cofactor and vitamin biosynthesis_Biotin biosynthesis, BioW pathway, pimelate => pimeloyl-CoA => biotin	0,009	0,041	-0,412
Bacteria_Firmicutes_A_Clostridia_Lachnospirales_Lachnospiraceae_Blautia_A_Blautia_A massiliensis_MB2bin79	Pathway module_ Nucleotide and amino acid metabolism_ Cofactor and vitamin biosynthesis_Biotin biosynthesis, BioW pathway, pimelate => pimeloyl-CoA => biotin	0,003	0,023	-0,463
Bacteria_Firmicutes_A_Clostridia_Lachnospirales_Lachnospiraceae_Blautia_A_Blautia_A sp900066335_MB2bin25	Pathway module_ Nucleotide and amino acid metabolism_ Cofactor and vitamin biosynthesis_Biotin biosynthesis, BioW pathway, pimelate => pimeloyl-CoA => biotin	0,001	0,015	-0,491

Bacteria_Firmicutes_A_Clostridia _Lachnospirales_Lachnospiraceae _Blautia_A_Blautia_A sp900066355_MGS00045	Pathway module_ Nucleotide and amino acid metabolism_ Cofactor and vitamin biosynthesis_Biotin biosynthesis, BioW pathway, pimelate => pimeloyl-CoA => biotin	0,021	0,073	-0,369
Bacteria_Firmicutes_A_Clostridia _Lachnospirales_Lachnospiraceae _Coprococcus_A_Coprococcus_A catus_MB2bin38	Pathway module_ Nucleotide and amino acid metabolism_ Cofactor and vitamin biosynthesis_Biotin biosynthesis, BioW pathway, pimelate => pimeloyl-CoA => biotin	0,007	0,038	-0,424
Bacteria_Firmicutes_A_Clostridia _Lachnospirales_Lachnospiraceae _Coprococcus_Coprococcus eutactus_A_MB2bin7	Pathway module_ Nucleotide and amino acid metabolism_ Cofactor and vitamin biosynthesis_Biotin biosynthesis, BioW pathway, pimelate => pimeloyl-CoA => biotin	0,010	0,044	-0,405
Bacteria_Firmicutes_A_Clostridia _Lachnospirales_Lachnospiraceae _Coprococcus_Coprococcus eutactus_MB2bin153	Pathway module_ Nucleotide and amino acid metabolism_ Cofactor and vitamin biosynthesis_Biotin biosynthesis, BioW pathway, pimelate => pimeloyl-CoA => biotin	0,011	0,044	-0,405
Bacteria_Firmicutes_A_Clostridia _Lachnospirales_Lachnospiraceae _Mediterraneibacter_Mediterran eibacter faecis_MB2bin127	Pathway module_ Nucleotide and amino acid metabolism_ Cofactor and vitamin biosynthesis_Biotin biosynthesis, BioW pathway, pimelate => pimeloyl-CoA => biotin	0,003	0,023	-0,462
Bacteria_Firmicutes_A_Clostridia _Lachnospirales_Lachnospiraceae _Ruminococcus_A_Ruminococcus _A sp000437095_MB2bin155	Pathway module_ Nucleotide and amino acid metabolism_ Cofactor and vitamin biosynthesis_Biotin biosynthesis, BioW pathway, pimelate => pimeloyl-CoA => biotin	0,010	0,044	-0,407
Bacteria_Firmicutes_A_Clostridia _Lachnospirales_Lachnospiraceae _Ruminococcus_A_Ruminococcus _A sp003011855_MB2bin82	Pathway module_ Nucleotide and amino acid metabolism_ Cofactor and vitamin biosynthesis_Biotin biosynthesis, BioW pathway, pimelate => pimeloyl-CoA => biotin	0,001	0,010	-0,510

Bacteria_Firmicutes_A_Clostridia_Lachnospirales_Lachnospirales_Dorea_Dorea formicigenerans_MB2bin120	Pathway module_ Nucleotide and amino acid metabolism_ Cofactor and vitamin biosynthesis_Biotin biosynthesis, BioW pathway, pimelate => pimeloyl-CoA => biotin	0,000	0,003	-0,559
Bacteria_Firmicutes_A_Clostridia_Lachnospirales_Lachnospirales_Dorea_Dorea longicatena_MB2bin81	Pathway module_ Nucleotide and amino acid metabolism_ Cofactor and vitamin biosynthesis_Biotin biosynthesis, BioW pathway, pimelate => pimeloyl-CoA => biotin	0,009	0,041	-0,413
Bacteria_Firmicutes_A_Clostridia_Oscillospirales_Acutalibacteraceae_Ruminococcus_H_Ruminococcus_H sp003531055_MB2bin93	Pathway module_ Nucleotide and amino acid metabolism_ Cofactor and vitamin biosynthesis_Biotin biosynthesis, BioW pathway, pimelate => pimeloyl-CoA => biotin	0,028	0,087	-0,352
Bacteria_Firmicutes_A_Clostridia_Oscillospirales_Oscillospiraceae_CAG-83_?_MB2bin179	Pathway module_ Nucleotide and amino acid metabolism_ Cofactor and vitamin biosynthesis_Biotin biosynthesis, BioW pathway, pimelate => pimeloyl-CoA => biotin	0,005	0,035	0,436
Bacteria_Firmicutes_A_Clostridia_Oscillospirales_Oscillospiraceae_Oscillibacter_?_MB2bin147	Pathway module_ Nucleotide and amino acid metabolism_ Cofactor and vitamin biosynthesis_Biotin biosynthesis, BioW pathway, pimelate => pimeloyl-CoA => biotin	0,008	0,041	0,418
Bacteria_Firmicutes_A_Clostridia_Oscillospirales_Ruminococcaceae_Faecalibacterium_Faecalibacterium prausnitzii_A_MB2bin78	Pathway module_ Nucleotide and amino acid metabolism_ Cofactor and vitamin biosynthesis_Biotin biosynthesis, BioW pathway, pimelate => pimeloyl-CoA => biotin	0,017	0,064	-0,379
Bacteria_Firmicutes_A_Clostridia_Oscillospirales_Ruminococcaceae_Faecalibacterium_Faecalibacterium prausnitzii_C_MB2bin90	Pathway module_ Nucleotide and amino acid metabolism_ Cofactor and vitamin biosynthesis_Biotin biosynthesis, BioW pathway, pimelate => pimeloyl-CoA => biotin	0,004	0,026	-0,452

Bacteria_Firmicutes_A_Clostridia_Oscillospirales_Ruminococcaceae_Gemmiger_Gemmiger sp900539695_MB2bin39	Pathway module_ Nucleotide and amino acid metabolism_ Cofactor and vitamin biosynthesis_Biotin biosynthesis, BioW pathway, pimelate => pimeloyl-CoA => biotin	0,013	0,052	-0,393
Bacteria_Firmicutes_A_Clostridia_Oscillospirales_Ruminococcaceae_Gemmiger_Gemmiger sp900540595_MB2bin67	Pathway module_ Nucleotide and amino acid metabolism_ Cofactor and vitamin biosynthesis_Biotin biosynthesis, BioW pathway, pimelate => pimeloyl-CoA => biotin	0,009	0,041	-0,414
Bacteria_Firmicutes_A_Clostridia_TANB77_CAG-508_CAG-492_CAG-492 sp000434015_specI_v3_Cluster7639	Pathway module_ Nucleotide and amino acid metabolism_ Cofactor and vitamin biosynthesis_Biotin biosynthesis, BioW pathway, pimelate => pimeloyl-CoA => biotin	0,008	0,041	-0,420
Bacteria_Firmicutes_C_Negativicutes_Veillonellales_Dialisteraceae_Dialister_Dialister invisus_specI_v3_Cluster3691	Pathway module_ Nucleotide and amino acid metabolism_ Cofactor and vitamin biosynthesis_Biotin biosynthesis, BioW pathway, pimelate => pimeloyl-CoA => biotin	0,009	0,041	0,412
Bacteria_Firmicutes_I_Bacilli_A_Lactobacillales_Streptococcaceae_Streptococcus_?_MB2bin209	Pathway module_ Nucleotide and amino acid metabolism_ Cofactor and vitamin biosynthesis_Biotin biosynthesis, BioW pathway, pimelate => pimeloyl-CoA => biotin	0,002	0,016	-0,482
Bacteria_Firmicutes_I_Bacilli_A_Lactobacillales_Streptococcaceae_Streptococcus_Streptococcus thermophilus_MB2bin145	Pathway module_ Nucleotide and amino acid metabolism_ Cofactor and vitamin biosynthesis_Biotin biosynthesis, BioW pathway, pimelate => pimeloyl-CoA => biotin	0,006	0,036	-0,433
Bacteria_Verrucomicrobiota_Verrucomicrobiae_Verrucomicrobiales_Akkermansiaceae_Akkermansia_Akkermansia muciniphila_B_MB2bin125	Pathway module_ Nucleotide and amino acid metabolism_ Cofactor and vitamin biosynthesis_Biotin biosynthesis, BioW pathway, pimelate => pimeloyl-CoA => biotin	0,032	0,096	0,344

Bacteria_Actinobacteriota_Actinobacteria_Actinomycetales_Bifidobacteriaceae_Bifidobacterium_Bifidobacterium breve_specI_v3_Cluster1098	Pathway module_ Nucleotide and amino acid metabolism_ Cofactor and vitamin biosynthesis_Glutathione biosynthesis, glutamate => glutathione	0,014	0,065	0,390
Bacteria_Actinobacteriota_Actinobacteria_Actinomycetales_Bifidobacteriaceae_Bifidobacterium_Bifidobacterium ruminantium_specI_v3_Cluster2702	Pathway module_ Nucleotide and amino acid metabolism_ Cofactor and vitamin biosynthesis_Glutathione biosynthesis, glutamate => glutathione	0,000	0,000	0,674
Bacteria_Actinobacteriota_Actinomycetia_Actinomycetales_Bifidobacteriaceae_Bifidobacterium_Bifidobacterium adolescentis_MB2bin135	Pathway module_ Nucleotide and amino acid metabolism_ Cofactor and vitamin biosynthesis_Glutathione biosynthesis, glutamate => glutathione	0,000	0,000	0,655
Bacteria_Actinobacteriota_Actinomycetia_Actinomycetales_Bifidobacteriaceae_Bifidobacterium_Bifidobacterium angulatum_MB2bin288	Pathway module_ Nucleotide and amino acid metabolism_ Cofactor and vitamin biosynthesis_Glutathione biosynthesis, glutamate => glutathione	0,000	0,000	0,733
Bacteria_Actinobacteriota_Actinomycetia_Actinomycetales_Bifidobacteriaceae_Bifidobacterium_Bifidobacterium longum_MB2bin141	Pathway module_ Nucleotide and amino acid metabolism_ Cofactor and vitamin biosynthesis_Glutathione biosynthesis, glutamate => glutathione	0,006	0,043	0,430
Bacteria_Actinobacteriota_Coriobacteriia_Coriobacteriales_Coriobacteriaceae_Collinsella_?_MB2bin75	Pathway module_ Nucleotide and amino acid metabolism_ Cofactor and vitamin biosynthesis_Glutathione biosynthesis, glutamate => glutathione	0,018	0,077	0,376
Bacteria_Actinobacteriota_Coriobacteriia_Coriobacteriales_Coriobacteriaceae_Collinsella_Collinsella sp000763055_MB2bin202	Pathway module_ Nucleotide and amino acid metabolism_ Cofactor and vitamin biosynthesis_Glutathione biosynthesis, glutamate => glutathione	0,009	0,052	0,411
Bacteria_Bacteroidota_Bacteroidia_Bacteroidales_Bacteroidaceae_Bacteroides_B_Bacteroides_B sartorii_specI_v3_Cluster2366	Pathway module_ Nucleotide and amino acid metabolism_ Cofactor and vitamin biosynthesis_Glutathione biosynthesis, glutamate => glutathione	0,000	0,006	-0,538
Bacteria_Bacteroidota_Bacteroidia_Bacteroidales_Bacteroidaceae_Bacteroides_Bacteroides eggerthii_MB2bin146	Pathway module_ Nucleotide and amino acid metabolism_ Cofactor and vitamin biosynthesis_Glutathione	0,027	0,098	-0,355

	biosynthesis, glutamate => glutathione			
Bacteria_Bacteroidota_Bacteroidia_Bacteroidales_Bacteroidaceae_Bacteroides_Bacteroides ovatus_MB2bin110	Pathway module_ Nucleotide and amino acid metabolism_ Cofactor and vitamin biosynthesis_Glutathione biosynthesis, glutamate => glutathione	0,001	0,011	-0,508
Bacteria_Bacteroidota_Bacteroidia_Bacteroidales_Bacteroidaceae_Bacteroides_Bacteroides stercoris_MB2bin42	Pathway module_ Nucleotide and amino acid metabolism_ Cofactor and vitamin biosynthesis_Glutathione biosynthesis, glutamate => glutathione	0,013	0,064	-0,394
Bacteria_Bacteroidota_Bacteroidia_Bacteroidales_Bacteroidaceae_Bacteroides_Bacteroides thetaiotaomicron_MB2bin137	Pathway module_ Nucleotide and amino acid metabolism_ Cofactor and vitamin biosynthesis_Glutathione biosynthesis, glutamate => glutathione	0,003	0,029	-0,458
Bacteria_Bacteroidota_Bacteroidia_Bacteroidales_Bacteroidaceae_Phocaeicola_Phocaeicola vulgatus_MB2bin4	Pathway module_ Nucleotide and amino acid metabolism_ Cofactor and vitamin biosynthesis_Glutathione biosynthesis, glutamate => glutathione	0,000	0,003	-0,572
Bacteria_Bacteroidota_Bacteroidia_Bacteroidales_Barnesiellaceae_Barnesiella_Barnesiella intestinihominis_MB2bin112	Pathway module_ Nucleotide and amino acid metabolism_ Cofactor and vitamin biosynthesis_Glutathione biosynthesis, glutamate => glutathione	0,000	0,006	-0,539
Bacteria_Bacteroidota_Bacteroidia_Bacteroidales_Rikenellaceae_Alistipes_Alistipes finegoldii_MB2bin46	Pathway module_ Nucleotide and amino acid metabolism_ Cofactor and vitamin biosynthesis_Glutathione biosynthesis, glutamate => glutathione	0,001	0,010	-0,516
Bacteria_Bacteroidota_Bacteroidia_Bacteroidales_Rikenellaceae_Alistipes_Alistipes obesi_MB2bin28	Pathway module_ Nucleotide and amino acid metabolism_ Cofactor and vitamin biosynthesis_Glutathione biosynthesis, glutamate => glutathione	0,000	0,003	-0,578
Bacteria_Bacteroidota_Bacteroidia_Bacteroidales_Rikenellaceae_Alistipes_Alistipes onderdonkii_MB2bin22	Pathway module_ Nucleotide and amino acid metabolism_ Cofactor and vitamin biosynthesis_Glutathione biosynthesis, glutamate => glutathione	0,022	0,088	-0,365

Bacteria_Bacteroidota_Bacteroidia_Bacteroidales_Rikenellaceae_Alistipes_Alistipes shahii_MB2bin10	Pathway module_ Nucleotide and amino acid metabolism_ Cofactor and vitamin biosynthesis_Glutathione biosynthesis, glutamate => glutathione	0,000	0,003	-0,576
Bacteria_Bacteroidota_Bacteroidia_Bacteroidales_Rikenellaceae_Alistipes_Alistipes sp900083545_specI_v3_Cluster1016	Pathway module_ Nucleotide and amino acid metabolism_ Cofactor and vitamin biosynthesis_Glutathione biosynthesis, glutamate => glutathione	0,000	0,004	-0,559
Bacteria_Bacteroidota_Bacteroidia_Bacteroidales_Tannerellaceae_Parabacteroides_Parabacteroides johnsonii_MB2bin185	Pathway module_ Nucleotide and amino acid metabolism_ Cofactor and vitamin biosynthesis_Glutathione biosynthesis, glutamate => glutathione	0,003	0,029	-0,463
Bacteria_Bacteroidota_Bacteroidia_Bacteroidales_Tannerellaceae_Parabacteroides_Parabacteroides merdae_MB2bin5	Pathway module_ Nucleotide and amino acid metabolism_ Cofactor and vitamin biosynthesis_Glutathione biosynthesis, glutamate => glutathione	0,008	0,050	-0,417
Bacteria_Firmicutes_A_Clostridia_A_Christensenellales_QAND01_UMGS1975_UMGS1975 sp900546685_MB2bin272	Pathway module_ Nucleotide and amino acid metabolism_ Cofactor and vitamin biosynthesis_Glutathione biosynthesis, glutamate => glutathione	0,001	0,007	0,528
Bacteria_Firmicutes_A_Clostridia_Lachnospirales_Lachnospiraceae_Anaerobutyricum_?_MB2bin124	Pathway module_ Nucleotide and amino acid metabolism_ Cofactor and vitamin biosynthesis_Glutathione biosynthesis, glutamate => glutathione	0,000	0,001	0,633
Bacteria_Firmicutes_A_Clostridia_Lachnospirales_Lachnospiraceae_Anaerobutyricum_Anaerobutyricum hallii_MB2bin3	Pathway module_ Nucleotide and amino acid metabolism_ Cofactor and vitamin biosynthesis_Glutathione biosynthesis, glutamate => glutathione	0,000	0,002	0,598
Bacteria_Firmicutes_A_Clostridia_Lachnospirales_Lachnospiraceae_Anaerostipes_Anaerostipes hadrus_A_specI_v3_Cluster856	Pathway module_ Nucleotide and amino acid metabolism_ Cofactor and vitamin biosynthesis_Glutathione biosynthesis, glutamate => glutathione	0,007	0,043	0,426
Bacteria_Firmicutes_A_Clostridia_Lachnospirales_Lachnospiraceae_Anaerostipes_Anaerostipes hadrus_MB2bin47	Pathway module_ Nucleotide and amino acid metabolism_ Cofactor and vitamin biosynthesis_Glutathione	0,004	0,034	0,447

	biosynthesis, glutamate => glutathione			
Bacteria_Firmicutes_A_Clostridia_Lachnospirales_Lachnospiraceae_Blautia_A_?_MB2bin128	Pathway module_ Nucleotide and amino acid metabolism_ Cofactor and vitamin biosynthesis_Glutathione biosynthesis, glutamate => glutathione	0,006	0,043	0,432
Bacteria_Firmicutes_A_Clostridia_Lachnospirales_Lachnospiraceae_Blautia_A_?_MB2bin14	Pathway module_ Nucleotide and amino acid metabolism_ Cofactor and vitamin biosynthesis_Glutathione biosynthesis, glutamate => glutathione	0,000	0,003	0,582
Bacteria_Firmicutes_A_Clostridia_Lachnospirales_Lachnospiraceae_Blautia_A_?_MB2bin175	Pathway module_ Nucleotide and amino acid metabolism_ Cofactor and vitamin biosynthesis_Glutathione biosynthesis, glutamate => glutathione	0,006	0,043	0,433
Bacteria_Firmicutes_A_Clostridia_Lachnospirales_Lachnospiraceae_Blautia_A_Blautia_A massiliensis_MB2bin79	Pathway module_ Nucleotide and amino acid metabolism_ Cofactor and vitamin biosynthesis_Glutathione biosynthesis, glutamate => glutathione	0,013	0,064	0,394
Bacteria_Firmicutes_A_Clostridia_Lachnospirales_Lachnospiraceae_Blautia_A_Blautia_A sp900066145_MB2bin111	Pathway module_ Nucleotide and amino acid metabolism_ Cofactor and vitamin biosynthesis_Glutathione biosynthesis, glutamate => glutathione	0,011	0,059	0,402
Bacteria_Firmicutes_A_Clostridia_Lachnospirales_Lachnospiraceae_Blautia_A_Blautia_A sp900066165_MB2bin2	Pathway module_ Nucleotide and amino acid metabolism_ Cofactor and vitamin biosynthesis_Glutathione biosynthesis, glutamate => glutathione	0,028	0,098	0,352
Bacteria_Firmicutes_A_Clostridia_Lachnospirales_Lachnospiraceae_Blautia_A_Blautia_A sp900066335_MB2bin25	Pathway module_ Nucleotide and amino acid metabolism_ Cofactor and vitamin biosynthesis_Glutathione biosynthesis, glutamate => glutathione	0,007	0,045	0,422
Bacteria_Firmicutes_A_Clostridia_Lachnospirales_Lachnospiraceae_Blautia_A_Blautia_A sp900066355_MGS00045	Pathway module_ Nucleotide and amino acid metabolism_ Cofactor and vitamin biosynthesis_Glutathione biosynthesis, glutamate => glutathione	0,003	0,029	0,458

Bacteria_Firmicutes_A_Clostridia_Lachnospirales_Lachnospiraceae_Blautia_A_Blautia_A sp900548245_MB2bin70	Pathway module_ Nucleotide and amino acid metabolism_ Cofactor and vitamin biosynthesis_Glutathione biosynthesis, glutamate => glutathione	0,020	0,081	0,371
Bacteria_Firmicutes_A_Clostridia_Lachnospirales_Lachnospiraceae_Mediterraneibacter_Mediterraneibacter faecis_MB2bin127	Pathway module_ Nucleotide and amino acid metabolism_ Cofactor and vitamin biosynthesis_Glutathione biosynthesis, glutamate => glutathione	0,016	0,073	0,383
Bacteria_Firmicutes_A_Clostridia_Lachnospirales_Lachnospiraceae_Ruminococcus_A_Ruminococcus_A sp003011855_MB2bin82	Pathway module_ Nucleotide and amino acid metabolism_ Cofactor and vitamin biosynthesis_Glutathione biosynthesis, glutamate => glutathione	0,007	0,043	0,427
Bacteria_Firmicutes_A_Clostridia_Lachnospirales_Lachnospiraceae_Ruminococcus_B_Ruminococcus_B gnavus_MB2bin121	Pathway module_ Nucleotide and amino acid metabolism_ Cofactor and vitamin biosynthesis_Glutathione biosynthesis, glutamate => glutathione	0,027	0,098	0,353
Bacteria_Firmicutes_A_Clostridia_Lachnospirales_Lachnospirales_Dorea_Dorea formicigenerans_MB2bin120	Pathway module_ Nucleotide and amino acid metabolism_ Cofactor and vitamin biosynthesis_Glutathione biosynthesis, glutamate => glutathione	0,004	0,032	0,451
Bacteria_Firmicutes_A_Clostridia_Lachnospirales_Lachnospirales_Dorea_Dorea longicatena_MB2bin81	Pathway module_ Nucleotide and amino acid metabolism_ Cofactor and vitamin biosynthesis_Glutathione biosynthesis, glutamate => glutathione	0,009	0,051	0,414
Bacteria_Firmicutes_A_Clostridia_Oscillospirales_Oscillospiraceae_CAG-103_?_MB2bin101	Pathway module_ Nucleotide and amino acid metabolism_ Cofactor and vitamin biosynthesis_Glutathione biosynthesis, glutamate => glutathione	0,026	0,098	-0,356
Bacteria_Firmicutes_A_Clostridia_Oscillospirales_Oscillospiraceae_CAG-83_?_MB2bin179	Pathway module_ Nucleotide and amino acid metabolism_ Cofactor and vitamin biosynthesis_Glutathione biosynthesis, glutamate => glutathione	0,001	0,011	-0,507
Bacteria_Firmicutes_A_Clostridia_Oscillospirales_Oscillospiraceae_Oscillibacter_?_MB2bin147	Pathway module_ Nucleotide and amino acid metabolism_ Cofactor and vitamin biosynthesis_Glutathione	0,002	0,016	-0,490

	biosynthesis, glutamate => glutathione			
Bacteria_Firmicutes_A_Clostridia_Oscillospirales_Ruminococcaceae_Faecalibacterium_Faecalibacterium prausnitzii_A_MB2bin78	Pathway module_ Nucleotide and amino acid metabolism_ Cofactor and vitamin biosynthesis_Glutathione biosynthesis, glutamate => glutathione	0,018	0,077	0,376
Bacteria_Firmicutes_A_Clostridia_Oscillospirales_Ruminococcaceae_Faecalibacterium_Faecalibacterium prausnitzii_E_MGS00146	Pathway module_ Nucleotide and amino acid metabolism_ Cofactor and vitamin biosynthesis_Glutathione biosynthesis, glutamate => glutathione	0,018	0,077	-0,377
Bacteria_Firmicutes_A_Clostridia_Oscillospirales_Ruminococcaceae_Ruminiclostridium_E_Ruminiclostridium_E sp003512525_MB2bin228	Pathway module_ Nucleotide and amino acid metabolism_ Cofactor and vitamin biosynthesis_Glutathione biosynthesis, glutamate => glutathione	0,025	0,097	-0,358
Bacteria_Firmicutes_A_Clostridia_Oscillospirales_Ruminococcaceae_Ruminococcus_C_Ruminococcus_C sp000433635_MB2bin321	Pathway module_ Nucleotide and amino acid metabolism_ Cofactor and vitamin biosynthesis_Glutathione biosynthesis, glutamate => glutathione	0,011	0,058	0,403
Bacteria_Proteobacteria_Gammaproteobacteria_Burkholderiales_Burkholderiaceae_Dakarella_Dakarella massiliensis_specI_v3_Cluster3664	Pathway module_ Nucleotide and amino acid metabolism_ Cofactor and vitamin biosynthesis_Glutathione biosynthesis, glutamate => glutathione	0,014	0,065	-0,391
Bacteria_Proteobacteria_Gammaproteobacteria_Enterobacterales_Enterobacteriaceae_Escherichia_Escherichia flexneri_MB2bin181	Pathway module_ Nucleotide and amino acid metabolism_ Cofactor and vitamin biosynthesis_Glutathione biosynthesis, glutamate => glutathione	0,019	0,080	0,373
Bacteria_Actinobacteriota_Actinobacteria_Actinomycetales_Bifidobacteriaceae_Bifidobacterium_Bifidobacterium ruminantium_specI_v3_Cluster2702	Pathway module_ Nucleotide and amino acid metabolism_ Serine and threonine metabolism_Serine biosynthesis, glycerate-3P => serine	0,002	0,052	0,475
Bacteria_Actinobacteriota_Actinomycetia_Actinomycetales_Bifidobacteriaceae_Bifidobacterium_Bifidobacterium longum_MB2bin141	Pathway module_ Nucleotide and amino acid metabolism_ Serine and threonine metabolism_Serine biosynthesis, glycerate-3P => serine	0,006	0,088	0,430

Bacteria_Actinobacteriota_Coriobacteriia_Coriobacteriales_Eggerthellaceae_Adlercreutzia_Adlercreutzia celatus_A_MB2bin193	Pathway module_ Nucleotide and amino acid metabolism_ Serine and threonine metabolism_Serine biosynthesis, glycerate-3P => serine	0,007	0,088	-0,427
Bacteria_Bacteroidota_Bacteroidia_Bacteroidales_Bacteroidaceae_Bacteroides_Bacteroides eggerthii_MB2bin146	Pathway module_ Nucleotide and amino acid metabolism_ Serine and threonine metabolism_Serine biosynthesis, glycerate-3P => serine	0,007	0,088	-0,428
Bacteria_Bacteroidota_Bacteroidia_Bacteroidales_Bacteroidaceae_Bacteroides_Bacteroides ovatus_MB2bin110	Pathway module_ Nucleotide and amino acid metabolism_ Serine and threonine metabolism_Serine biosynthesis, glycerate-3P => serine	0,001	0,043	-0,506
Bacteria_Bacteroidota_Bacteroidia_Bacteroidales_Bacteroidaceae_Bacteroides_Bacteroides stercoris_MB2bin42	Pathway module_ Nucleotide and amino acid metabolism_ Serine and threonine metabolism_Serine biosynthesis, glycerate-3P => serine	0,001	0,030	-0,530
Bacteria_Bacteroidota_Bacteroidia_Bacteroidales_Bacteroidaceae_Bacteroides_Bacteroides uniformis_MB2bin1	Pathway module_ Nucleotide and amino acid metabolism_ Serine and threonine metabolism_Serine biosynthesis, glycerate-3P => serine	0,001	0,044	-0,497
Bacteria_Bacteroidota_Bacteroidia_Bacteroidales_Bacteroidaceae_Prevotella_Prevotella sp900313215_specI_v3_Cluster7696	Pathway module_ Nucleotide and amino acid metabolism_ Serine and threonine metabolism_Serine biosynthesis, glycerate-3P => serine	0,000	0,014	0,580
Bacteria_Bacteroidota_Bacteroidia_Bacteroidales_Barnesiellaceae_Barnesiella_Barnesiella intestinihominis_MB2bin112	Pathway module_ Nucleotide and amino acid metabolism_ Serine and threonine metabolism_Serine biosynthesis, glycerate-3P => serine	0,004	0,063	-0,456
Bacteria_Bacteroidota_Bacteroidia_Bacteroidales_Rikenellaceae_Alistipes_A_Alistipes_A ihumii_MB2bin204	Pathway module_ Nucleotide and amino acid metabolism_ Serine and threonine metabolism_Serine biosynthesis, glycerate-3P => serine	0,000	0,014	-0,567
Bacteria_Bacteroidota_Bacteroidia_Bacteroidales_Rikenellaceae_Alistipes_Alistipes putredinis_MB2bin34	Pathway module_ Nucleotide and amino acid metabolism_ Serine and threonine metabolism_Serine	0,002	0,052	-0,472

	biosynthesis, glycerate-3P => serine			
Bacteria_Bacteroidota_Bacteroidia_Bacteroidales_Tannerellaceae_Parabacteroides_Parabacteroides distasonis_MB2bin71	Pathway module_ Nucleotide and amino acid metabolism_ Serine and threonine metabolism_Serine biosynthesis, glycerate-3P => serine	0,004	0,063	-0,454
Bacteria_Firmicutes_A_Clostridia_A_Christensenellales_QAND01_UMGS1975_UMGS1975 sp900546685_MB2bin272	Pathway module_ Nucleotide and amino acid metabolism_ Serine and threonine metabolism_Serine biosynthesis, glycerate-3P => serine	0,002	0,052	0,479
Bacteria_Actinobacteriota_Actinobacteria_Actinomycetales_Bifidobacteriaceae_Bifidobacterium_Bifidobacterium ruminantium_specI_v3_Cluster2702	Pathway module_ Nucleotide and amino acid metabolism_ Serine and threonine metabolism_Threonine biosynthesis, aspartate => homoserine => threonine	0,000	0,000	0,704
Bacteria_Actinobacteriota_Actinomycetia_Actinomycetales_Bifidobacteriaceae_Bifidobacterium_Bifidobacterium adolescentis_MB2bin135	Pathway module_ Nucleotide and amino acid metabolism_ Serine and threonine metabolism_Threonine biosynthesis, aspartate => homoserine => threonine	0,000	0,000	0,670
Bacteria_Actinobacteriota_Actinomycetia_Actinomycetales_Bifidobacteriaceae_Bifidobacterium_Bifidobacterium angulatum_MB2bin288	Pathway module_ Nucleotide and amino acid metabolism_ Serine and threonine metabolism_Threonine biosynthesis, aspartate => homoserine => threonine	0,000	0,000	0,672
Bacteria_Actinobacteriota_Coriobacteriia_Coriobacteriales_Coriobacteriaceae_Collinsella_?_MB2bin75	Pathway module_ Nucleotide and amino acid metabolism_ Serine and threonine metabolism_Threonine biosynthesis, aspartate => homoserine => threonine	0,010	0,059	0,406
Bacteria_Actinobacteriota_Coriobacteriia_Coriobacteriales_Coriobacteriaceae_Collinsella_Collinsella sp000763055_MB2bin202	Pathway module_ Nucleotide and amino acid metabolism_ Serine and threonine metabolism_Threonine biosynthesis, aspartate => homoserine => threonine	0,005	0,044	0,438
Bacteria_Bacteroidota_Bacteroidia_Bacteroidales_Bacteroidaceae_Bacteroides_B_Bacteroides_B sartorii_specI_v3_Cluster2366	Pathway module_ Nucleotide and amino acid metabolism_ Serine and threonine metabolism_Threonine biosynthesis, aspartate => homoserine => threonine	0,000	0,004	-0,575

Bacteria_Bacteroidota_Bacteroidia_Bacteroidales_Bacteroidaceae_Bacteroides_Bacteroides fragilis_MB2bin178	Pathway module_ Nucleotide and amino acid metabolism_ Serine and threonine metabolism_Threonine biosynthesis, aspartate => homoserine => threonine	0,007	0,047	-0,423
Bacteria_Bacteroidota_Bacteroidia_Bacteroidales_Bacteroidaceae_Bacteroides_Bacteroides ovatus_MB2bin110	Pathway module_ Nucleotide and amino acid metabolism_ Serine and threonine metabolism_Threonine biosynthesis, aspartate => homoserine => threonine	0,007	0,047	-0,423
Bacteria_Bacteroidota_Bacteroidia_Bacteroidales_Bacteroidaceae_Bacteroides_Bacteroides thetaiotaomicron_MB2bin137	Pathway module_ Nucleotide and amino acid metabolism_ Serine and threonine metabolism_Threonine biosynthesis, aspartate => homoserine => threonine	0,000	0,008	-0,541
Bacteria_Bacteroidota_Bacteroidia_Bacteroidales_Bacteroidaceae_Phocaeicola_Phocaeicola vulgatus_MB2bin4	Pathway module_ Nucleotide and amino acid metabolism_ Serine and threonine metabolism_Threonine biosynthesis, aspartate => homoserine => threonine	0,000	0,004	-0,577
Bacteria_Bacteroidota_Bacteroidia_Bacteroidales_Barnesiellaceae_Barnesiella_Barnesiella intestinihominis_MB2bin112	Pathway module_ Nucleotide and amino acid metabolism_ Serine and threonine metabolism_Threonine biosynthesis, aspartate => homoserine => threonine	0,001	0,013	-0,511
Bacteria_Bacteroidota_Bacteroidia_Bacteroidales_Rikenellaceae_Alistipes_Alistipes finegoldii_MB2bin46	Pathway module_ Nucleotide and amino acid metabolism_ Serine and threonine metabolism_Threonine biosynthesis, aspartate => homoserine => threonine	0,007	0,047	-0,422
Bacteria_Bacteroidota_Bacteroidia_Bacteroidales_Rikenellaceae_Alistipes_Alistipes obesi_MB2bin28	Pathway module_ Nucleotide and amino acid metabolism_ Serine and threonine metabolism_Threonine biosynthesis, aspartate => homoserine => threonine	0,006	0,045	-0,432
Bacteria_Bacteroidota_Bacteroidia_Bacteroidales_Rikenellaceae_Alistipes_Alistipes onderdonkii_MB2bin22	Pathway module_ Nucleotide and amino acid metabolism_ Serine and threonine metabolism_Threonine biosynthesis, aspartate => homoserine => threonine	0,022	0,100	-0,366
Bacteria_Bacteroidota_Bacteroidia_Bacteroidales_Rikenellaceae_Alistipes_Alistipes shahii_MB2bin10	Pathway module_ Nucleotide and amino acid metabolism_ Serine and threonine metabolism_Threonine	0,007	0,047	-0,427

	biosynthesis, aspartate => homoserine => threonine			
Bacteria_Bacteroidota_Bacteroidia_Bacteroidales_Tannerellaceae_Parabacteroides_Parabacteroides johnsonii_MB2bin185	Pathway module_ Nucleotide and amino acid metabolism_ Serine and threonine metabolism_Threonine biosynthesis, aspartate => homoserine => threonine	0,020	0,095	-0,370
Bacteria_Firmicutes_A_Clostridia_A_Christensenellales_QAND01_UMGS1975_UMGS1975 sp900546685_MB2bin272	Pathway module_ Nucleotide and amino acid metabolism_ Serine and threonine metabolism_Threonine biosynthesis, aspartate => homoserine => threonine	0,009	0,054	0,412
Bacteria_Firmicutes_A_Clostridia_Lachnospirales_Lachnospiraceae_Anaerobutyricum_?_MB2bin124	Pathway module_ Nucleotide and amino acid metabolism_ Serine and threonine metabolism_Threonine biosynthesis, aspartate => homoserine => threonine	0,001	0,010	0,526
Bacteria_Firmicutes_A_Clostridia_Lachnospirales_Lachnospiraceae_Anaerobutyricum_Anaerobutyricum hallii_MB2bin3	Pathway module_ Nucleotide and amino acid metabolism_ Serine and threonine metabolism_Threonine biosynthesis, aspartate => homoserine => threonine	0,001	0,015	0,503
Bacteria_Firmicutes_A_Clostridia_Lachnospirales_Lachnospiraceae_Anaerostipes_Anaerostipes hadrus_A_specI_v3_Cluster856	Pathway module_ Nucleotide and amino acid metabolism_ Serine and threonine metabolism_Threonine biosynthesis, aspartate => homoserine => threonine	0,001	0,010	0,527
Bacteria_Firmicutes_A_Clostridia_Lachnospirales_Lachnospiraceae_Blautia_A_?_MB2bin14	Pathway module_ Nucleotide and amino acid metabolism_ Serine and threonine metabolism_Threonine biosynthesis, aspartate => homoserine => threonine	0,012	0,067	0,397
Bacteria_Firmicutes_A_Clostridia_Lachnospirales_Lachnospiraceae_Blautia_A_?_MB2bin175	Pathway module_ Nucleotide and amino acid metabolism_ Serine and threonine metabolism_Threonine biosynthesis, aspartate => homoserine => threonine	0,003	0,030	0,459
Bacteria_Firmicutes_A_Clostridia_Lachnospirales_Lachnospiraceae_Blautia_A_Blautia_A massiliensis_MB2bin79	Pathway module_ Nucleotide and amino acid metabolism_ Serine and threonine metabolism_Threonine biosynthesis, aspartate => homoserine => threonine	0,003	0,030	0,460

Bacteria_Firmicutes_A_Clostridia _Lachnospirales_Lachnospiraceae _Blautia_A_Blautia_A sp900066145_MB2bin111	Pathway module_ Nucleotide and amino acid metabolism_ Serine and threonine metabolism_Threonine biosynthesis, aspartate => homoserine => threonine	0,012	0,064	0,400
Bacteria_Firmicutes_A_Clostridia _Lachnospirales_Lachnospiraceae _Blautia_A_Blautia_A sp900066335_MB2bin25	Pathway module_ Nucleotide and amino acid metabolism_ Serine and threonine metabolism_Threonine biosynthesis, aspartate => homoserine => threonine	0,003	0,030	0,459
Bacteria_Firmicutes_A_Clostridia _Lachnospirales_Lachnospiraceae _Blautia_A_Blautia_A sp900548245_MB2bin70	Pathway module_ Nucleotide and amino acid metabolism_ Serine and threonine metabolism_Threonine biosynthesis, aspartate => homoserine => threonine	0,017	0,087	0,381
Bacteria_Firmicutes_A_Clostridia _Lachnospirales_Lachnospiraceae _Mediterraneibacter_Mediterran eibacter faecis_MB2bin127	Pathway module_ Nucleotide and amino acid metabolism_ Serine and threonine metabolism_Threonine biosynthesis, aspartate => homoserine => threonine	0,000	0,003	0,594
Bacteria_Firmicutes_A_Clostridia _Lachnospirales_Lachnospiraceae _Ruminococcus_A_Ruminococcus _A sp003011855_MB2bin82	Pathway module_ Nucleotide and amino acid metabolism_ Serine and threonine metabolism_Threonine biosynthesis, aspartate => homoserine => threonine	0,001	0,018	0,492
Bacteria_Firmicutes_A_Clostridia _Lachnospirales_Lachnospiraceae _UMGS1375_UMGS1375 sp900066615_MB2bin12	Pathway module_ Nucleotide and amino acid metabolism_ Serine and threonine metabolism_Threonine biosynthesis, aspartate => homoserine => threonine	0,001	0,011	0,520
Bacteria_Firmicutes_A_Clostridia _Lachnospirales_Lachnospirales_ Dorea_Dorea formicigenerans_MB2bin120	Pathway module_ Nucleotide and amino acid metabolism_ Serine and threonine metabolism_Threonine biosynthesis, aspartate => homoserine => threonine	0,004	0,032	0,453
Bacteria_Firmicutes_A_Clostridia _Lachnospirales_Lachnospirales_ Dorea_Dorea longicatena_MB2bin81	Pathway module_ Nucleotide and amino acid metabolism_ Serine and threonine metabolism_Threonine biosynthesis, aspartate => homoserine => threonine	0,006	0,044	0,436
Bacteria_Firmicutes_A_Clostridia _Oscillospirales_Oscillospiraceae _CAG-83_?_MB2bin179	Pathway module_ Nucleotide and amino acid metabolism_ Serine and threonine metabolism_Threonine	0,019	0,091	-0,374

	biosynthesis, aspartate => homoserine => threonine			
Bacteria_Firmicutes_A_Clostridia_Oscillospirales_Oscillospiraceae_Oscillibacter_?_MB2bin147	Pathway module_ Nucleotide and amino acid metabolism_ Serine and threonine metabolism_Threonine biosynthesis, aspartate => homoserine => threonine	0,019	0,091	-0,374
Bacteria_Firmicutes_A_Clostridia_Oscillospirales_Ruminococcaceae_Gemmiger_Gemmiger sp900539695_MB2bin39	Pathway module_ Nucleotide and amino acid metabolism_ Serine and threonine metabolism_Threonine biosynthesis, aspartate => homoserine => threonine	0,008	0,047	0,421
Bacteria_Firmicutes_A_Clostridia_Peptostreptococcales_Peptostreptococcaceae_Romboutsia_Romboutsia lituseburensis_specI_v3_Cluster6795	Pathway module_ Nucleotide and amino acid metabolism_ Serine and threonine metabolism_Threonine biosynthesis, aspartate => homoserine => threonine	0,000	0,004	0,572
Bacteria_Firmicutes_C_Negativicutes_Veillonellales_Dialisteraceae_Dialister_Dialister succinatiphilus_specI_v3_Cluster11863	Pathway module_ Nucleotide and amino acid metabolism_ Serine and threonine metabolism_Threonine biosynthesis, aspartate => homoserine => threonine	0,003	0,030	0,459
Bacteria_Firmicutes_I_Bacilli_A_Lactobacillales_Streptococcaceae_Streptococcus_?_MB2bin209	Pathway module_ Nucleotide and amino acid metabolism_ Serine and threonine metabolism_Threonine biosynthesis, aspartate => homoserine => threonine	0,003	0,030	0,467
Bacteria_Firmicutes_I_Bacilli_A_Lactobacillales_Streptococcaceae_Streptococcus_Streptococcus thermophilus_MB2bin145	Pathway module_ Nucleotide and amino acid metabolism_ Serine and threonine metabolism_Threonine biosynthesis, aspartate => homoserine => threonine	0,018	0,091	0,376
Bacteria_Actinobacteriota_Coriobacteriia_Coriobacteriales_Eggerthellaceae_Adlercreutzia_Adlercreutzia celatus_A_MB2bin193	Signature module_ Gene set_ Metabolic capacity_Sulfate-sulfur assimilation	0,000	0,006	0,582
Bacteria_Bacteroidota_Bacteroidia_Bacteroidales_Bacteroidaceae_Bacteroides_Bacteroides caccae_MB2bin142	Signature module_ Gene set_ Metabolic capacity_Sulfate-sulfur assimilation	0,004	0,077	0,450
Bacteria_Bacteroidota_Bacteroidia_Bacteroidales_Bacteroidaceae_Bacteroides_Bacteroides caccae_specI_v3_Cluster3473	Signature module_ Gene set_ Metabolic capacity_Sulfate-sulfur assimilation	0,003	0,072	0,464

Bacteria_Firmicutes_A_Clostridia_Lachnospirales_Lachnospiraceae_Blautia_A_?_MB2bin9	Signature module_ Gene set_ Metabolic capacity_Sulfate-sulfur assimilation	0,007	0,097	0,423
Bacteria_Firmicutes_A_Clostridia_Lachnospirales_Lachnospiraceae_Eubacterium_I_Eubacterium_I ramulus_MB2bin11	Signature module_ Gene set_ Metabolic capacity_Sulfate-sulfur assimilation	0,000	0,004	0,607
Bacteria_Firmicutes_A_Clostridia_Lachnospirales_Lachnospiraceae_Eubacterium_I_Eubacterium_I sp900546495_MB2bin174	Signature module_ Gene set_ Metabolic capacity_Sulfate-sulfur assimilation	0,000	0,001	0,652
Bacteria_Firmicutes_A_Clostridia_Lachnospirales_Lachnospiraceae_Roseburia_Roseburia sp900552665_MB2bin35	Signature module_ Gene set_ Metabolic capacity_Sulfate-sulfur assimilation	0,002	0,056	0,488
Bacteria_Firmicutes_A_Clostridia_Monoglobales_A_UBA1381_CAG-41_?_MB2bin97	Signature module_ Gene set_ Metabolic capacity_Sulfate-sulfur assimilation	0,000	0,015	0,543
Bacteria_Firmicutes_A_Clostridia_Oscillospirales_Butyricicoccaceae_Agathobaculum_Agathobaculum butyriciproducens_MB2bin73	Signature module_ Gene set_ Metabolic capacity_Sulfate-sulfur assimilation	0,003	0,072	0,458
Bacteria_Firmicutes_A_Clostridia_Oscillospirales_Oscillospiraceae_CAG-170_CAG-170 sp900545925_MB2bin33	Signature module_ Gene set_ Metabolic capacity_Sulfate-sulfur assimilation	0,005	0,081	-0,440
Bacteria_Firmicutes_A_Clostridia_Oscillospirales_Ruminococcaceae_Faecalibacterium_Faecalibacterium prausnitzii_G_MB2bin45	Signature module_ Gene set_ Metabolic capacity_Sulfate-sulfur assimilation	0,005	0,081	0,439
Bacteria_Firmicutes_A_Clostridia_Oscillospirales_Ruminococcaceae_Ruminiclostridium_E_Ruminiclostridium_E siraeum_MB2bin52	Signature module_ Gene set_ Metabolic capacity_Sulfate-sulfur assimilation	0,003	0,072	-0,459
Bacteria_Firmicutes_A_Clostridia_Oscillospirales_Ruminococcaceae_Ruminiclostridium_E_Ruminiclostridium_E sp003512525_MB2bin228	Signature module_ Gene set_ Metabolic capacity_Sulfate-sulfur assimilation	0,006	0,083	-0,434
Bacteria_Actinobacteriota_Actinobacteria_Actinomycetales_Bifidobacteriaceae_Bifidobacterium_Bifidobacterium ruminantium_specI_v3_Cluster2702	Structural complex_ Environmental information processing_ ABC-2 type and other transport systems_Cell division transport system	0,000	0,006	0,612
Bacteria_Actinobacteriota_Actinomycetia_Actinomycetales_Bifidobacteriaceae_Bifidobacterium_Bifidobacterium adolescentis_MB2bin135	Structural complex_ Environmental information processing_ ABC-2 type and other transport systems_Cell division transport system	0,000	0,017	0,532

Bacteria_Actinobacteriota_Actinomycetia_Actinomycetales_Bifidobacteriaceae_Bifidobacterium_Bifidobacterium angulatum_MB2bin288	Structural complex_ Environmental information processing_ ABC-2 type and other transport systems_Cell division transport system	0,000	0,017	0,537
Bacteria_Actinobacteriota_Coriobacteriia_Coriobacteriales_Coriobacteriaceae_Collinsella_Collinsella aerofaciens_F_specI_v3_Cluster3625	Structural complex_ Environmental information processing_ ABC-2 type and other transport systems_Cell division transport system	0,002	0,028	0,488
Bacteria_Actinobacteriota_Coriobacteriia_Coriobacteriales_Eggerthellaceae_Adlercreutzia_Adlercreutzia celatus_A_MB2bin193	Structural complex_ Environmental information processing_ ABC-2 type and other transport systems_Cell division transport system	0,000	0,016	-0,555
Bacteria_Bacteroidota_Bacteroidia_Bacteroidales_Bacteroidaceae_Bacteroides_Bacteroides eggerthii_MB2bin146	Structural complex_ Environmental information processing_ ABC-2 type and other transport systems_Cell division transport system	0,004	0,064	-0,449
Bacteria_Bacteroidota_Bacteroidia_Bacteroidales_Bacteroidaceae_Bacteroides_Bacteroides fragilis_MB2bin178	Structural complex_ Environmental information processing_ ABC-2 type and other transport systems_Cell division transport system	0,001	0,023	-0,499
Bacteria_Bacteroidota_Bacteroidia_Bacteroidales_Bacteroidaceae_Prevotella_?_MB2bin51	Structural complex_ Environmental information processing_ ABC-2 type and other transport systems_Cell division transport system	0,001	0,023	0,500
Bacteria_Bacteroidota_Bacteroidia_Bacteroidales_Barnesiellaceae_Barnesiella_Barnesiella intestinihominis_MB2bin112	Structural complex_ Environmental information processing_ ABC-2 type and other transport systems_Cell division transport system	0,001	0,023	-0,505
Bacteria_Firmicutes_A_Clostridia_Lachnospirales_Lachnospiraceae_Blautia_A_?_MB2bin9	Structural complex_ Environmental information processing_ ABC-2 type and other transport systems_Cell division transport system	0,000	0,016	-0,551
Bacteria_Firmicutes_A_Clostridia_Lachnospirales_Lachnospiraceae_Coprococcus_A_Coprococcus_A catus_MB2bin38	Structural complex_ Environmental information processing_ ABC-2 type and other transport systems_Cell division transport system	0,005	0,071	0,441
Bacteria_Firmicutes_A_Clostridia_Oscillospirales_Ruminococcaceae_Angelakisella_Angelakisella sp900547385_MB2bin279	Structural complex_ Environmental information processing_ ABC-2 type and other transport systems_Cell division transport system	0,001	0,019	0,521

Bacteria_Actinobacteriota_Actinobacteria_Actinomycetales_Bifidobacteriaceae_Bifidobacterium_Bifidobacterium ruminantium_specI_v3_Cluster2702	Structural complex_ Environmental information processing_ Metallic cation, iron-siderophore and vitamin B12 transport system_Iron complex transport system	0,002	0,065	-0,481
Bacteria_Actinobacteriota_Actinomycetia_Actinomycetales_Bifidobacteriaceae_Bifidobacterium_Bifidobacterium adolescentis_MB2bin135	Structural complex_ Environmental information processing_ Metallic cation, iron-siderophore and vitamin B12 transport system_Iron complex transport system	0,006	0,073	-0,434
Bacteria_Actinobacteriota_Actinomycetia_Actinomycetales_Bifidobacteriaceae_Bifidobacterium_Bifidobacterium angulatum_MB2bin288	Structural complex_ Environmental information processing_ Metallic cation, iron-siderophore and vitamin B12 transport system_Iron complex transport system	0,005	0,073	-0,438
Bacteria_Actinobacteriota_Actinomycetia_Actinomycetales_Bifidobacteriaceae_Bifidobacterium_Bifidobacterium bifidum_MB2bin203	Structural complex_ Environmental information processing_ Metallic cation, iron-siderophore and vitamin B12 transport system_Iron complex transport system	0,005	0,070	-0,445
Bacteria_Actinobacteriota_Actinomycetia_Actinomycetales_Bifidobacteriaceae_Bifidobacterium_Bifidobacterium longum_MB2bin141	Structural complex_ Environmental information processing_ Metallic cation, iron-siderophore and vitamin B12 transport system_Iron complex transport system	0,004	0,070	-0,446
Bacteria_Bacteroidota_Bacteroidia_Bacteroidales_Bacteroidaceae_Prevotella_?_MB2bin51	Structural complex_ Environmental information processing_ Metallic cation, iron-siderophore and vitamin B12 transport system_Iron complex transport system	0,006	0,073	-0,433
Bacteria_Bacteroidota_Bacteroidia_Bacteroidales_Bacteroidaceae_Prevotella_Prevotella copri_A_MB2bin109	Structural complex_ Environmental information processing_ Metallic cation, iron-siderophore and vitamin B12 transport system_Iron complex transport system	0,009	0,095	-0,414
Bacteria_Bacteroidota_Bacteroidia_Bacteroidales_Rikenellaceae_Alistipes_Alistipes finegoldii_MB2bin46	Structural complex_ Environmental information processing_ Metallic cation, iron-siderophore and vitamin B12 transport system_Iron complex transport system	0,003	0,065	0,458
Bacteria_Firmicutes_A_Clostridia_A_Christensenellales_QAND01_UMGS1975_UMGS1975 sp900546685_MB2bin272	Structural complex_ Environmental information processing_ Metallic cation, iron-siderophore and vitamin	0,003	0,065	-0,463

	B12 transport system_Iron complex transport system			
Bacteria_Firmicutes_A_Clostridia_Lachnospirales_Lachnospiraceae_Anaerostipes_Anaerostipes hadrus_MB2bin47	Structural complex_ Environmental information processing_ Metallic cation, iron-siderophore and vitamin B12 transport system_Iron complex transport system	0,001	0,065	-0,496
Bacteria_Firmicutes_A_Clostridia_Lachnospirales_Lachnospiraceae_CAG-127_CAG-127 sp900319515_MB2bin98	Structural complex_ Environmental information processing_ Metallic cation, iron-siderophore and vitamin B12 transport system_Iron complex transport system	0,003	0,065	0,457
Bacteria_Firmicutes_A_Clostridia_Oscillospirales_Acutalibacteraceae_CAG-177_CAG-177 sp003514385_MB2bin37	Structural complex_ Environmental information processing_ Metallic cation, iron-siderophore and vitamin B12 transport system_Iron complex transport system	0,003	0,065	-0,468
Bacteria_Firmicutes_A_Clostridia_Oscillospirales_Acutalibacteraceae_CAG-177_CAG-177 sp003538135_MB2bin156	Structural complex_ Environmental information processing_ Metallic cation, iron-siderophore and vitamin B12 transport system_Iron complex transport system	0,001	0,065	-0,506
Bacteria_Firmicutes_A_Clostridia_Oscillospirales_Acutalibacteraceae_Ruminococcus_E_Ruminococcus_E sp003526955_MB2bin130	Structural complex_ Environmental information processing_ Metallic cation, iron-siderophore and vitamin B12 transport system_Iron complex transport system	0,009	0,095	0,414
Bacteria_Firmicutes_A_Clostridia_Oscillospirales_Ruminococcaceae_Faecalibacterium_Faecalibacterium prausnitzii_E_MGS00146	Structural complex_ Environmental information processing_ Metallic cation, iron-siderophore and vitamin B12 transport system_Iron complex transport system	0,010	0,097	0,410
Bacteria_Firmicutes_C_Negativicutes_Veillonellales_Dialisteraceae_Dialister_Dialister succinatiphilus_specI_v3_Cluster 11863	Structural complex_ Environmental information processing_ Metallic cation, iron-siderophore and vitamin B12 transport system_Iron complex transport system	0,002	0,065	-0,474
Bacteria_Proteobacteria_Gammaproteobacteria_Enterobacterales_Enterobacteriaceae_Klebsiella_B_Klebsiella_B aerogenes_specI_v3_Cluster84	Structural complex_ Environmental information processing_ Metallic cation, iron-siderophore and vitamin B12 transport system_Iron complex transport system	0,002	0,065	0,476

Bacteria_Actinobacteriota_Actinomycetia_Actinomycetales_Bifidobacteriaceae_Bifidobacterium_Bifidobacterium adolescentis_MB2bin135	Structural complex_ Environmental information processing_ Peptide and nickel transport system_Peptides/nickel transport system	0,004	0,087	0,456
Bacteria_Actinobacteriota_Actinomycetia_Actinomycetales_Bifidobacteriaceae_Bifidobacterium_Bifidobacterium longum_MB2bin141	Structural complex_ Environmental information processing_ Peptide and nickel transport system_Peptides/nickel transport system	0,003	0,084	0,468
Bacteria_Bacteroidota_Bacteroidia_Bacteroidales_Barnesiellaceae_Barnesiella_Barnesiella intestinihominis_MB2bin112	Structural complex_ Environmental information processing_ Peptide and nickel transport system_Peptides/nickel transport system	0,001	0,050	-0,501
Bacteria_Bacteroidota_Bacteroidia_Bacteroidales_Rikenellaceae_Alistipes_Alistipes obesi_MB2bin28	Structural complex_ Environmental information processing_ Peptide and nickel transport system_Peptides/nickel transport system	0,003	0,084	-0,464
Bacteria_Firmicutes_A_Clostridia_Lachnospirales_Lachnospiraceae_Anaerostipes_Anaerostipes hadrus_MB2bin47	Structural complex_ Environmental information processing_ Peptide and nickel transport system_Peptides/nickel transport system	0,001	0,050	0,504
Bacteria_Firmicutes_A_Clostridia_Oscillospirales_Oscillospiraceae_CAG-83_?_MB2bin179	Structural complex_ Environmental information processing_ Peptide and nickel transport system_Peptides/nickel transport system	0,000	0,026	-0,549
Bacteria_Firmicutes_A_Clostridia_Oscillospirales_Oscillospiraceae_Oscillibacter_?_MB2bin147	Structural complex_ Environmental information processing_ Peptide and nickel transport system_Peptides/nickel transport system	0,000	0,026	-0,553
Bacteria_Actinobacteriota_Actinobacteria_Actinomycetales_Bifidobacteriaceae_Bifidobacterium_Bifidobacterium ruminantium_specI_v3_Cluster2702	Structural complex_ Environmental information processing_ Phosphate and amino acid transport system_Branched-chain amino acid transport system	0,002	0,052	0,472
Bacteria_Actinobacteriota_Actinomycetia_Actinomycetales_Bifidobacteriaceae_Bifidobacterium_Bif	Structural complex_ Environmental information processing_ Phosphate and amino acid transport	0,002	0,052	0,474

idobacterium adolescentis_MB2bin135	system_Branched-chain amino acid transport system			
Bacteria_Actinobacteriota_Actinomycetia_Actinomycetales_Bifidobacteriaceae_Bifidobacterium_Bifidobacterium angulatum_MB2bin288	Structural complex_ Environmental information processing_ Phosphate and amino acid transport system_Branched-chain amino acid transport system	0,004	0,062	0,455
Bacteria_Actinobacteriota_Coriobacteriia_Coriobacteriales_Eggerthellaceae_Adlercreutzia_Adlercreutzia celatus_A_MB2bin193	Structural complex_ Environmental information processing_ Phosphate and amino acid transport system_Branched-chain amino acid transport system	0,000	0,017	-0,556
Bacteria_Bacteroidota_Bacteroidia_Bacteroidales_Bacteroidaceae_Bacteroides_Bacteroides fragilis_MB2bin178	Structural complex_ Environmental information processing_ Phosphate and amino acid transport system_Branched-chain amino acid transport system	0,001	0,036	-0,498
Bacteria_Firmicutes_A_Clostridia_A_Christensenellales_CAG-138_SFEL01_SFEL01 sp004557245_MB2bin134	Structural complex_ Environmental information processing_ Phosphate and amino acid transport system_Branched-chain amino acid transport system	0,001	0,036	0,505
Bacteria_Firmicutes_A_Clostridia_Lachnospirales_Lachnospiraceae_Blautia_A_?_MB2bin9	Structural complex_ Environmental information processing_ Phosphate and amino acid transport system_Branched-chain amino acid transport system	0,000	0,017	-0,549
Bacteria_Firmicutes_A_Clostridia_Oscillospirales_Oscillospiraceae_CAG-83_?_MB2bin100	Structural complex_ Environmental information processing_ Phosphate and amino acid transport system_Branched-chain amino acid transport system	0,003	0,057	0,463
Bacteria_Firmicutes_A_Clostridia_Oscillospirales_Oscillospiraceae_CAG-83_?_MB2bin108	Structural complex_ Environmental information processing_ Phosphate and amino acid transport system_Branched-chain amino acid transport system	0,000	0,006	0,614
Bacteria_Firmicutes_A_Clostridia_Oscillospirales_Oscillospiraceae_ER4_?_MB2bin17	Structural complex_ Environmental information processing_ Phosphate and amino acid transport system_Branched-chain amino acid transport system	0,001	0,036	0,505

Bacteria_Actinobacteriota_Actinobacteria_Actinomycetales_Bifidobacteriaceae_Bifidobacterium_Bifidobacterium ruminantium_specI_v3_Cluster2702	Structural complex_ Environmental information processing_ Phosphate and amino acid transport system_Putative polar amino acid transport system	0,000	0,001	0,655
Bacteria_Actinobacteriota_Actinomycetia_Actinomycetales_Bifidobacteriaceae_Bifidobacterium_Bifidobacterium adolescentis_MB2bin135	Structural complex_ Environmental information processing_ Phosphate and amino acid transport system_Putative polar amino acid transport system	0,000	0,001	0,624
Bacteria_Actinobacteriota_Actinomycetia_Actinomycetales_Bifidobacteriaceae_Bifidobacterium_Bifidobacterium angulatum_MB2bin288	Structural complex_ Environmental information processing_ Phosphate and amino acid transport system_Putative polar amino acid transport system	0,000	0,006	0,557
Bacteria_Actinobacteriota_Coriobacteriia_Coriobacteriales_Coriobacteriaceae_Collinsella_?_MB2bin75	Structural complex_ Environmental information processing_ Phosphate and amino acid transport system_Putative polar amino acid transport system	0,004	0,035	0,452
Bacteria_Actinobacteriota_Coriobacteriia_Coriobacteriales_Coriobacteriaceae_Collinsella_Collinsella sp000763055_MB2bin202	Structural complex_ Environmental information processing_ Phosphate and amino acid transport system_Putative polar amino acid transport system	0,003	0,026	0,467
Bacteria_Bacteroidota_Bacteroidia_Bacteroidales_Bacteroidaceae_Bacteroides_B_Bacteroides_B sartorii_specI_v3_Cluster2366	Structural complex_ Environmental information processing_ Phosphate and amino acid transport system_Putative polar amino acid transport system	0,000	0,001	-0,617
Bacteria_Bacteroidota_Bacteroidia_Bacteroidales_Bacteroidaceae_Bacteroides_Bacteroides eggerthii_MB2bin146	Structural complex_ Environmental information processing_ Phosphate and amino acid transport system_Putative polar amino acid transport system	0,002	0,019	-0,484
Bacteria_Bacteroidota_Bacteroidia_Bacteroidales_Bacteroidaceae_Bacteroides_Bacteroides ovatus_MB2bin110	Structural complex_ Environmental information processing_ Phosphate and amino acid transport system_Putative polar amino acid transport system	0,001	0,013	-0,512
Bacteria_Bacteroidota_Bacteroidia_Bacteroidales_Bacteroidaceae_Bacteroides_Bacteroides stercoris_MB2bin42	Structural complex_ Environmental information processing_ Phosphate and amino acid transport	0,008	0,061	-0,419

	system_Putative polar amino acid transport system			
Bacteria_Bacteroidota_Bacteroidia_Bacteroidales_Bacteroidaceae_Bacteroides_Bacteroides thetaiotaomicron_MB2bin137	Structural complex_ Environmental information processing_ Phosphate and amino acid transport system_Putative polar amino acid transport system	0,002	0,019	-0,487
Bacteria_Bacteroidota_Bacteroidia_Bacteroidales_Bacteroidaceae_Phocaeicola_Phocaeicola vulgatus_MB2bin4	Structural complex_ Environmental information processing_ Phosphate and amino acid transport system_Putative polar amino acid transport system	0,000	0,001	-0,611
Bacteria_Bacteroidota_Bacteroidia_Bacteroidales_Bacteroidaceae_Prevotella_?_MB2bin51	Structural complex_ Environmental information processing_ Phosphate and amino acid transport system_Putative polar amino acid transport system	0,015	0,081	0,387
Bacteria_Bacteroidota_Bacteroidia_Bacteroidales_Barnesiellaceae_Barnesiella_Barnesiella intestinihominis_MB2bin112	Structural complex_ Environmental information processing_ Phosphate and amino acid transport system_Putative polar amino acid transport system	0,001	0,015	-0,504
Bacteria_Bacteroidota_Bacteroidia_Bacteroidales_Rikenellaceae_Alistipes_Alistipes obesi_MB2bin28	Structural complex_ Environmental information processing_ Phosphate and amino acid transport system_Putative polar amino acid transport system	0,011	0,076	-0,401
Bacteria_Bacteroidota_Bacteroidia_Bacteroidales_Rikenellaceae_Alistipes_Alistipes onderdonkii_MB2bin22	Structural complex_ Environmental information processing_ Phosphate and amino acid transport system_Putative polar amino acid transport system	0,015	0,081	-0,387
Bacteria_Bacteroidota_Bacteroidia_Bacteroidales_Rikenellaceae_Alistipes_Alistipes shahii_MB2bin10	Structural complex_ Environmental information processing_ Phosphate and amino acid transport system_Putative polar amino acid transport system	0,000	0,010	-0,533
Bacteria_Bacteroidota_Bacteroidia_Bacteroidales_Rikenellaceae_Alistipes_Alistipes sp900083545_specI_v3_Cluster1016	Structural complex_ Environmental information processing_ Phosphate and amino acid transport system_Putative polar amino acid transport system	0,004	0,038	-0,446

Bacteria_Firmicutes_A_Clostridia_A_Christensenellales_QAND01_UMGS1975_UMGS1975 sp900546685_MB2bin272	Structural complex_ Environmental information processing_ Phosphate and amino acid transport system_Putative polar amino acid transport system	0,014	0,081	0,392
Bacteria_Firmicutes_A_Clostridia_Lachnospirales_Lachnospiraceae_Blautia_A_?_MB2bin175	Structural complex_ Environmental information processing_ Phosphate and amino acid transport system_Putative polar amino acid transport system	0,011	0,076	0,402
Bacteria_Firmicutes_A_Clostridia_Lachnospirales_Lachnospiraceae_Mediterraneibacter_Mediterraneibacter faecis_MB2bin127	Structural complex_ Environmental information processing_ Phosphate and amino acid transport system_Putative polar amino acid transport system	0,000	0,001	0,603
Bacteria_Firmicutes_A_Clostridia_Lachnospirales_Lachnospiraceae_Ruminococcus_A_Ruminococcus_A sp003011855_MB2bin82	Structural complex_ Environmental information processing_ Phosphate and amino acid transport system_Putative polar amino acid transport system	0,008	0,061	0,418
Bacteria_Firmicutes_A_Clostridia_Lachnospirales_Lachnospiraceae_UMGS1375_UMGS1375 sp900066615_MB2bin12	Structural complex_ Environmental information processing_ Phosphate and amino acid transport system_Putative polar amino acid transport system	0,002	0,019	0,490
Bacteria_Firmicutes_A_Clostridia_Lachnospirales_Lachnospirales_Dorea_Dorea formicigenerans_MB2bin120	Structural complex_ Environmental information processing_ Phosphate and amino acid transport system_Putative polar amino acid transport system	0,015	0,081	0,387
Bacteria_Firmicutes_A_Clostridia_Oscillospirales_Oscillospiraceae_CAG-103_?_MB2bin101	Structural complex_ Environmental information processing_ Phosphate and amino acid transport system_Putative polar amino acid transport system	0,019	0,097	-0,375
Bacteria_Firmicutes_A_Clostridia_Oscillospirales_Oscillospiraceae_CAG-83_?_MB2bin108	Structural complex_ Environmental information processing_ Phosphate and amino acid transport system_Putative polar amino acid transport system	0,001	0,010	0,530
Bacteria_Firmicutes_A_Clostridia_Oscillospirales_Oscillospiraceae_CAG-83_?_MB2bin179	Structural complex_ Environmental information processing_ Phosphate and amino acid transport	0,013	0,081	-0,393

	system_Putative polar amino acid transport system			
Bacteria_Firmicutes_A_Clostridia_Oscillospirales_Ruminococcaceae_Gemmiger_Gemmiger sp900539695_MB2bin39	Structural complex_ Environmental information processing_ Phosphate and amino acid transport system_Putative polar amino acid transport system	0,011	0,076	0,403
Bacteria_Firmicutes_A_Clostridia_Oscillospirales_Ruminococcaceae_Gemmiger_Gemmiger sp900540595_MB2bin67	Structural complex_ Environmental information processing_ Phosphate and amino acid transport system_Putative polar amino acid transport system	0,015	0,081	0,388
Bacteria_Firmicutes_A_Clostridia_Peptostreptococcales_Peptostreptococcaceae_Romboutsia_Romboutsia lituseburensis_specI_v3_Cluster6795	Structural complex_ Environmental information processing_ Phosphate and amino acid transport system_Putative polar amino acid transport system	0,007	0,056	0,426
Bacteria_Firmicutes_C_Negativicutes_Veillonellales_Dialisteraceae_Dialister_Dialister succinatiphilus_specI_v3_Cluster11863	Structural complex_ Environmental information processing_ Phosphate and amino acid transport system_Putative polar amino acid transport system	0,001	0,010	0,525
Bacteria_Firmicutes_I_Bacilli_A_Lactobacillales_Streptococcaceae_Streptococcus_?_MB2bin209	Structural complex_ Environmental information processing_ Phosphate and amino acid transport system_Putative polar amino acid transport system	0,000	0,001	0,604
Bacteria_Firmicutes_I_Bacilli_A_Lactobacillales_Streptococcaceae_Streptococcus_Streptococcus sp000187445_specI_v3_Cluster1349	Structural complex_ Environmental information processing_ Phosphate and amino acid transport system_Putative polar amino acid transport system	0,002	0,019	0,488
Bacteria_Firmicutes_I_Bacilli_A_Lactobacillales_Streptococcaceae_Streptococcus_Streptococcus thermophilus_MB2bin145	Structural complex_ Environmental information processing_ Phosphate and amino acid transport system_Putative polar amino acid transport system	0,003	0,026	0,466
Bacteria_Actinobacteriota_Actinobacteria_Actinomycetales_Bifidobacteriaceae_Bifidobacterium_Bifidobacterium ruminantium_specI_v3_Cluster2702	Structural complex_ Genetic information processing_ DNA polymerase_DNA polymerase III complex, bacteria	0,004	0,042	0,454

Bacteria_Actinobacteriota_Actinomycetia_Actinomycetales_Bifidobacteriaceae_Bifidobacterium_Bifidobacterium adolescentis_MB2bin135	Structural complex_ Genetic information processing_ DNA polymerase_DNA polymerase III complex, bacteria	0,011	0,081	0,402
Bacteria_Actinobacteriota_Actinomycetia_Actinomycetales_Bifidobacteriaceae_Bifidobacterium_Bifidobacterium angulatum_MB2bin288	Structural complex_ Genetic information processing_ DNA polymerase_DNA polymerase III complex, bacteria	0,003	0,039	0,470
Bacteria_Actinobacteriota_Coriobacteriia_Coriobacteriales_Eggerthellaceae_Adlercreutzia_Adlercreutzia celatus_A_MB2bin193	Structural complex_ Genetic information processing_ DNA polymerase_DNA polymerase III complex, bacteria	0,002	0,039	-0,477
Bacteria_Bacteroidota_Bacteroidia_Bacteroidales_Bacteroidaceae_Bacteroides_B_Bacteroides_B sartorii_specI_v3_Cluster2366	Structural complex_ Genetic information processing_ DNA polymerase_DNA polymerase III complex, bacteria	0,002	0,039	-0,483
Bacteria_Bacteroidota_Bacteroidia_Bacteroidales_Bacteroidaceae_Bacteroides_Bacteroides eggerthii_MB2bin146	Structural complex_ Genetic information processing_ DNA polymerase_DNA polymerase III complex, bacteria	0,007	0,062	-0,424
Bacteria_Bacteroidota_Bacteroidia_Bacteroidales_Bacteroidaceae_Bacteroides_Bacteroides fragilis_MB2bin178	Structural complex_ Genetic information processing_ DNA polymerase_DNA polymerase III complex, bacteria	0,002	0,039	-0,486
Bacteria_Bacteroidota_Bacteroidia_Bacteroidales_Bacteroidaceae_Bacteroides_Bacteroides ovatus_MB2bin110	Structural complex_ Genetic information processing_ DNA polymerase_DNA polymerase III complex, bacteria	0,003	0,039	-0,460
Bacteria_Bacteroidota_Bacteroidia_Bacteroidales_Bacteroidaceae_Bacteroides_Bacteroides stercoris_MB2bin42	Structural complex_ Genetic information processing_ DNA polymerase_DNA polymerase III complex, bacteria	0,001	0,024	-0,523
Bacteria_Bacteroidota_Bacteroidia_Bacteroidales_Bacteroidaceae_Bacteroides_Bacteroides thetaiotaomicron_MB2bin137	Structural complex_ Genetic information processing_ DNA polymerase_DNA polymerase III complex, bacteria	0,000	0,024	-0,537
Bacteria_Bacteroidota_Bacteroidia_Bacteroidales_Bacteroidaceae_Phocaeicola_Phocaeicola vulgatus_MB2bin4	Structural complex_ Genetic information processing_ DNA polymerase_DNA polymerase III complex, bacteria	0,000	0,024	-0,551
Bacteria_Bacteroidota_Bacteroidia_Bacteroidales_Bacteroidaceae_Prevotella_?_MB2bin51	Structural complex_ Genetic information processing_ DNA polymerase_DNA polymerase III complex, bacteria	0,001	0,039	0,491
Bacteria_Bacteroidota_Bacteroidia_Bacteroidales_Bacteroidaceae_Prevotella_Prevotella copri_A_MB2bin109	Structural complex_ Genetic information processing_ DNA polymerase_DNA polymerase III complex, bacteria	0,008	0,062	0,421
Bacteria_Bacteroidota_Bacteroidia_Bacteroidales_Barnesiellaceae	Structural complex_ Genetic information processing_ DNA	0,000	0,024	-0,567

_Barnesiella_Barnesiella intestinihominis_MB2bin112	polymerase_DNA polymerase III complex, bacteria			
Bacteria_Bacteroidota_Bacteroidia_Bacteroidales_Rikenellaceae_Alistipes_A_Alistipes_A ihumii_MB2bin204	Structural complex_ Genetic information processing_ DNA polymerase_DNA polymerase III complex, bacteria	0,006	0,062	-0,429
Bacteria_Bacteroidota_Bacteroidia_Bacteroidales_Rikenellaceae_Alistipes_Alistipes obesi_MB2bin28	Structural complex_ Genetic information processing_ DNA polymerase_DNA polymerase III complex, bacteria	0,006	0,060	-0,433
Bacteria_Bacteroidota_Bacteroidia_Bacteroidales_Rikenellaceae_Alistipes_Alistipes onderdonkii_MB2bin22	Structural complex_ Genetic information processing_ DNA polymerase_DNA polymerase III complex, bacteria	0,004	0,047	-0,447
Bacteria_Firmicutes_A_Clostridia_Lachnospirales_Lachnospiraceae_Anaerostipes_Anaerostipes hadrus_A_specI_v3_Cluster856	Structural complex_ Genetic information processing_ DNA polymerase_DNA polymerase III complex, bacteria	0,003	0,039	0,464
Bacteria_Firmicutes_A_Clostridia_Lachnospirales_Lachnospiraceae_Blautia_A_?_MB2bin9	Structural complex_ Genetic information processing_ DNA polymerase_DNA polymerase III complex, bacteria	0,009	0,070	-0,413
Bacteria_Firmicutes_A_Clostridia_Lachnospirales_Lachnospiraceae_Blautia_A_Blautia_A massiliensis_MB2bin79	Structural complex_ Genetic information processing_ DNA polymerase_DNA polymerase III complex, bacteria	0,003	0,039	0,465
Bacteria_Firmicutes_A_Clostridia_Lachnospirales_Lachnospiraceae_Blautia_A_Blautia_A sp900066335_MB2bin25	Structural complex_ Genetic information processing_ DNA polymerase_DNA polymerase III complex, bacteria	0,001	0,024	0,520
Bacteria_Firmicutes_A_Clostridia_Lachnospirales_Lachnospiraceae_Ruminococcus_A_Ruminococcus_A sp000437095_MB2bin155	Structural complex_ Genetic information processing_ DNA polymerase_DNA polymerase III complex, bacteria	0,014	0,099	0,389
Bacteria_Firmicutes_A_Clostridia_Lachnospirales_Lachnospiraceae_Ruminococcus_A_Ruminococcus_A sp003011855_MB2bin82	Structural complex_ Genetic information processing_ DNA polymerase_DNA polymerase III complex, bacteria	0,011	0,081	0,404
Bacteria_Firmicutes_A_Clostridia_Oscillospirales_Oscillospiraceae_ER4_?_MB2bin17	Structural complex_ Genetic information processing_ DNA polymerase_DNA polymerase III complex, bacteria	0,007	0,062	0,422
Bacteria_Firmicutes_A_Clostridia_Oscillospirales_Oscillospiraceae_Oscillibacter_?_MB2bin147	Structural complex_ Genetic information processing_ DNA polymerase_DNA polymerase III complex, bacteria	0,016	0,100	-0,382
Bacteria_Firmicutes_A_Clostridia_Oscillospirales_Ruminococcaceae_Faecalibacterium_Faecalibacterium sp900539945_MB2bin59	Structural complex_ Genetic information processing_ DNA polymerase_DNA polymerase III complex, bacteria	0,015	0,099	0,387

Bacteria_Firmicutes_I_Bacilli_A_Erysipelotrichales_Erysipelotrichaceae_Holdemanella_Holdemanella sp002299315_MB2bin140	Structural complex_ Genetic information processing_ DNA polymerase_DNA polymerase III complex, bacteria	0,003	0,039	0,462
Bacteria_Verrucomicrobiota_Verrucomicrobiae_Verrucomicrobiales_Akkermansiaceae_Akkermansia_Akkermansia muciniphila_B_MB2bin125	Structural complex_ Genetic information processing_ DNA polymerase_DNA polymerase III complex, bacteria	0,016	0,099	-0,385

**Correlations in CO KEGG Modules**				
**MGS**	**Pathway/KEGG Module**	**p-Value**	**q-Value**	**corr-coeff**
Bacteria_Firmicutes_A_Clostridia_Lachnospirales_Lachnospiraceae_Mediterraneibacter_Mediterraneibacter faecis_MB2bin127	Structural complex_ Environmental information processing_ ABC-2 type and other transport systems_ABC-2 type transport system	0,001	0,058	0,521
Bacteria_Firmicutes_A_Clostridia_Lachnospirales_Lachnospirales_Dorea_Dorea longicatena_MB2bin81	Structural complex_ Environmental information processing_ ABC-2 type and other transport systems_ABC-2 type transport system	0,001	0,058	0,528
Bacteria_Firmicutes_A_Clostridia_Peptostreptococcales_Peptostreptococcaceae_Romboutsia_Romboutsia lituseburensis_specI_v3_Cluster6795	Structural complex_ Environmental information processing_ ABC-2 type and other transport systems_ABC-2 type transport system	0,002	0,086	0,491
Bacteria_Firmicutes_A_Clostridia_Oscillospirales_Oscillospiraceae_CAG-83_?_MB2bin100	Pathway module_ Carbohydrate and lipid metabolism_ Terpenoid backbone biosynthesis_C10-C20 isoprenoid biosynthesis, bacteria	0,005	0,049	0,442
Bacteria_Firmicutes_A_Clostridia_Oscillospirales_Oscillospiraceae_CAG-103_?_MB2bin101	Pathway module_ Carbohydrate and lipid metabolism_ Terpenoid backbone biosynthesis_C10-C20 isoprenoid biosynthesis, bacteria	0,000	0,008	0,580
Bacteria_Firmicutes_A_Clostridia_Lachnospirales_Lachnospiraceae_Eubacterium_I_Eubacterium_I ramulus_MB2bin11	Pathway module_ Carbohydrate and lipid metabolism_ Terpenoid backbone biosynthesis_C10-C20 isoprenoid biosynthesis, bacteria	0,008	0,063	-0,421
Bacteria_Firmicutes_A_Clostridia_Lachnospirales_Lachnospiraceae_Lachnospira_Lachnospira rogosae_MB2bin119	Pathway module_ Carbohydrate and lipid metabolism_ Terpenoid backbone biosynthesis_C10-C20	0,016	0,087	-0,383

	isoprenoid biosynthesis, bacteria			
Bacteria_Firmicutes_A_Clostridia_Lachnospirales_Lachnospiraceae_Anaerobutyricum_?_MB2bin124	Pathway module_ Carbohydrate and lipid metabolism_ Terpenoid backbone biosynthesis_C10-C20 isoprenoid biosynthesis, bacteria	0,000	0,008	-0,580
Bacteria_Firmicutes_A_Clostridia_Lachnospirales_Lachnospiraceae_Blautia_A_?_MB2bin128	Pathway module_ Carbohydrate and lipid metabolism_ Terpenoid backbone biosynthesis_C10-C20 isoprenoid biosynthesis, bacteria	0,001	0,021	-0,512
Bacteria_Actinobacteriota_Actinomycetia_Actinomycetales_Bifidobacteriaceae_Bifidobacterium_Bifidobacterium adolescentis_MB2bin135	Pathway module_ Carbohydrate and lipid metabolism_ Terpenoid backbone biosynthesis_C10-C20 isoprenoid biosynthesis, bacteria	0,008	0,063	-0,420
Bacteria_Firmicutes_A_Clostridia_Lachnospirales_Lachnospiraceae_Blautia_A_?_MB2bin14	Pathway module_ Carbohydrate and lipid metabolism_ Terpenoid backbone biosynthesis_C10-C20 isoprenoid biosynthesis, bacteria	0,000	0,008	-0,571
Bacteria_Firmicutes_A_Clostridia_Oscillospirales_Oscillospiraceae_CAG-170_CAG-170 sp003516765_MB2bin143	Pathway module_ Carbohydrate and lipid metabolism_ Terpenoid backbone biosynthesis_C10-C20 isoprenoid biosynthesis, bacteria	0,010	0,073	0,409
Bacteria_Firmicutes_A_Clostridia_Oscillospirales_Oscillospiraceae_Oscillibacter_?_MB2bin147	Pathway module_ Carbohydrate and lipid metabolism_ Terpenoid backbone biosynthesis_C10-C20 isoprenoid biosynthesis, bacteria	0,002	0,034	0,481
Bacteria_Firmicutes_A_Clostridia_Oscillospirales_Oscillospiraceae_CAG-103_CAG-103 sp000432375_MB2bin162	Pathway module_ Carbohydrate and lipid metabolism_ Terpenoid backbone biosynthesis_C10-C20 isoprenoid biosynthesis, bacteria	0,001	0,018	0,526
Bacteria_Firmicutes_A_Clostridia_Lachnospirales_Lachnospiraceae_Eubacterium_I_Eubacterium_I sp900546495_MB2bin174	Pathway module_ Carbohydrate and lipid metabolism_ Terpenoid backbone biosynthesis_C10-C20 isoprenoid biosynthesis, bacteria	0,002	0,032	-0,487

Bacteria_Firmicutes_A_Clostridia_Oscillospirales_Oscillospiraceae_CAG-83_?_MB2bin179	Pathway module_ Carbohydrate and lipid metabolism_ Terpenoid backbone biosynthesis_C10-C20 isoprenoid biosynthesis, bacteria	0,001	0,022	0,505
Bacteria_Bacteroidota_Bacteroidia_Bacteroidales_Tannerellaceae_Parabacteroides_Parabacteroides johnsonii_MB2bin185	Pathway module_ Carbohydrate and lipid metabolism_ Terpenoid backbone biosynthesis_C10-C20 isoprenoid biosynthesis, bacteria	0,002	0,036	0,473
Bacteria_Actinobacteriota_Coriobacteriia_Coriobacteriales_Eggerthellaceae_Adlercreutzia_Adlercreutzia celatus_A_MB2bin193	Pathway module_ Carbohydrate and lipid metabolism_ Terpenoid backbone biosynthesis_C10-C20 isoprenoid biosynthesis, bacteria	0,016	0,087	-0,384
Bacteria_Bacteroidota_Bacteroidia_Bacteroidales_Muribaculaceae_CAG-279_CAG-279 sp000437795_MB2bin226	Pathway module_ Carbohydrate and lipid metabolism_ Terpenoid backbone biosynthesis_C10-C20 isoprenoid biosynthesis, bacteria	0,011	0,074	0,404
Bacteria_Firmicutes_A_Clostridia_Lachnospirales_Lachnospiraceae_CAG-45_CAG-45 sp000438375_MB2bin229	Pathway module_ Carbohydrate and lipid metabolism_ Terpenoid backbone biosynthesis_C10-C20 isoprenoid biosynthesis, bacteria	0,006	0,056	0,433
Archaea_Methanobacteriota_Methanobacteria_Methanobacteriales_Methanobacteriaceae_Methanobrevibacter_A_Methanobrevibacter_A smithii_MB2bin267	Pathway module_ Carbohydrate and lipid metabolism_ Terpenoid backbone biosynthesis_C10-C20 isoprenoid biosynthesis, bacteria	0,014	0,087	-0,392
Bacteria_Firmicutes_A_Clostridia_Oscillospirales_Oscillospiraceae_CAG-170_CAG-170 sp000432135_MB2bin27	Pathway module_ Carbohydrate and lipid metabolism_ Terpenoid backbone biosynthesis_C10-C20 isoprenoid biosynthesis, bacteria	0,003	0,039	0,459
Bacteria_Actinobacteriota_Actinomycetia_Actinomycetales_Bifidobacteriaceae_Bifidobacterium_Bifidobacterium angulatum_MB2bin288	Pathway module_ Carbohydrate and lipid metabolism_ Terpenoid backbone biosynthesis_C10-C20 isoprenoid biosynthesis, bacteria	0,004	0,042	-0,452
Bacteria_Firmicutes_A_Clostridia_Lachnospirales_Lachnospiraceae_Anaerobutyricum_Anaerobutyricum hallii_MB2bin3	Pathway module_ Carbohydrate and lipid metabolism_ Terpenoid backbone biosynthesis_C10-C20	0,002	0,036	-0,471

	isoprenoid biosynthesis, bacteria			
Bacteria_Firmicutes_A_Clostridia_Oscillospirales_Oscillospiraceae_CAG-170_CAG-170 sp900545925_MB2bin33	Pathway module_ Carbohydrate and lipid metabolism_ Terpenoid backbone biosynthesis_C10-C20 isoprenoid biosynthesis, bacteria	0,000	0,015	0,545
Bacteria_Firmicutes_A_Clostridia_Lachnospirales_Lachnospiraceae_UBA11774_UBA11774 sp003507655_MB2bin41	Pathway module_ Carbohydrate and lipid metabolism_ Terpenoid backbone biosynthesis_C10-C20 isoprenoid biosynthesis, bacteria	0,017	0,087	0,381
Bacteria_Firmicutes_A_Clostridia_Lachnospirales_Lachnospiraceae_Anaerostipes_Anaerostipes hadrus_MB2bin47	Pathway module_ Carbohydrate and lipid metabolism_ Terpenoid backbone biosynthesis_C10-C20 isoprenoid biosynthesis, bacteria	0,001	0,018	-0,523
Bacteria_Bacteroidota_Bacteroidia_Bacteroidales_Tannerellaceae_Parabacteroides_Parabacteroides merdae_MB2bin5	Pathway module_ Carbohydrate and lipid metabolism_ Terpenoid backbone biosynthesis_C10-C20 isoprenoid biosynthesis, bacteria	0,003	0,039	0,458
Bacteria_Firmicutes_A_Clostridia_Lachnospirales_Lachnospiraceae_Mediterraneibacter_Mediterraneibacter torques_MB2bin56	Pathway module_ Carbohydrate and lipid metabolism_ Terpenoid backbone biosynthesis_C10-C20 isoprenoid biosynthesis, bacteria	0,010	0,073	-0,410
Bacteria_Firmicutes_A_Clostridia_Oscillospirales_Ruminococcaceae_Ruminococcus_D_?_MB2bin60	Pathway module_ Carbohydrate and lipid metabolism_ Terpenoid backbone biosynthesis_C10-C20 isoprenoid biosynthesis, bacteria	0,007	0,063	-0,423
Bacteria_Firmicutes_A_Clostridia_Oscillospirales_Ruminococcaceae_Faecalibacterium_Faecalibacterium prausnitzii_I_MB2bin63	Pathway module_ Carbohydrate and lipid metabolism_ Terpenoid backbone biosynthesis_C10-C20 isoprenoid biosynthesis, bacteria	0,011	0,074	0,405
Bacteria_Firmicutes_A_Clostridia_Lachnospirales_Lachnospiraceae_Blautia_A_Blautia_A sp900066355_MGS00045	Pathway module_ Carbohydrate and lipid metabolism_ Terpenoid backbone biosynthesis_C10-C20 isoprenoid biosynthesis, bacteria	0,014	0,087	-0,390

Bacteria_Bacteroidota_Bacteroidia_Bacteroidales_Rikenellaceae_Alistipes_Alistipes sp900083545_specI_v3_Cluster1016	Pathway module_ Carbohydrate and lipid metabolism_ Terpenoid backbone biosynthesis_C10-C20 isoprenoid biosynthesis, bacteria	0,003	0,038	0,465
Bacteria_Firmicutes_I_Bacilli_A_Lactobacillales_Streptococcaceae_Streptococcus_Streptococcus sp000187445_specI_v3_Cluster1349	Pathway module_ Carbohydrate and lipid metabolism_ Terpenoid backbone biosynthesis_C10-C20 isoprenoid biosynthesis, bacteria	0,016	0,087	-0,384
Bacteria_Actinobacteriota_Actinobacteria_Actinomycetales_Bifidobacteriaceae_Bifidobacterium_Bifidobacterium ruminantium_specI_v3_Cluster2702	Pathway module_ Carbohydrate and lipid metabolism_ Terpenoid backbone biosynthesis_C10-C20 isoprenoid biosynthesis, bacteria	0,015	0,087	-0,387
Bacteria_Bacteroidota_Bacteroidia_Bacteroidales_Bacteroidaceae_Prevotella_Prevotella sp900313215_specI_v3_Cluster7696	Pathway module_ Carbohydrate and lipid metabolism_ Terpenoid backbone biosynthesis_C10-C20 isoprenoid biosynthesis, bacteria	0,016	0,087	0,382
Bacteria_Firmicutes_A_Clostridia_Oscillospirales_Oscillospiraceae_CAG-83_?_MB2bin100	Pathway module_ Nucleotide and amino acid metabolism_ Aromatic amino acid metabolism_Shikimate pathway, phosphoenolpyruvate + erythrose-4P => chorismate	0,000	0,003	0,627
Bacteria_Bacteroidota_Bacteroidia_Bacteroidales_Bacteroidaceae_Paraprevotella_Paraprevotella clara_MB2bin115	Pathway module_ Nucleotide and amino acid metabolism_ Aromatic amino acid metabolism_Shikimate pathway, phosphoenolpyruvate + erythrose-4P => chorismate	0,003	0,057	0,469
Bacteria_Bacteroidota_Bacteroidia_Bacteroidales_Bacteroidaceae_Bacteroides_Bacteroides thetaiotaomicron_MB2bin137	Pathway module_ Nucleotide and amino acid metabolism_ Aromatic amino acid metabolism_Shikimate pathway, phosphoenolpyruvate + erythrose-4P => chorismate	0,004	0,065	-0,453
Bacteria_Firmicutes_A_Clostridia_Oscillospirales_Oscillospiraceae_ER4_?_MB2bin17	Pathway module_ Nucleotide and amino acid metabolism_ Aromatic amino acid metabolism_Shikimate pathway, phosphoenolpyruvate + erythrose-4P => chorismate	0,002	0,056	0,491
Bacteria_Actinobacteriota_Actinomycetia_Actinomycetales_Bifidobacteriaceae_Bifidobacterium_Bif	Pathway module_ Nucleotide and amino acid metabolism_ Aromatic amino acid metabolism_Shikimate	0,003	0,057	-0,465

idobacterium bifidum_MB2bin203	pathway, phosphoenolpyruvate + erythrose-4P => chorismate			
Bacteria_Actinobacteriota_Coriobacteriia_Coriobacteriales_Eggerthellaceae_CAG-1427_CAG-1427 sp000436075_MB2bin206	Pathway module_ Nucleotide and amino acid metabolism_ Aromatic amino acid metabolism_Shikimate pathway, phosphoenolpyruvate + erythrose-4P => chorismate	0,002	0,056	0,488
Bacteria_Actinobacteriota_Coriobacteriia_Coriobacteriales_Eggerthellaceae_CAG-1427_?_MB2bin289	Pathway module_ Nucleotide and amino acid metabolism_ Aromatic amino acid metabolism_Shikimate pathway, phosphoenolpyruvate + erythrose-4P => chorismate	0,003	0,057	0,463
Bacteria_Firmicutes_A_Clostridia_Lachnospirales_Lachnospiraceae_Fusicatenibacter_Fusicatenibacter saccharivorans_MB2bin29	Pathway module_ Nucleotide and amino acid metabolism_ Aromatic amino acid metabolism_Shikimate pathway, phosphoenolpyruvate + erythrose-4P => chorismate	0,000	0,018	-0,560
Bacteria_Firmicutes_A_Clostridia_Lachnospirales_Lachnospiraceae_Mediterraneibacter_Mediterraneibacter torques_MB2bin56	Pathway module_ Nucleotide and amino acid metabolism_ Aromatic amino acid metabolism_Shikimate pathway, phosphoenolpyruvate + erythrose-4P => chorismate	0,002	0,057	-0,476
Bacteria_Firmicutes_A_Clostridia_Lachnospirales_Lachnospiraceae_Blautia_A_?_MB2bin9	Pathway module_ Nucleotide and amino acid metabolism_ Aromatic amino acid metabolism_Shikimate pathway, phosphoenolpyruvate + erythrose-4P => chorismate	0,001	0,056	-0,507
Bacteria_Actinobacteriota_Actinomycetia_Actinomycetales_Bifidobacteriaceae_Bifidobacterium_Bifidobacterium longum_MB2bin141	Pathway module_ Carbohydrate and lipid metabolism_ Lipid metabolism_Triacylglycerol biosynthesis	0,000	0,001	-0,640
Bacteria_Firmicutes_A_Clostridia_Lachnospirales_Lachnospiraceae_Coprococcus_Coprococcus eutactus_MB2bin153	Pathway module_ Carbohydrate and lipid metabolism_ Lipid metabolism_Triacylglycerol biosynthesis	0,001	0,055	0,499
Bacteria_Firmicutes_A_Clostridia_Lachnospirales_Lachnospiraceae_Mediterraneibacter_Mediterraneibacter lactaris_MB2bin26	Pathway module_ Carbohydrate and lipid metabolism_ Lipid metabolism_Triacylglycerol biosynthesis	0,003	0,080	0,466
Bacteria_Firmicutes_A_Clostridia_Lachnospirales_Lachnospiraceae_Coprococcus_Coprococcus eutactus_A_MB2bin7	Pathway module_ Carbohydrate and lipid metabolism_ Lipid metabolism_Triacylglycerol biosynthesis	0,001	0,055	0,497

Bacteria_Actinobacteriota_Actinobacteria_Actinomycetales_Bifidobacteriaceae_Bifidobacterium_Bifidobacterium breve_specI_v3_Cluster1098	Pathway module_ Carbohydrate and lipid metabolism_ Lipid metabolism_Triacylglycerol biosynthesis	0,000	0,001	-0,641
Bacteria_Bacteroidota_Bacteroidia_Bacteroidales_Bacteroidaceae_Bacteroides_Bacteroides caccae_specI_v3_Cluster3473	Pathway module_ Carbohydrate and lipid metabolism_ Lipid metabolism_Triacylglycerol biosynthesis	0,002	0,069	0,480
Bacteria_Bacteroidota_Bacteroidia_Bacteroidales_Bacteroidaceae_Bacteroides_Bacteroides uniformis_MB2bin1	Pathway module_ Nucleotide and amino acid metabolism_ Histidine metabolism_Histidine biosynthesis, PRPP => histidine	0,008	0,050	-0,418
Bacteria_Bacteroidota_Bacteroidia_Bacteroidales_Rikenellaceae_Alistipes_Alistipes shahii_MB2bin10	Pathway module_ Nucleotide and amino acid metabolism_ Histidine metabolism_Histidine biosynthesis, PRPP => histidine	0,000	0,012	-0,585
Bacteria_Firmicutes_A_Clostridia_Oscillospirales_Oscillospiraceae_CAG-103_?_MB2bin101	Pathway module_ Nucleotide and amino acid metabolism_ Histidine metabolism_Histidine biosynthesis, PRPP => histidine	0,006	0,043	-0,431
Bacteria_Bacteroidota_Bacteroidia_Bacteroidales_Bacteroidaceae_Prevotella_Prevotella copri_A_MB2bin109	Pathway module_ Nucleotide and amino acid metabolism_ Histidine metabolism_Histidine biosynthesis, PRPP => histidine	0,003	0,036	0,460
Bacteria_Bacteroidota_Bacteroidia_Bacteroidales_Bacteroidaceae_Bacteroides_Bacteroides ovatus_MB2bin110	Pathway module_ Nucleotide and amino acid metabolism_ Histidine metabolism_Histidine biosynthesis, PRPP => histidine	0,006	0,043	-0,432
Bacteria_Firmicutes_A_Clostridia_Lachnospirales_Lachnospiraceae_Blautia_A_Blautia_A sp900066145_MB2bin111	Pathway module_ Nucleotide and amino acid metabolism_ Histidine metabolism_Histidine biosynthesis, PRPP => histidine	0,006	0,043	0,429
Bacteria_Firmicutes_A_Clostridia_Lachnospirales_Lachnospirales_Dorea_Dorea formicigenerans_MB2bin120	Pathway module_ Nucleotide and amino acid metabolism_ Histidine metabolism_Histidine biosynthesis, PRPP => histidine	0,001	0,032	0,519
Bacteria_Firmicutes_A_Clostridia_Lachnospirales_Lachnospiraceae_Anaerobutyricum_?_MB2bin124	Pathway module_ Nucleotide and amino acid metabolism_ Histidine metabolism_Histidine biosynthesis, PRPP => histidine	0,002	0,036	0,474
Bacteria_Firmicutes_A_Clostridia_Lachnospirales_Lachnospiraceae_Mediterraneibacter_Mediterraneibacter faecis_MB2bin127	Pathway module_ Nucleotide and amino acid metabolism_ Histidine metabolism_Histidine biosynthesis, PRPP => histidine	0,012	0,057	0,397
Bacteria_Firmicutes_A_Clostridia_Lachnospirales_Lachnospiraceae_Agathobacter_Agathobacter faecis_MB2bin13	Pathway module_ Nucleotide and amino acid metabolism_ Histidine metabolism_Histidine biosynthesis, PRPP => histidine	0,005	0,043	0,439
Bacteria_Firmicutes_A_Clostridia_Lachnospirales_Lachnospiraceae_Blautia_A_?_MB2bin14	Pathway module_ Nucleotide and amino acid metabolism_	0,003	0,036	0,469

	Histidine metabolism_Histidine biosynthesis, PRPP => histidine			
Bacteria_Firmicutes_A_Clostridia_Lachnospirales_Lachnospiraceae_Coprococcus_Coprococcus eutactus_MB2bin153	Pathway module_ Nucleotide and amino acid metabolism_ Histidine metabolism_Histidine biosynthesis, PRPP => histidine	0,004	0,036	0,455
Bacteria_Firmicutes_A_Clostridia_Lachnospirales_Lachnospiraceae_Ruminococcus_A_Ruminococcus_A sp000437095_MB2bin155	Pathway module_ Nucleotide and amino acid metabolism_ Histidine metabolism_Histidine biosynthesis, PRPP => histidine	0,011	0,056	0,401
Bacteria_Firmicutes_A_Clostridia_Oscillospirales_Acutalibacteraceae_CAG-180_CAG-180 sp000432435_MB2bin173	Pathway module_ Nucleotide and amino acid metabolism_ Histidine metabolism_Histidine biosynthesis, PRPP => histidine	0,003	0,036	0,457
Bacteria_Firmicutes_A_Clostridia_Lachnospirales_Lachnospiraceae_Blautia_A_?_MB2bin175	Pathway module_ Nucleotide and amino acid metabolism_ Histidine metabolism_Histidine biosynthesis, PRPP => histidine	0,009	0,053	0,413
Bacteria_Bacteroidota_Bacteroidia_Bacteroidales_Tannerellaceae_Parabacteroides_Parabacteroides johnsonii_MB2bin185	Pathway module_ Nucleotide and amino acid metabolism_ Histidine metabolism_Histidine biosynthesis, PRPP => histidine	0,004	0,039	-0,447
Bacteria_Firmicutes_A_Clostridia_Lachnospirales_Lachnospiraceae_Bariatricus_Bariatricus comes_MB2bin19	Pathway module_ Nucleotide and amino acid metabolism_ Histidine metabolism_Histidine biosynthesis, PRPP => histidine	0,010	0,055	0,409
Bacteria_Actinobacteriota_Coriobacteriia_Coriobacteriales_Eggerthellaceae_Adlercreutzia_Adlercreutzia celatus_A_MB2bin193	Pathway module_ Nucleotide and amino acid metabolism_ Histidine metabolism_Histidine biosynthesis, PRPP => histidine	0,020	0,091	0,371
Bacteria_Firmicutes_A_Clostridia_Lachnospirales_Lachnospiraceae_Blautia_A_Blautia_A sp900066165_MB2bin2	Pathway module_ Nucleotide and amino acid metabolism_ Histidine metabolism_Histidine biosynthesis, PRPP => histidine	0,010	0,055	0,407
Bacteria_Actinobacteriota_Coriobacteriia_Coriobacteriales_Coriobacteriaceae_Collinsella_Collinsella sp000763055_MB2bin202	Pathway module_ Nucleotide and amino acid metabolism_ Histidine metabolism_Histidine biosynthesis, PRPP => histidine	0,003	0,036	0,462
Bacteria_Firmicutes_A_Clostridia_Oscillospirales_Ruminococcaceae_Ruminiclostridium_E_Ruminiclostridium_E sp003512525_MB2bin228	Pathway module_ Nucleotide and amino acid metabolism_ Histidine metabolism_Histidine biosynthesis, PRPP => histidine	0,003	0,036	-0,470
Bacteria_Firmicutes_A_Clostridia_Lachnospirales_Lachnospiraceae_CAG-45_CAG-45 sp000438375_MB2bin229	Pathway module_ Nucleotide and amino acid metabolism_ Histidine metabolism_Histidine biosynthesis, PRPP => histidine	0,006	0,043	-0,432
Bacteria_Firmicutes_A_Clostridia_Lachnospirales_Lachnospiraceae_Fusicatenibacter_Fusicatenibacter saccharivorans_MB2bin29	Pathway module_ Nucleotide and amino acid metabolism_ Histidine metabolism_Histidine biosynthesis, PRPP => histidine	0,004	0,036	0,456

Bacteria_Firmicutes_A_Clostridia_Lachnospirales_Lachnospiraceae_Anaerobutyricum_Anaerobutyricum hallii_MB2bin3	Pathway module_ Nucleotide and amino acid metabolism_ Histidine metabolism_Histidine biosynthesis, PRPP => histidine	0,003	0,036	0,458
Bacteria_Firmicutes_A_Clostridia_Oscillospirales_Ruminococcaceae_Ruminococcus_C_Ruminococcus_C sp000433635_MB2bin321	Pathway module_ Nucleotide and amino acid metabolism_ Histidine metabolism_Histidine biosynthesis, PRPP => histidine	0,001	0,032	0,509
Bacteria_Firmicutes_A_Clostridia_Oscillospirales_Ruminococcaceae_Gemmiger_Gemmiger sp900539695_MB2bin39	Pathway module_ Nucleotide and amino acid metabolism_ Histidine metabolism_Histidine biosynthesis, PRPP => histidine	0,003	0,036	0,463
Bacteria_Bacteroidota_Bacteroidia_Bacteroidales_Tannerellaceae_Parabacteroides_Parabacteroides merdae_MB2bin5	Pathway module_ Nucleotide and amino acid metabolism_ Histidine metabolism_Histidine biosynthesis, PRPP => histidine	0,007	0,043	-0,426
Bacteria_Firmicutes_A_Clostridia_Oscillospirales_Ruminococcaceae_Ruminiclostridium_E_Ruminiclostridium_E siraeum_MB2bin52	Pathway module_ Nucleotide and amino acid metabolism_ Histidine metabolism_Histidine biosynthesis, PRPP => histidine	0,012	0,057	-0,397
Bacteria_Firmicutes_A_Clostridia_Lachnospirales_Lachnospiraceae_Mediterraneibacter_Mediterraneibacter torques_MB2bin56	Pathway module_ Nucleotide and amino acid metabolism_ Histidine metabolism_Histidine biosynthesis, PRPP => histidine	0,011	0,055	0,404
Bacteria_Firmicutes_A_Clostridia_Lachnospirales_Lachnospiraceae_Coprococcus_Coprococcus eutactus_A_MB2bin7	Pathway module_ Nucleotide and amino acid metabolism_ Histidine metabolism_Histidine biosynthesis, PRPP => histidine	0,004	0,039	0,450
Bacteria_Firmicutes_A_Clostridia_Oscillospirales_Butyricicoccaceae_Agathobaculum_Agathobaculum butyriciproducens_MB2bin73	Pathway module_ Nucleotide and amino acid metabolism_ Histidine metabolism_Histidine biosynthesis, PRPP => histidine	0,006	0,043	0,436
Bacteria_Actinobacteriota_Coriobacteriia_Coriobacteriales_Coriobacteriaceae_Collinsella_?_MB2bin75	Pathway module_ Nucleotide and amino acid metabolism_ Histidine metabolism_Histidine biosynthesis, PRPP => histidine	0,005	0,041	0,443
Bacteria_Firmicutes_A_Clostridia_Oscillospirales_Ruminococcaceae_Faecalibacterium_Faecalibacterium prausnitzii_A_MB2bin78	Pathway module_ Nucleotide and amino acid metabolism_ Histidine metabolism_Histidine biosynthesis, PRPP => histidine	0,011	0,055	0,404
Bacteria_Firmicutes_A_Clostridia_Lachnospirales_Lachnospiraceae_Ruminococcus_A_Ruminococcus_A sp003011855_MB2bin82	Pathway module_ Nucleotide and amino acid metabolism_ Histidine metabolism_Histidine biosynthesis, PRPP => histidine	0,001	0,032	0,513
Bacteria_Firmicutes_A_Clostridia_Oscillospirales_Acutalibacteraceae_Ruminococcus_H_Ruminococcus_H sp003531055_MB2bin93	Pathway module_ Nucleotide and amino acid metabolism_ Histidine metabolism_Histidine biosynthesis, PRPP => histidine	0,001	0,036	0,492
Bacteria_Firmicutes_A_Clostridia_Lachnospirales_Lachnospiraceae_Blautia_A_Blautia_A sp900066355_MGS00045	Pathway module_ Nucleotide and amino acid metabolism_ Histidine metabolism_Histidine biosynthesis, PRPP => histidine	0,001	0,034	0,500

Bacteria_Bacteroidota_Bacteroidia_Bacteroidales_Rikenellaceae_Alistipes_Alistipes sp900083545_specI_v3_Cluster1016	Pathway module_ Nucleotide and amino acid metabolism_ Histidine metabolism_Histidine biosynthesis, PRPP => histidine	0,000	0,012	-0,573
Bacteria_Bacteroidota_Bacteroidia_Bacteroidales_Bacteroidaceae_Bacteroides_B_Bacteroides_B sartorii_specI_v3_Cluster2366	Pathway module_ Nucleotide and amino acid metabolism_ Histidine metabolism_Histidine biosynthesis, PRPP => histidine	0,010	0,055	-0,409
Bacteria_Firmicutes_A_Clostridia_Oscillospirales_Oscillospiraceae_CAG-83_?_MB2bin100	Structural complex_ Environmental information processing_ Mineral and organic ion transport system_Iron(III) transport system	0,000	0,064	0,541
Bacteria_Firmicutes_A_Clostridia_Oscillospirales_Oscillospiraceae_CAG-103_?_MB2bin101	Structural complex_ Environmental information processing_ Saccharide, polyol, and lipid transport system_Multiple sugar transport system	0,001	0,031	-0,514
Bacteria_Actinobacteriota_Actinomycetia_Actinomycetales_Bifidobacteriaceae_Bifidobacterium_Bifidobacterium longum_MB2bin141	Structural complex_ Environmental information processing_ Saccharide, polyol, and lipid transport system_Multiple sugar transport system	0,002	0,059	0,476
Bacteria_Firmicutes_A_Clostridia_Oscillospirales_Oscillospiraceae_Oscillibacter_?_MB2bin147	Structural complex_ Environmental information processing_ Saccharide, polyol, and lipid transport system_Multiple sugar transport system	0,001	0,031	-0,510
Bacteria_Firmicutes_A_Clostridia_Oscillospirales_Oscillospiraceae_CAG-83_?_MB2bin179	Structural complex_ Environmental information processing_ Saccharide, polyol, and lipid transport system_Multiple sugar transport system	0,000	0,027	-0,533
Bacteria_Bacteroidota_Bacteroidia_Bacteroidales_Rikenellaceae_Alistipes_Alistipes obesi_MB2bin28	Structural complex_ Environmental information processing_ Saccharide, polyol, and lipid transport system_Multiple sugar transport system	0,000	0,003	-0,628
Bacteria_Bacteroidota_Bacteroidia_Bacteroidales_Rikenellaceae_Alistipes_Alistipes finegoldii_MB2bin46	Structural complex_ Environmental information processing_ Saccharide, polyol, and lipid transport system_Multiple sugar transport system	0,003	0,059	-0,466

Bacteria_Firmicutes_A_Clostridia_Lachnospirales_Lachnospiraceae_Anaerostipes_Anaerostipes hadrus_MB2bin47	Structural complex_ Environmental information processing_ Saccharide, polyol, and lipid transport system_Multiple sugar transport system	0,000	0,015	0,567
Bacteria_Firmicutes_A_Clostridia_Lachnospirales_Lachnospiraceae_Blautia_A_Blautia_A sp900548245_MB2bin70	Structural complex_ Environmental information processing_ Saccharide, polyol, and lipid transport system_Multiple sugar transport system	0,005	0,092	0,437
Bacteria_Firmicutes_C_Negativicutes_Veillonellales_Dialisteraceae_Dialister_Dialister succinatiphilus_specI_v3_Cluster11863	Structural complex_ Environmental information processing_ Saccharide, polyol, and lipid transport system_Multiple sugar transport system	0,005	0,092	0,438
Bacteria_Firmicutes_A_Clostridia_Lachnospirales_Lachnospiraceae_Anaerostipes_Anaerostipes hadrus_A_specI_v3_Cluster856	Structural complex_ Environmental information processing_ Saccharide, polyol, and lipid transport system_Multiple sugar transport system	0,002	0,059	0,472

**Correlations in PD and CO GB Modules**				
**MGS**	**Pathway/GBM Module**	**p-Value**	**q-Value**	**corr-coeff**
Archaea_Methanobacteriota_Methanobacteria_Methanobacteriales_Methanobacteriaceae_Methanobrevibacter_A_Methanobrevibacter_A smithii_MB2bin267	GABA synthesis_GABA synthesis III_GABA synthesis III	0,001	0,018	0,501
Bacteria_Actinobacteriota_Actinobacteria_Actinomycetales_Bifidobacteriaceae_Bifidobacterium_Bifidobacterium breve_specI_v3_Cluster1098	ClpB_ClpB (ATP-dependent chaperone protein)_ClpB (ATP-dependent chaperone protein)	0,001	0,076	0,511
Bacteria_Actinobacteriota_Actinobacteria_Actinomycetales_Bifidobacteriaceae_Bifidobacterium_Bifidobacterium ruminantium_specI_v3_Cluster2702	ClpB_ClpB (ATP-dependent chaperone protein)_ClpB (ATP-dependent chaperone protein)	0,004	0,099	0,446
Bacteria_Actinobacteriota_Actinomycetia_Actinomycetales_Bifidobacteriaceae_Bifidobacterium_Bifidobacterium adolescentis_MB2bin135	ClpB_ClpB (ATP-dependent chaperone protein)_ClpB (ATP-dependent chaperone protein)	0,003	0,099	0,457

Bacteria_Actinobacteriota_Actinomycetia_Actinomycetales_Bifidobacteriaceae_Bifidobacterium_Bifidobacterium longum_MB2bin141	ClpB_ClpB (ATP-dependent chaperone protein)_ClpB (ATP-dependent chaperone protein)	0,001	0,076	0,526
Bacteria_Actinobacteriota_Coriobacteriia_Coriobacteriales_Eggerthellaceae_CAG-1427_?_MB2bin289	GABA synthesis_GABA synthesis III_GABA synthesis III	0,010	0,072	-0,409
Bacteria_Bacteroidota_Bacteroidia_Bacteroidales_Bacteroidaceae_Bacteroides_Bacteroides fragilis_MB2bin178	ClpB_ClpB (ATP-dependent chaperone protein)_ClpB (ATP-dependent chaperone protein)	0,004	0,099	-0,447
Bacteria_Bacteroidota_Bacteroidia_Bacteroidales_Bacteroidaceae_Phocaeicola_Phocaeicola plebeius_A_MB2bin65	GABA synthesis_GABA synthesis III_GABA synthesis III	0,003	0,042	-0,461
Bacteria_Bacteroidota_Bacteroidia_Bacteroidales_Bacteroidaceae_Prevotella_Prevotella copri_A_MB2bin109	GABA synthesis_GABA synthesis III_GABA synthesis III	0,000	0,013	-0,548
Bacteria_Bacteroidota_Bacteroidia_Bacteroidales_Barnesiellaceae_Barnesiella_Barnesiella intestinihominis_MB2bin112	ClpB_ClpB (ATP-dependent chaperone protein)_ClpB (ATP-dependent chaperone protein)	0,005	0,099	-0,444
Bacteria_Bacteroidota_Bacteroidia_Bacteroidales_Rikenellaceae_Alistipes_A_Alistipes_A ihumii_MB2bin204	GABA synthesis_GABA synthesis III_GABA synthesis III	0,000	0,015	0,536
Bacteria_Bacteroidota_Bacteroidia_Bacteroidales_Rikenellaceae_Alistipes_Alistipes finegoldii_MB2bin46	ClpB_ClpB (ATP-dependent chaperone protein)_ClpB (ATP-dependent chaperone protein)	0,004	0,099	-0,448
Bacteria_Bacteroidota_Bacteroidia_Bacteroidales_Rikenellaceae_Alistipes_Alistipes obesi_MB2bin28	GABA synthesis_GABA synthesis III_GABA synthesis III	0,009	0,072	0,411
Bacteria_Bacteroidota_Bacteroidia_Bacteroidales_Rikenellaceae_Alistipes_Alistipes onderdonkii_MB2bin22	GABA synthesis_GABA synthesis III_GABA synthesis III	0,000	0,013	0,549
Bacteria_Firmicutes_A_Clostridia_A_Christensenellales_CAG-74_SFFH01_SFFH01 sp900542445_MB2bin113	GABA synthesis_GABA synthesis III_GABA synthesis III	0,001	0,017	-0,515
Bacteria_Firmicutes_A_Clostridia_A_Christensenellales_QAND01_UMGS1975_UMGS1975 sp900546685_MB2bin272	ClpB_ClpB (ATP-dependent chaperone protein)_ClpB (ATP-dependent chaperone protein)	0,002	0,092	0,488
Bacteria_Firmicutes_A_Clostridia_Lachnospirales_Lachnospiraceae_Blautia_A_Blautia_A sp900548245_MB2bin70	GABA synthesis_GABA synthesis III_GABA synthesis III	0,008	0,065	-0,421

Bacteria_Firmicutes_A_Clostridia_Lachnospirales_Lachnospiraceae_KLE1615_KLE1615 sp900066985_MB2bin161	GABA synthesis_GABA synthesis III_GABA synthesis III	0,006	0,055	-0,434
Bacteria_Firmicutes_A_Clostridia_Lachnospirales_Lachnospiraceae_Mediterraneibacter_Mediterraneibacter lactaris_MB2bin26	GABA synthesis_GABA synthesis III_GABA synthesis III	0,004	0,044	-0,455
Bacteria_Firmicutes_A_Clostridia_Lachnospirales_Lachnospirales_Dorea_Dorea formicigenerans_MB2bin120	GABA synthesis_GABA synthesis III_GABA synthesis III	0,014	0,096	-0,392
Bacteria_Firmicutes_A_Clostridia_Lachnospirales_Lachnospirales_Dorea_Dorea longicatena_MB2bin81	GABA synthesis_GABA synthesis III_GABA synthesis III	0,000	0,010	-0,578
Bacteria_Firmicutes_A_Clostridia_Oscillospirales_Butyricicoccaceae_Agathobaculum_Agathobaculum sp003481705_MB2bin186	GABA synthesis_GABA synthesis III_GABA synthesis III	0,005	0,047	-0,444
Bacteria_Firmicutes_A_Clostridia_Oscillospirales_Oscillospiraceae_CAG-83_CAG-83 sp900545585_MB2bin50	GABA synthesis_GABA synthesis III_GABA synthesis III	0,009	0,072	-0,413
Bacteria_Firmicutes_A_Clostridia_Oscillospirales_Ruminococcaceae_Faecalibacterium_Faecalibacterium prausnitzii_C_MB2bin90	GABA synthesis_GABA synthesis III_GABA synthesis III	0,002	0,027	-0,482
Bacteria_Firmicutes_A_Clostridia_Oscillospirales_Ruminococcaceae_Faecalibacterium_Faecalibacterium prausnitzii_D_MB2bin102	GABA synthesis_GABA synthesis III_GABA synthesis III	0,001	0,017	-0,511
Bacteria_Firmicutes_A_Clostridia_Oscillospirales_Ruminococcaceae_Faecalibacterium_Faecalibacterium prausnitzii_H_MB2bin261	GABA synthesis_GABA synthesis III_GABA synthesis III	0,005	0,047	-0,445
Bacteria_Firmicutes_A_Clostridia_Oscillospirales_Ruminococcaceae_Faecalibacterium_Faecalibacterium sp900539945_MB2bin59	GABA synthesis_GABA synthesis III_GABA synthesis III	0,005	0,047	-0,445
Bacteria_Firmicutes_A_Clostridia_Oscillospirales_Ruminococcaceae_Gemmiger_Gemmiger sp900539695_MB2bin39	GABA synthesis_GABA synthesis III_GABA synthesis III	0,001	0,017	-0,513
Bacteria_Firmicutes_C_Negativicutes_Acidaminococcales_Acidaminococcaceae_Phascolarctobacterium_A_?_MB2bin381	GABA synthesis_GABA synthesis III_GABA synthesis III	0,007	0,063	-0,425
Bacteria_Firmicutes_C_Negativicutes_Acidaminococcales_Acidaminococcaceae_Phascolarctobacteri	GABA synthesis_GABA synthesis III_GABA synthesis III	0,014	0,096	0,391

um_Phascolarctobacterium faecium_MB2bin404				
Bacteria_Firmicutes_C_Negativicutes_Veillonellales_Dialisteraceae_Dialister_Dialister invisus_specI_v3_Cluster3691	GABA synthesis_GABA synthesis III_GABA synthesis III	0,000	0,010	0,584
Bacteria_Verrucomicrobiota_Verrucomicrobiae_Verrucomicrobiales_Akkermansiaceae_Akkermansia_Akkermansia muciniphila_B_MB2bin125	GABA synthesis_GABA synthesis III_GABA synthesis III	0,001	0,018	0,502
Bacteria_Verrucomicrobiota_Verrucomicrobiae_Verrucomicrobiales_Akkermansiaceae_Akkermansia_Akkermansia muciniphila_MB2bin64	GABA synthesis_GABA synthesis III_GABA synthesis III	0,001	0,016	0,527

**Correlations in PD GBM Modules**				
**MGS**	**Pathway/GBM Module**	**p-Value**	**q-Value**	**corr-coeff**
Bacteria_Actinobacteriota_Actinobacteria_Actinomycetales_Bifidobacteriaceae_Bifidobacterium_Bifidobacterium ruminantium_specI_v3_Cluster2702	Inositol degradation_Inositol degradation_Inositol degradation	0,019	0,087	-0,375
Bacteria_Actinobacteriota_Actinomycetia_Actinomycetales_Bifidobacteriaceae_Bifidobacterium_Bifidobacterium adolescentis_MB2bin135	Inositol degradation_Inositol degradation_Inositol degradation	0,016	0,077	-0,385
Bacteria_Actinobacteriota_Actinomycetia_Actinomycetales_Bifidobacteriaceae_Bifidobacterium_Bifidobacterium angulatum_MB2bin288	Inositol degradation_Inositol degradation_Inositol degradation	0,015	0,076	-0,387
Bacteria_Actinobacteriota_Actinomycetia_Actinomycetales_Bifidobacteriaceae_Bifidobacterium_Bifidobacterium bifidum_MB2bin203	Acetate synthesis_Acetate synthesis III_Acetate synthesis III	0,001	0,071	-0,498
Bacteria_Actinobacteriota_Actinomycetia_Actinomycetales_Bifidobacteriaceae_Bifidobacterium_Bifidobacterium bifidum_MB2bin203	Acetate synthesis_Acetate synthesis II_Acetate synthesis II	0,001	0,058	-0,495
Bacteria_Actinobacteriota_Actinomycetia_Actinomycetales_Bifidobacteriaceae_Bifidobacterium_Bif	Glutamate synthesis_Glutamate synthesis I_Glutamate synthesis I	0,000	0,026	0,570

idobacterium bifidum_MB2bin203				
Bacteria_Actinobacteriota_Coriobacteriia_Coriobacteriales_Coriobacteriaceae_Collinsella_?_MB2bin75	Inositol degradation_Inositol degradation_Inositol degradation	0,008	0,056	-0,416
Bacteria_Actinobacteriota_Coriobacteriia_Coriobacteriales_Coriobacteriaceae_Collinsella_Collinsella aerofaciens_F_specI_v3_Cluster3625	Quinolinic acid degradation_Quinolinic acid degradation_Quinolinic acid degradation	0,000	0,002	0,591
Bacteria_Actinobacteriota_Coriobacteriia_Coriobacteriales_Coriobacteriaceae_Collinsella_Collinsella sp000763055_MB2bin202	Inositol degradation_Inositol degradation_Inositol degradation	0,003	0,031	-0,462
Bacteria_Actinobacteriota_Coriobacteriia_Coriobacteriales_Eggerthellaceae_Adlercreutzia_Adlercreutzia celatus_A_MB2bin193	Quinolinic acid degradation_Quinolinic acid degradation_Quinolinic acid degradation	0,000	0,000	-0,651
Bacteria_Actinobacteriota_Coriobacteriia_Coriobacteriales_Eggerthellaceae_CAG-1427_CAG-1427 sp000435675_MB2bin169	Inositol degradation_Inositol degradation_Inositol degradation	0,010	0,061	0,406
Bacteria_Bacteroidota_Bacteroidia_Bacteroidales_Bacteroidaceae_Bacteroides_B_Bacteroides_B sartorii_specI_v3_Cluster2366	Inositol degradation_Inositol degradation_Inositol degradation	0,002	0,025	0,477
Bacteria_Bacteroidota_Bacteroidia_Bacteroidales_Bacteroidaceae_Bacteroides_Bacteroides eggerthii_MB2bin146	Quinolinic acid degradation_Quinolinic acid degradation_Quinolinic acid degradation	0,000	0,010	-0,538
Bacteria_Bacteroidota_Bacteroidia_Bacteroidales_Bacteroidaceae_Bacteroides_Bacteroides eggerthii_MB2bin146	Inositol degradation_Inositol degradation_Inositol degradation	0,014	0,076	0,389
Bacteria_Bacteroidota_Bacteroidia_Bacteroidales_Bacteroidaceae_Bacteroides_Bacteroides fragilis_MB2bin178	Quinolinic acid degradation_Quinolinic acid degradation_Quinolinic acid degradation	0,001	0,014	-0,517
Bacteria_Bacteroidota_Bacteroidia_Bacteroidales_Bacteroidaceae_Bacteroides_Bacteroides fragilis_MB2bin178	Inositol degradation_Inositol degradation_Inositol degradation	0,001	0,019	0,500
Bacteria_Bacteroidota_Bacteroidia_Bacteroidales_Bacteroidaceae_Bacteroides_Bacteroides ovatus_MB2bin110	Quinolinic acid degradation_Quinolinic acid degradation_Quinolinic acid degradation	0,009	0,064	-0,411
Bacteria_Bacteroidota_Bacteroidia_Bacteroidales_Bacteroidaceae_Bacteroides_Bacteroides ovatus_MB2bin110	Inositol degradation_Inositol degradation_Inositol degradation	0,001	0,019	0,511

Bacteria_Bacteroidota_Bacteroidia_Bacteroidales_Bacteroidaceae_Bacteroides_Bacteroides stercoris_MB2bin42	Quinolinic acid degradation_Quinolinic acid degradation_Quinolinic acid degradation	0,000	0,010	-0,535
Bacteria_Bacteroidota_Bacteroidia_Bacteroidales_Bacteroidaceae_Bacteroides_Bacteroides stercoris_MB2bin42	Inositol degradation_Inositol degradation_Inositol degradation	0,001	0,019	0,506
Bacteria_Bacteroidota_Bacteroidia_Bacteroidales_Bacteroidaceae_Bacteroides_Bacteroides thetaiotaomicron_MB2bin137	Quinolinic acid degradation_Quinolinic acid degradation_Quinolinic acid degradation	0,006	0,051	-0,431
Bacteria_Bacteroidota_Bacteroidia_Bacteroidales_Bacteroidaceae_Bacteroides_Bacteroides thetaiotaomicron_MB2bin137	Inositol degradation_Inositol degradation_Inositol degradation	0,003	0,031	0,465
Bacteria_Bacteroidota_Bacteroidia_Bacteroidales_Bacteroidaceae_Paraprevotella_Paraprevotella clara_MB2bin115	Acetate synthesis_Acetate synthesis II_Acetate synthesis II	0,000	0,002	0,643
Bacteria_Bacteroidota_Bacteroidia_Bacteroidales_Bacteroidaceae_Paraprevotella_Paraprevotella clara_MB2bin115	Acetate synthesis_Acetate synthesis III_Acetate synthesis III	0,000	0,001	0,655
Bacteria_Bacteroidota_Bacteroidia_Bacteroidales_Bacteroidaceae_Phocaeicola_Phocaeicola plebeius_A_MB2bin65	Inositol degradation_Inositol degradation_Inositol degradation	0,003	0,032	-0,458
Bacteria_Bacteroidota_Bacteroidia_Bacteroidales_Bacteroidaceae_Phocaeicola_Phocaeicola plebeius_A_MB2bin65	Quinolinic acid degradation_Quinolinic acid degradation_Quinolinic acid degradation	0,015	0,082	0,387
Bacteria_Bacteroidota_Bacteroidia_Bacteroidales_Bacteroidaceae_Phocaeicola_Phocaeicola vulgatus_MB2bin4	Quinolinic acid degradation_Quinolinic acid degradation_Quinolinic acid degradation	0,015	0,082	-0,386
Bacteria_Bacteroidota_Bacteroidia_Bacteroidales_Bacteroidaceae_Phocaeicola_Phocaeicola vulgatus_MB2bin4	Inositol degradation_Inositol degradation_Inositol degradation	0,001	0,019	0,500
Bacteria_Bacteroidota_Bacteroidia_Bacteroidales_Bacteroidaceae_Prevotella_?_MB2bin51	Inositol degradation_Inositol degradation_Inositol degradation	0,009	0,058	-0,412
Bacteria_Bacteroidota_Bacteroidia_Bacteroidales_Bacteroidaceae_Prevotella_?_MB2bin51	Quinolinic acid degradation_Quinolinic acid degradation_Quinolinic acid degradation	0,002	0,023	0,490
Bacteria_Bacteroidota_Bacteroidia_Bacteroidales_Bacteroidaceae_Prevotella_Prevotella copri_A_MB2bin109	Inositol degradation_Inositol degradation_Inositol degradation	0,000	0,012	-0,543

Bacteria_Bacteroidota_Bacteroidia_Bacteroidales_Bacteroidaceae_Prevotella_Prevotella copri_A_MB2bin109	Quinolinic acid degradation_Quinolinic acid degradation_Quinolinic acid degradation	0,010	0,064	0,409
Bacteria_Bacteroidota_Bacteroidia_Bacteroidales_Barnesiellaceae_Barnesiella_Barnesiella intestinihominis_MB2bin112	Quinolinic acid degradation_Quinolinic acid degradation_Quinolinic acid degradation	0,002	0,023	-0,482
Bacteria_Bacteroidota_Bacteroidia_Bacteroidales_Barnesiellaceae_Barnesiella_Barnesiella intestinihominis_MB2bin112	Inositol degradation_Inositol degradation_Inositol degradation	0,002	0,023	0,482
Bacteria_Bacteroidota_Bacteroidia_Bacteroidales_Muribaculaceae_CAG-279_CAG-279 sp000437795_MB2bin226	Quinolinic acid degradation_Quinolinic acid degradation_Quinolinic acid degradation	0,012	0,071	0,399
Bacteria_Bacteroidota_Bacteroidia_Bacteroidales_Rikenellaceae_Alistipes_A_Alistipes_A ihumii_MB2bin204	Quinolinic acid degradation_Quinolinic acid degradation_Quinolinic acid degradation	0,010	0,064	-0,408
Bacteria_Bacteroidota_Bacteroidia_Bacteroidales_Rikenellaceae_Alistipes_Alistipes finegoldii_MB2bin46	Inositol degradation_Inositol degradation_Inositol degradation	0,000	0,011	0,555
Bacteria_Bacteroidota_Bacteroidia_Bacteroidales_Rikenellaceae_Alistipes_Alistipes obesi_MB2bin28	Inositol degradation_Inositol degradation_Inositol degradation	0,000	0,005	0,606
Bacteria_Bacteroidota_Bacteroidia_Bacteroidales_Rikenellaceae_Alistipes_Alistipes onderdonkii_MB2bin22	Inositol degradation_Inositol degradation_Inositol degradation	0,000	0,005	0,597
Bacteria_Bacteroidota_Bacteroidia_Bacteroidales_Rikenellaceae_Alistipes_Alistipes putredinis_MB2bin34	p-Cresol synthesis_p-Cresol synthesis_p-Cresol synthesis	0,000	0,008	0,605
Bacteria_Bacteroidota_Bacteroidia_Bacteroidales_Rikenellaceae_Tidjanibacter_Tidjanibacter inops_MB2bin306	Quinolinic acid degradation_Quinolinic acid degradation_Quinolinic acid degradation	0,004	0,036	-0,451
Bacteria_Firmicutes_A_Clostridia_A_Christensenellales_CAG-138_SFEL01_SFEL01 sp004557245_MB2bin134	Quinolinic acid degradation_Quinolinic acid degradation_Quinolinic acid degradation	0,004	0,036	0,453
Bacteria_Firmicutes_A_Clostridia_A_Christensenellales_CAG-74_SFFH01_SFFH01 sp900542445_MB2bin113	Inositol degradation_Inositol degradation_Inositol degradation	0,018	0,087	-0,376
Bacteria_Firmicutes_A_Clostridia_Lachnospirales_Lachnospiraceae_Anaerostipes_Anaerostipes hadrus_A_specI_v3_Cluster856	Inositol degradation_Inositol degradation_Inositol degradation	0,008	0,055	-0,419

Bacteria_Firmicutes_A_Clostridia_Lachnospirales_Lachnospiraceae_Blautia_A_?_MB2bin9	Quinolinic acid degradation_Quinolinic acid degradation_Quinolinic acid degradation	0,000	0,000	-0,663
Bacteria_Firmicutes_A_Clostridia_Lachnospirales_Lachnospiraceae_Blautia_A_Blautia_A massiliensis_MB2bin79	Inositol degradation_Inositol degradation_Inositol degradation	0,001	0,019	-0,503
Bacteria_Firmicutes_A_Clostridia_Lachnospirales_Lachnospiraceae_Blautia_A_Blautia_A sp900066335_MB2bin25	Inositol degradation_Inositol degradation_Inositol degradation	0,002	0,023	-0,482
Bacteria_Firmicutes_A_Clostridia_Lachnospirales_Lachnospiraceae_Blautia_A_Blautia_A sp900548245_MB2bin70	Inositol degradation_Inositol degradation_Inositol degradation	0,004	0,037	-0,447
Bacteria_Firmicutes_A_Clostridia_Lachnospirales_Lachnospiraceae_KLE1615_KLE1615 sp900066985_MB2bin161	Quinolinic acid degradation_Quinolinic acid degradation_Quinolinic acid degradation	0,009	0,064	0,411
Bacteria_Firmicutes_A_Clostridia_Lachnospirales_Lachnospiraceae_Mediterraneibacter_Mediterraneibacter faecis_MB2bin127	Inositol degradation_Inositol degradation_Inositol degradation	0,010	0,059	-0,409
Bacteria_Firmicutes_A_Clostridia_Lachnospirales_Lachnospiraceae_Mediterraneibacter_Mediterraneibacter lactaris_MB2bin26	Inositol synthesis_Inositol synthesis_Inositol synthesis	0,001	0,087	-0,531
Bacteria_Firmicutes_A_Clostridia_Lachnospirales_Lachnospiraceae_Mediterraneibacter_Mediterraneibacter torques_MB2bin56	Quinolinic acid degradation_Quinolinic acid degradation_Quinolinic acid degradation	0,002	0,023	-0,486
Bacteria_Firmicutes_A_Clostridia_Lachnospirales_Lachnospiraceae_Ruminococcus_A_Ruminococcus_A sp000437095_MB2bin155	Inositol degradation_Inositol degradation_Inositol degradation	0,008	0,055	-0,420
Bacteria_Firmicutes_A_Clostridia_Lachnospirales_Lachnospiraceae_Ruminococcus_A_Ruminococcus_A sp003011855_MB2bin82	Inositol degradation_Inositol degradation_Inositol degradation	0,001	0,019	-0,498
Bacteria_Firmicutes_A_Clostridia_Lachnospirales_Lachnospiraceae_UMGS1375_UMGS1375 sp900066615_MB2bin12	Inositol degradation_Inositol degradation_Inositol degradation	0,004	0,035	-0,452
Bacteria_Firmicutes_A_Clostridia_Lachnospirales_Lachnospirales_Dorea_Dorea longicatena_MB2bin81	Inositol degradation_Inositol degradation_Inositol degradation	0,005	0,043	-0,439
Bacteria_Firmicutes_A_Clostridia_Oscillospirales_Acutalibacteraceae_Eubacterium_R_Eubacterium_R sp000434995_MB2bin275	Quinolinic acid degradation_Quinolinic acid degradation_Quinolinic acid degradation	0,008	0,062	0,418

Bacteria_Firmicutes_A_Clostridia_Oscillospirales_Butyricicoccaceae_Agathobaculum_Agathobaculum sp003481705_MB2bin186	Quinolinic acid degradation_Quinolinic acid degradation_Quinolinic acid degradation	0,004	0,036	0,452
Bacteria_Firmicutes_A_Clostridia_Oscillospirales_Oscillospiraceae_CAG-170_CAG-170 sp000432135_MB2bin27	Quinolinic acid degradation_Quinolinic acid degradation_Quinolinic acid degradation	0,006	0,051	0,431
Bacteria_Firmicutes_A_Clostridia_Oscillospirales_Oscillospiraceae_CAG-170_CAG-170 sp003516765_MB2bin143	Quinolinic acid degradation_Quinolinic acid degradation_Quinolinic acid degradation	0,001	0,019	0,503
Bacteria_Firmicutes_A_Clostridia_Oscillospirales_Oscillospiraceae_CAG-83_?_MB2bin100	Acetate synthesis_Acetate synthesis II_Acetate synthesis II	0,000	0,035	0,538
Bacteria_Firmicutes_A_Clostridia_Oscillospirales_Oscillospiraceae_CAG-83_?_MB2bin100	Acetate synthesis_Acetate synthesis III_Acetate synthesis III	0,000	0,007	0,589
Bacteria_Firmicutes_A_Clostridia_Oscillospirales_Oscillospiraceae_CAG-83_?_MB2bin100	Quinolinic acid degradation_Quinolinic acid degradation_Quinolinic acid degradation	0,000	0,002	0,605
Bacteria_Firmicutes_A_Clostridia_Oscillospirales_Oscillospiraceae_CAG-83_?_MB2bin108	Quinolinic acid degradation_Quinolinic acid degradation_Quinolinic acid degradation	0,000	0,000	0,677
Bacteria_Firmicutes_A_Clostridia_Oscillospirales_Oscillospiraceae_ER4_?_MB2bin17	Quinolinic acid degradation_Quinolinic acid degradation_Quinolinic acid degradation	0,000	0,002	0,609
Bacteria_Firmicutes_A_Clostridia_Oscillospirales_Oscillospiraceae_Evtepia_?_MB2bin104	Acetate synthesis_Acetate synthesis II_Acetate synthesis II	0,001	0,058	0,504
Bacteria_Firmicutes_A_Clostridia_Oscillospirales_Ruminococcaceae_Angelakisella_Angelakisella sp900547385_MB2bin279	Quinolinic acid degradation_Quinolinic acid degradation_Quinolinic acid degradation	0,011	0,070	0,401
Bacteria_Firmicutes_A_Clostridia_Oscillospirales_Ruminococcaceae_Faecalibacterium_Faecalibacterium prausnitzii_C_MB2bin90	Inositol degradation_Inositol degradation_Inositol degradation	0,006	0,048	-0,429
Bacteria_Firmicutes_A_Clostridia_Oscillospirales_Ruminococcaceae_Faecalibacterium_Faecalibacterium prausnitzii_I_MB2bin63	Quinolinic acid degradation_Quinolinic acid degradation_Quinolinic acid degradation	0,002	0,023	0,489
Bacteria_Firmicutes_A_Clostridia_Oscillospirales_Ruminococcaceae_Faecalibacterium_Faecalibacterium sp900539945_MB2bin59	Quinolinic acid degradation_Quinolinic acid degradation_Quinolinic acid degradation	0,014	0,079	0,392
Bacteria_Firmicutes_A_Clostridia_Oscillospirales_Ruminococcacea	Inositol degradation_Inositol degradation_Inositol degradation	0,001	0,019	-0,497

e_Gemmiger_Gemmiger sp900539695_MB2bin39				
Bacteria_Firmicutes_A_Clostridia_Oscillospirales_Ruminococcaceae_Ruminococcus_D_?_MB2bin60	Quinolinic acid degradation_Quinolinic acid degradation_Quinolinic acid degradation	0,003	0,035	-0,460
Bacteria_Firmicutes_C_Negativicutes_Acidaminococcales_Acidaminococcaceae_Phascolarctobacterium_A_?_MB2bin381	Inositol degradation_Inositol degradation_Inositol degradation	0,006	0,048	-0,429
Bacteria_Firmicutes_C_Negativicutes_Acidaminococcales_Acidaminococcaceae_Phascolarctobacterium_Phascolarctobacterium faecium_MB2bin404	Inositol degradation_Inositol degradation_Inositol degradation	0,014	0,076	0,392
Bacteria_Firmicutes_C_Negativicutes_Veillonellales_Dialisteraceae_Dialister_Dialister invisus_specI_v3_Cluster3691	Inositol degradation_Inositol degradation_Inositol degradation	0,014	0,076	0,389
Bacteria_Firmicutes_I_Bacilli_A_Erysipelotrichales_Erysipelotrichaceae_Holdemanella_Holdemanella sp002299315_MB2bin140	Inositol degradation_Inositol degradation_Inositol degradation	0,000	0,005	-0,585
Bacteria_Firmicutes_I_Bacilli_A_Erysipelotrichales_Erysipelotrichaceae_Holdemanella_Holdemanella sp002299315_MB2bin140	Quinolinic acid degradation_Quinolinic acid degradation_Quinolinic acid degradation	0,003	0,033	0,464
Bacteria_Proteobacteria_Gammaproteobacteria_Enterobacterales_Enterobacteriaceae_Klebsiella_Klebsiella variicola_MB2bin427	Quinolinic acid degradation_Quinolinic acid degradation_Quinolinic acid degradation	0,008	0,062	0,417
Bacteria_Verrucomicrobiota_Verrucomicrobiae_Verrucomicrobiales_Akkermansiaceae_Akkermansia_Akkermansia muciniphila_B_MB2bin125	Inositol degradation_Inositol degradation_Inositol degradation	0,015	0,076	0,388

**Correlations in CO GBM Modules**				
**MGS**	**Pathway/GBMModule**	**p-Value**	**q-Value**	**corr-coeff**
Bacteria_Firmicutes_A_Clostridia_Lachnospirales_Lachnospiraceae_UBA11774_UBA11774 sp003507655_MB2bin41	Acetate synthesis_Acetate synthesis I_Acetate synthesis I	0,000	0,026	0,570

Supplementary Table 8

1) Have you taken antibiotics within the past three months?	(yes/no)
2) Do you regularly ingest probiotics?	(yes/no)
3) Please describe the dietary regimen you currently adhere to.	(omnivore/vegetarian/vegan)
4) Do you smoke?	(yes/no/ex-smoker)
5) Please indicate whether you consume alcohol.	(yes/no/previously)<br>If yes, how often? (<1 per month/1-4 per month/1-6 per week/1-2 per day/ ≥ 3 per day)
6) Please indicate the number of portions of dairy products consumed on a typical day.	(< 1 portion per day/2-3 portions per day/3-4 portions per day/ ≥ 5 portions per day)
7) Please indicate the number of portions of vegetables consumed on a typical day.	(< 1 portion per day/2-3 portions per day/3-4 portions per day/ ≥ 5 portions per day)

Supplementary Table 8, Dietary Questionnaire for Dietary Baseline Conditions

CONSORT 2010 checklist of information to include when reporting a pilot or feasibility trial*

Section/Topic	Item No	Checklist item	Reported on page No
**Title and abstract**			
	1a	Identification as a pilot or feasibility randomised trial in the title	1
	1b	Structured summary of pilot trial design, methods, results, and conclusions (for specific guidance see CONSORT abstract extension for pilot trials)	2
**Introduction**			
Background and objectives	2a	Scientific background and explanation of rationale for future definitive trial, and reasons for randomised pilot trial	3-4
	2b	Specific objectives or research questions for pilot trial	3-4
**Methods**			
Trial design	3a	Description of pilot trial design (such as parallel, factorial) including allocation ratio	35
	3b	Important changes to methods after pilot trial commencement (such as eligibility criteria), with reasons	Not applicable
Participants	4a	Eligibility criteria for participants	35-36
	4b	Settings and locations where the data were collected	35
	4c	How participants were identified and consented	35
Interventions	5	The interventions for each group with sufficient details to allow replication, including how and when they were actually administered	36-37
Outcomes	6a	Completely defined prespecified assessments or measurements to address each pilot trial objective specified in 2b, including how and when they were assessed	38 -42
	6b	Any changes to pilot trial assessments or measurements after the pilot trial commenced, with reasons	Not applicable
	6c	If applicable, prespecified criteria used to judge whether, or how, to proceed with future definitive trial	Not applicable
Sample size	7a	Rationale for numbers in the pilot trial	--
	7b	When applicable, explanation of any interim analyses and stopping guidelines	Not applicable
Randomisation:			
Sequence generation	8a	Method used to generate the random allocation sequence	Not applicable
	8b	Type of randomisation(s); details of any restriction (such as blocking and block size)	Not applicable
Allocation concealment mechanism	9	Mechanism used to implement the random allocation sequence (such as sequentially numbered containers), describing any steps taken to conceal the sequence until interventions were assigned	Not applicable

Implementation	10	Who generated the random allocation sequence, who enrolled participants, and who assigned participants to interventions	Not applicable
Blinding	11a	If done, who was blinded after assignment to interventions (for example, participants, care providers, those assessing outcomes) and how	Not applicable
	11b	If relevant, description of the similarity of interventions	Not applicable
Statistical methods	12	Methods used to address each pilot trial objective whether qualitative or quantitative	43-45
**Results**			
Participant flow (a diagram is strongly recommended)	13a	For each group, the numbers of participants who were approached and/or assessed for eligibility, randomly assigned, received intended treatment, and were assessed for each objective	4-5
	13b	For each group, losses and exclusions after randomisation, together with reasons	5
Recruitment	14a	Dates defining the periods of recruitment and follow-up	35
	14b	Why the pilot trial ended or was stopped	36
Baseline data	15	A table showing baseline demographic and clinical characteristics for each group	5<br>Suppl. table 1
Numbers analysed	16	For each objective, number of participants (denominator) included in each analysis. If relevant, these numbers should be by randomised group	45<br>Fig. 1a
Outcomes and estimation	17	For each objective, results including expressions of uncertainty (such as 95% confidence interval) for any estimates. If relevant, these results should be by randomised group	Main Figures
Ancillary analyses	18	Results of any other analyses performed that could be used to inform the future definitive trial	Not applicable
Harms	19	All important harms or unintended effects in each group (for specific guidance see CONSORT for harms)	6
	19a	If relevant, other important unintended consequences	Not applicable
**Discussion**			
Limitations	20	Pilot trial limitations, addressing sources of potential bias and remaining uncertainty about feasibility	33-34
Generalisability	21	Generalisability (applicability) of pilot trial methods and findings to future definitive trial and other studies	34
Interpretation	22	Interpretation consistent with pilot trial objectives and findings, balancing potential benefits and harms, and considering other relevant evidence	27,29-30,33-34
	22a	Implications for progression from pilot to future definitive trial, including any proposed amendments	27,29-30,33-34
**Other information**			
Registration	23	Registration number for pilot trial and name of trial registry	35
Protocol	24	Where the pilot trial protocol can be accessed, if available	Not applicable
Funding	25	Sources of funding and other support (such as supply of drugs), role of funders	53

	26	Ethical approval or approval by research review committee, confirmed with reference number	35

Citation: Eldridge SM, Chan CL, Campbell MJ, Bond CM, Hopewell S, Thabane L, et al. CONSORT 2010 statement: extension to randomised pilot and feasibility trials. BMJ. 2016;355. This is an Open Access article distributed in accordance with the terms of the Creative Commons Attribution (CC BY 3.0) license (http://creativecommons.org/licenses/by/3.0/), which permits others to distribute, remix, adapt and build upon this work, for commercial use, provided the original work is properly cited.

*We strongly recommend reading this statement in conjunction with the CONSORT 2010, extension to randomised pilot and feasibility trials, Explanation and Elaboration for important clarifications on all the items. If relevant, we also recommend reading CONSORT extensions for cluster randomised trials, non-inferiority and equivalence trials, non-pharmacological treatments, herbal interventions, and pragmatic trials. Additional extensions are forthcoming: for those and for up-to-date references relevant to this checklist, see www.consort-statement.org.
